# Lienhwalides: Unique Tropolone–Maleidride Hybrids from *Hypoxylon lienhwacheense*

**DOI:** 10.1002/cbic.202500037

**Published:** 2025-04-07

**Authors:** Katharina Schmidt, Esteban Charria‐Girón, Tatiana E. Gorelik, Christian Kleeberg, Jackson M. Muema, Simone Heitkämper, Bart Verwaaijen, Eric Kuhnert, Jennifer Gerke, Jörn Kalinowski, Kevin D. Hyde, Marc Stadler, Russell Cox, Frank Surup

**Affiliations:** ^1^ Institute for Organic Chemistry and BMWZ Leibniz Universität Hannover Schneiderberg 38 30167 Hannover Germany; ^2^ Department Microbial Drugs Helmholtz Centre for Infection Research (HZI), and German Centre for Infection Research (DZIF) Partner Site Hannover‐Braunschweig Inhoffenstrasse 7 38124 Braunschweig Germany; ^3^ Institute of Microbiology Technische Universität Braunschweig Spielmannstraße 7 38106 Braunschweig Germany; ^4^ Department of Structure and Function of Proteins Helmholtz Centre for Infection Research (HZI) Inhoffenstraße 7 38124 Braunschweig Germany; ^5^ Ernst Ruska‐Centre for Microscopy and Spectroscopy with Electrons (ER‐C) Forschungszentrum Jülich 52425 Jülich Germany; ^6^ Institute for Inorganic and Analytical Chemistry Technische Universität Braunschweig Hagenring 30 38106 Braunschweig Germany; ^7^ Department of Compound Profiling and Screening Helmholtz Centre for Infection Research (HZI), and German Centre for Infection Research (DZIF) Partner Site Hannover‐Braunschweig Inhoffenstrasse 7 38124 Braunschweig Germany; ^8^ CeBiTec University of Bielefeld Universitätsstraße 27 D‐33615 Bielefeld Germany; ^9^ Institute of Excellence in Fungal Research Mae Fah Luang University Chiang Rai 57100 Thailand

**Keywords:** antibiotics, biosyntheses, genomics, metabolomics, secondary metabolites, structure elucidations

## Abstract

*Hypoxylon lienhwacheense*, a fungal species with an unclear taxonomic placement within the Hypoxylaceae, presents a highly rare stromatal secondary metabolite profile. Isolation of its major stromatal constituents leads to the discovery of a novel tropolone–maleidride hybrid molecule, lienhwalide A **5**, in addition to the known cordyanhydride B **6**, its new derivative **7**, and binaphthalenetetraol **8**. Unexpectedly, *Hypoxylon lienhwacheense* produces in liquid cultures various lienhwalide A congeners **9**–**11**. Their structures and relative configurations are elucidated using high‐resolution mass spectrometry and nuclear magnetic resonance (NMR) spectroscopy, with their absolute configurations determined using X‐ray analysis of a semisynthetic brominated derivative of **9** and synthesizing α‐methoxy‐α‐trifluoromethylphenylacetyl esters of **11**. Feeding experiments with ^13^C‐labeled precursors (^13^C‐methionine; 1‐^13^C‐ and U‐^13^C_6_‐glucose) reveal insights into the biogenesis of tropolone and maleidride moieties, according to ^13^C couplings and incredible natural abundance double quantum transfer NMR data. Genome analysis identifies two separate biosynthetic gene clusters responsible for these moieties, and heterologous expression experiments provide further insights into the interplay of both clusters during the biosynthesis of these hybrid natural products. Remarkably, lienhwalides exhibit reduced toxicity and enhance antibacterial selectivity compared to related fungal tropolones.

## Graphical abstract

Lienhwalides are novel tropolone–maleidride hybrid molecules unique in nature. Absolute configuration is solved by semisynthetic approaches and X‐ray analysis. Interplay between both *lwt* and *lwm* biosynthetic gene clusters is responsible for the production of lienhwalides.

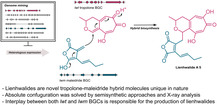

## 1 Introduction

The Xylariales constitute one of the largest and most intriguing orders of the Sordariomycetes. Their rather interesting ecology confers them an important role as prolific secondary metabolite producers. From its second largest family (Hypoxylaceae) alone, several hundreds of secondary metabolites have already been reported.^[^ ^1^, ^2^ ^]^ Examples of these bioactive metabolites include the insecticidal nodulisporic acids (*Hypoxylon pulicicidum*);^[^ ^3^, ^4^ ^]^ antifungal sporothriolides (*Hypomontagnella* spp.);^[^ ^5^, ^6^, ^7^ ^]^ cytochalasans (*Hypoxylon. fragiforme and Daldinia spp.*);^[^ ^8^ ^]^ the topoisomerase I inhibitor hypoxyxylerone (*H. fragiforme*);^[^ ^9^ ^]^ immunosuppressive polyketides dalesconols A and B (*Daldinia eschscholtzii*);^[^ ^10^ ^]^ phytotoxic eutypine derivatives (*Phylacia sagrana*);^[^ ^11^ ^]^ rubiginosin C that is an inhibitor of hyphae and biofilm formation;^[^ ^12^ ^]^ and multiformin‐type azaphilones that inhibit the binding of severe acute respiratory syndrome coronavirus 2 (SARS–CoV‐2) spike protein to mammalian angiotensin converting enzyme 2 receptors.^[^ ^13^ ^]^ Single family members of the Hypoxylaceae also produce highly diverse mixtures of bioactive metabolites. For example, fermentation of *Hypoxylon rickii* in a 70 L bioreactor revealed the production of 31 compounds derived from eight different core scaffolds, including sesquiterpenoids,^[^ ^14^ ^]^ diterpenes,^[^ ^15^ ^]^ macrolactones,^[^ ^16^ ^]^ and terphenyls.^[^ ^17^ ^]^ This extensive chemical diversity has significant implications for drug discovery and also serves as a reliable tool for taxonomic investigations. Metabolites found in the stromata of the Hypoxylaceae have proven to be highly conserved, often at the genus and even species level, irrespective of their origin and environmental conditions.^[^ ^18^, ^19^, ^20^ ^]^

Recently, 13 taxonomically well‐defined representatives of the family, and one member of the closely related Xylariaceae (*Xylaria hypoxylon*), were genome sequenced using a combination of third‐generation sequencing technologies.^[^ ^21^ ^]^ This study provided a solid backbone for genomic investigations of the Hypoxylaceae for the first time, confirming the findings of previous phylogenetic studies.^[^ ^18^, ^21^ ^]^ Inspired by the diversity of secondary metabolites within the Hypoxylaceae and the availability of high‐quality genome sequences, the biosynthetic potential of these organisms was investigated to estimate their true potential as secondary metabolite producers.^[^ ^22^ ^]^ This led to the identification of 783 biosynthetic pathways across the fourteen studied species, mostly organized in biosynthetic gene clusters (BGCs), which constituted 375 gene cluster families (GCFs). Notably, only 10 GCFs were found to be conserved across all these fungi, underlining the fact that speciation in the Hypoxylaceae entails changes in their secondary metabolism.

**Figure 1:**
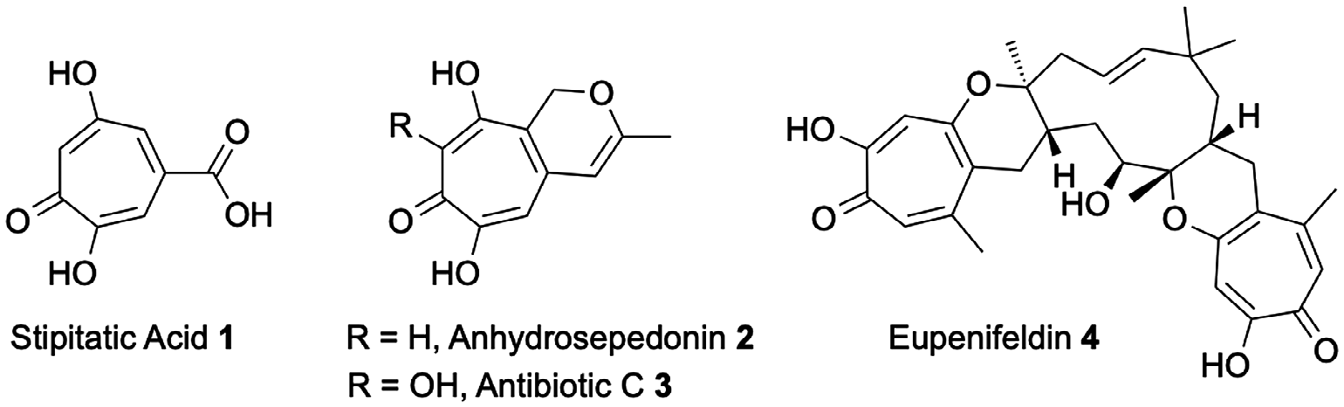
Fungal tropolones.

Among the identified GCFs, a tropolone pathway^[^ ^23^, ^24^ ^]^ was found to be present in 13 of the 14 studied species. This observation was intriguing since the common presence of this BGC suggests that the encoded tropolone metabolite may have an important ecological role. However, no tropolones have yet been reported as metabolites of this family. Tropolones are relatively rare in fungi,^[^ ^25^, ^26^ ^]^ but notable examples include stipitatic acid **1**;^[^ ^27^ ^]^ anhydrosepedonin **2** and antibiotic C **3**;^[^ ^24^ ^]^ and the tropolone meroterpenoids such as eupenifeldin **4** (**Figure** **1**).^[^ ^28^, ^29^ ^]^ Here, we report the isolation, structure elucidation, biological testing, and preliminary biosynthetic investigations of the lienhwalides, unique new hybrid tropolone–maleidrides^[^ ^30^ ^]^ from *Hypoxylon lienhwacheense*.

## 2 Results

**Figure 2:**
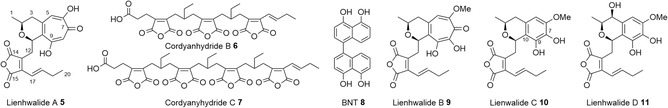
Chemical structures of isolated metabolites from the stromata (**5**–**8**) and scaled‐up cultivation in YM 6.3 liquid medium (**9**–**11**) of *H. lienhwacheense.*

*Hypoxylon lienhwacheense* has an indistinct taxonomic position within the fungal family Hypoxylaceae as revealed by previous phylogenetic and phylogenomic analyses using high‐quality genome sequences generated by third‐generation sequencing methods.^[^ ^21^, ^31^ ^]^ Similarly, the metabolite profile of its stromata is unique when compared with previously investigated Hypoxylaceae. High‐resolution electrospray ionization mass spectrometry (HR–ESI–MS) analysis of the stromatal extract revealed the presence of three major peaks (Figure S1, Supporting Information), from which the hypoxylaceous chemotaxonomic marker binaphthalenetetraol (BNT, **8**) was identified, while the other two compounds appeared to be new in relation to the closest relatives of *H. lienhwacheense*. Therefore, fresh stromata of this species (3.54 g) were extracted, and the obtained crude extract (503 mg) purified by preparative HPLC, resulting in the isolation of compounds **5**–**8** (**Figure** **2**).

The molecular formula of **5** was determined as C_20_H_20_O_7_ based on the *m/z* peak at 373.1281 in the HR–ESI–MS spectrum, indicating eleven units of unsaturation. ^1^H and heteronuclear single quantum coherence (HSQC), nuclear magnetic resonance (NMR), α‐methoxy‐α‐trifluoromethylphenylacetyl spectra (see ESI for full NMR spectra) revealed the presence of two methyls, three methylenes as well as two oxymethines and four olefinic methines. The ^13^C NMR spectrum displayed a further nine quaternary sp^2^‐hybridized carbon atoms, of which five were deshielded having chemical shifts between 161 and 174 ppm. Correlation spectroscopy (COSY) and total correlation spectroscopy (TOCSY) spectra connected the three spin systems 1–H_3_/2–H/3–H_a_/3–H_b_, 11–H/12–H_a_/12–H_b_, and 17–H/18–H/19–H_2_/20–H_3_. These spin systems were linked by heteronuclear multiple bond correlation (HMBC) correlations to a continuous carbon chain including carbons C–4, C–10, C–13, and C–16. The ether bridge between C–2 and C–11 was deduced from mutual HMBC correlations. The locations of the two carboxyls C–14 and C–15 were determined by HMBC correlations of 12–H_2_ to C–14 and 17–H to C–15, respectively. HMBC correlations of 5–H to C–6/C–7 and 9–H to C–7/C–9 confirmed the tropolone moiety. Finally, the missing degree of unsaturation requires an anhydride linkage between C–14 and C–15 that is consistent with the chemical shifts of these carbons.

The relative configuration between 2–H and 11–H was deduced as *cis* based on the rotating‐frame overhauser enhancement spectroscopy (ROESY) correlation between these two protons. Cordyanhydride B **6** and BNT **8** were identified by comparison of their NMR data to the literature. The NMR data of cordyanhydride C **7** was very similar to those of **6**. However, an additional maleidride subunit was deduced from the molecular formula C_38_H_42_O_14_ and NMR data.

**Scheme 1:**
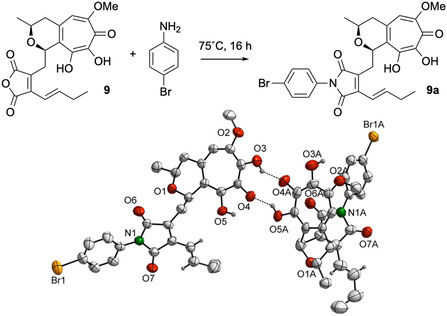
4‐*p*‐bromoaniline derivatization of **9** to obtain **9a**.

Subsequently, a fermentation of *H. lienhwacheense* in liquid yeast extract‐malt extract medium (YM 6.3, 4L) was carried out and the obtained crude extracts from the supernatant and the mycelia were checked for the production of other tropolone maleidrides related to compound **5** (Figure 2). Several derivatives of **5** were detected by HR–ESI–MS and their distinctive UV/vis absorption at *λ*_max_ 216, 254, and 326 nm. Targeted purification of these compounds by preparative HPLC resulted in the isolation of compounds **9**–**11**. Compound **6** was produced only in traces and barely detected by HPLC–MS analysis, and therefore not isolated. Tropolone **9** was analyzed by HR–ESI–MS and its molecular formula determined as C_21_H_22_O_8_, indicating the formal addition of a CH_2_O unit compared to **5**. NMR spectra of **9** were highly similar to those of **5**, with the key changes being the absence of methine 9–H and the presence of an oxymethyl group. This oxymethyl group is attached to C–6, as HMBC correlations of both 6–OCH_3_ and 5–H to C–6 show. C–8 is hydroxylated in comparison to **5**, as shown by the HMBC correlation of 11–H to C–9. Analogously to **5**, ROESY correlations between 2–H and 11–H confirm a *cis* configuration of these protons. Finally, a derivatization approach was undertaken to allow the establishment of the absolute configuration of these metabolites by X‐ray analysis. The reaction of **9** with 4‐bromonaniline yielded the crystalline derivative **9a** (**Scheme** **1**), which was subjected to crystallographic analysis. The obtained single‐crystal X‐ray structure of **9a** confirmed the proposed structure of **9** and the 2*S*, 11*R* absolute configuration.

Compound **10** has the molecular formula C_20_H_22_O_7_ and very similar NMR spectra to **9**. However, chemical shifts and HMBC correlations confirm that the tropolone of **9** is replaced by a benzene. Analogously to **9**, a ROESY correlation between 2–H and 11–H was observed for **10**, indicating the same relative stereochemistry. Finally, **11** was elucidated as the 3‐hydroxy derivative of **10**. Synthesis of *R*‐ and *S*‐α‐methoxy‐α‐trifluoromethylphenylacetyl esters of **11** confirmed the absolute configuration of the molecules, since Δ*δ* ^SR^ shifts were positive for 1–H_3_(+0.18), but negative for 5–H(−0.06).

### 2.1 Genome Sequence and Analysis

**Figure 3:**
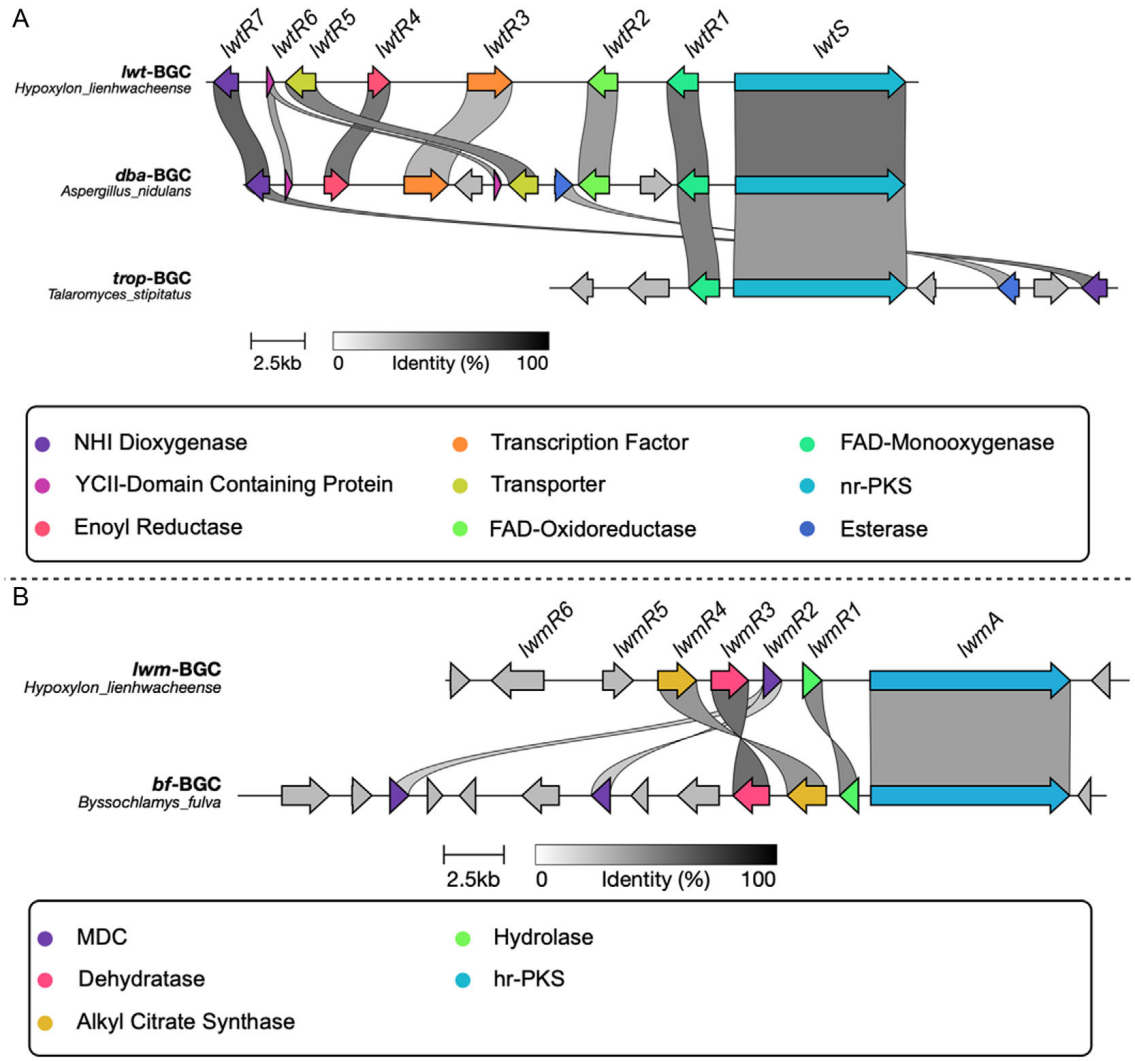
BGC analysis. A) Comparison of the *H. lienhwacheense* tropolone BGC (*lwt*) to the anhydrosepedonin BGC (*dba*) of *A. nidulans* and stipitatic acid BGC (*trop*) from *T. stipitatus*; B) comparison of the *H. lienhwacheense* maleidride BGC (*lwm*) with the byssochlamic acid (*bf*) BGC from *B. fulva*.

Genomic DNA (gDNA) from *H. lienhwacheense* strain MFLUCC 14‐1231 was prepared and sequenced using a combination of Illumina and Oxford nanopore technologies.^[^ ^21^ ^]^ The resulting assembled sequences (38.5 Mbp, N50 1.6 Mbp, 61 contigs) were used for gene prediction by an established pipeline, revealing 9942 open reading frames (ORFs) and a total of 25 BGCs.^[^ ^22^ ^]^ Analysis of the assembled genome revealed the presence of a single‐tropolone BGC that is highly homologous to the well‐understood *dba* tropolone BGC from *Aspergillus nidulans* that encodes the biosynthesis of anhydrosepedonin **2** and antibiotic C **3** (**Figure** **3**A).^[^ ^24^ ^]^

In particular, genes encoding a tetraketide synthase (*lwtS*), a flavin adenine dinucleotide‐dependent monooxygenase (*lwtR1*), and a non‐heme iron dioxygenase (*lwtR7*) also have close homologs in the well‐understood stipitatic acid **1** pathway in *Talaromyces stipitatus* (Table S7, Supporting Information).^[^ ^23^ ^]^

Two putative maleidride‐type BGCs were identified. One of these (*lwm*) had five genes in common with the *Byssochlamys fulva* metabolite byssochlamic acid **14** BGC (*bf*, Figure 3B).^[^ ^30^ ^]^ These encode an iterative highly reducing polyketide synthase (PKS, *lwmA*), a hydrolase (*lwmR1*), a maleidride dimerizing cyclase (MDC, *lwmR2*),^[^ ^32^ ^]^ a 2‐methylcitrate dehydratase (2MCDH, *lwmR3*) homolog, and an alkyl citrate synthase (*lwmR4*). In addition, *lwmR6* encodes a putative ATP‐dependent CoA ligase and *lwmR7* encodes another putative hydrolase (**Table** **1**). The *bf* BGC encodes a protein with homology to phosphatidylethanolamine binding proteins (PEBP) that may be involved in dimerization of monomers during the biosynthesis of nonandrides.^[^ ^32^ ^]^ However, the *lwm* BGC does not appear to encode any identifiable PEBP proteins. The second *H. lienhwacheense* maleidride‐like BGC shows higher homology to the oryzine‐type maleidride BGCs that encode fungal fatty acid synthase components rather than PKSs.^[^ ^33^ ^]^

**Table 1:** Minimum inhibitory concentration (MIC, μg mL^−1^) against bacterial test organisms, half‐maximal inhibitory concentrations (IC_50_, μg mL^−1^) against mammalian cell lines, and single‐dose assay against CHIKV.

Tested organisms/cell line	Code	Compound							References
		3*	**5**	**6**	**8**	**9**	**10**	**11**	
MIC versus bacteria [μg mL^−1^]									
*Bacillus subtilis*	DSM 10	33.3	33.3	–	50	16.6	–	–	8.3a)
*Escherichia coli*	DSM 1116	33.3	–	n.d.	n.d.	–	–	–	1.7b)
*Pseudomonas aeruginosa*	PA 14	–	–	n.d.	n.d.	–	–	–	0.21b)
*Streptococcus aureus*	DSM 346	16.6	33.3	50	25	16.6	–	–	0.4b)
*Chromobacterium violaceum*	DSM 30 191	8.3	–	–	50	33.3	–	–	0.42b)
*Acinetobacter baumannii*	DSM 30 008	16.6	–	n.d.	n.d.	–	–	–	0.26c)
*Mycobacterium smegmatis*	ATCC 700 084	33.3	–	n.d.	n.d.	–	–	–	1.7d)
IC_50_ versus mammalian cell lines [μg mL^−1^]									
KB‐3‐1	ACC 158	0.089	26	–	–	14	–	–	5.8 × 10^−5^ e)
L929	ACC 2	0.085	26	–	–	7.5	–	–	9.0 × 10^−4^ e)
A549	ACC 107	0.080	n.d.	n.d.	n.d.	8	n.d.	n.d.	1.2 × 10^−4^ e)
SK‐OV‐3	ACC HTB 77	0.084	n.d.	n.d.	n.d.	6.3	n.d.	n.d.	1.4 × 10^−4^ e)
Cell viability [%]									
Anti‐CHIKV		n.d.	19	n.d.	n.d.	15	–	–	100f), g)

a) Oxytetracycline.

b) Gentamicin.

c) Ciprofloxacin.

d) Kanamycin.

e) Epothilone B.

f) Ribavirin.

g) Notes: no activity observed under test conditions (–), not tested (n.t.), and data extracted from previous studies (*).^[^ ^24^ ^]^

### 2.2 Biosynthetic Investigations

We carried out a series of feeding experiments with isotope‐labeled precursors. ^13^C‐enrichments of main metabolite **9** were analyzed by ^13^C NMR spectroscopy. Unfortunately, feeding of acetate, which is the most likely direct precursor, caused a collapse of lienhwalide production. Thus, 1‐^13^C‐ and *u*‐^13^C_6_‐glucose were fed since labeled glucose is converted into labeled acetate *via* the Krebs cycle. Feeding of 1‐^13^C‐glucose (equivalent to 2‐^13^C‐acetate), resulted in high incorporation rates at positions C–1, C–3, C–5, C–7, C–10, C–14, C–16, C–18, and C–20. Feeding of *u*‐^13^C_6_‐glucose showed the intact incorporation of the units C–1/C–2, C–3/C–4, C–5/C–6, C–7/C–9, C–10/C–11, C–12/C–13/C–14, C–15/C–16, C–17/C–18, and C–19/C–20. Notably, both incredible natural abundance double quantum transfer NMR correlations as well as mutual coupling constants in the ^13^C spectrum of neighboring carbons confirmed the incorporation of the C–12/C–13/C–14 and C–7/C–9 as intact units. Finally, feeding of *S*‐methyl‐^13^C‐labeled methionine resulted in the signal enhancement of C–8 and 6–OCH_3_.

The function of the *lwm* BGC was investigated by heterologous expression in *Aspergillus oryzae*. Genes were amplified by PCR from cDNA prepared from a producing strain of *H. lienhwacheense* (i.e., the templates lacked introns). Genes encoding the PKS, hydrolase, citrate synthase, and 2MCDH (*lwmA*, *lwmR1*, *lwmR*4, and *lwmR3*, respectively) were cloned into a fungal expression vector with the *adeA* selection gene. In this system, *lwmA* is driven by a dextrin‐inducible promoter *P*_amyB_, while the other genes are driven by strong constitutive promoters (see ESI for details).^[^ ^34^ ^]^ *A. oryzae* NSAR1 (that contains four auxotrophic lesions in *adeA*, *argB*, *sC*, and *niaD*)^[^ ^35^ ^]^ was transformed with this vector to give *A. oryzae* *lwmA·R1·R3·R4*. Sixteen transformants were selected on media lacking adenine and then grown in dextrin–polypeptone–yeast extract (DPY) media. LCMS analysis of the transformants versus untransformed *A. oryzae* NSAR1 (Figure S9, Supporting Information) showed the clear presence of a new peak in four of the transformants (Figure S9, Supporting Information). Initial LCMS data suggested this to be **19a**, but relatively low titers hindered purification and full NMR determination at this stage.

The gene *lwmR6* encodes an ATP‐dependent CoA ligase (Figure 3B). It has been established in vitro that alkyl citrate synthases like LwmR4 only accept CoA thiolester substrates.^[^ ^36^ ^]^ Fungal iterative hrPKS/hydrolase systems release carboxylic acids such as **15** rather than CoA thiolesters such as **16**,^[^ ^37^ ^]^ so we hypothesized that *lwmR6* may encode the required thiolester synthetase. The gene *lwmR6* was therefore cloned in an *argB*‐selectable vector and integrated into the genome of *A. oryzae* with the previous four genes to give *A. oryza*e *lwmA·R1·R3·R4·R6*. Seventeen transformants were selected on media lacking adenine and arginine and again grown in DPY media. In this case, 13 of the transformants produced the same compound **19a**, but in significantly higher amounts and accompanied by two later‐eluting compounds **20a** and **21a** (Figure S9, Supporting Information).

The main component was purified and shown by NMR analysis to be the expected maleidride monomer **19a**.^[^ ^37^ ^]^ The later‐eluting component **20a** proved to be the known decarboxylation product of **19a**. Surprisingly, purification and full NMR and high‐resolution mass spectrometry analysis of the third compound proved it to be the methylene homolog **21a** (ESI).

Cordyanhydrides B **6** and C **7** are produced by wild type (WT) *H. lienhwacheense*, but the heterologous expression experiments in *A. oryzae* with *lwmA·R1·R3·R4·R6* did not produce these compounds, despite producing the apparent precursors **19a** and **21a**. Cordyanhydride B **6** appears to be composed of two **19a**‐derived maleic anhydrides, linked to a unit potentially derived from **21a**. In the case of byssochlamic acid **14** and other nonadrides, the MDCs encoded by *bfl6* and *bfl10* have been linked to the dimerization (and cyclization) of **19a** both in vivo and in vitro.^[^ ^38^ ^]^

We therefore added the MDC component encoded by *lwmR2* to the expression system to determine if it has a role in biosynthesis of **6** and **7**. PEBP components are also implicated in this chemistry,^[^ ^37^ ^]^ but the *lwm* BGC does not encode a PEBP homolog. However, *lwmR5* encodes a protein of unknown function and we also included this for completeness. Thus *lwmR2* and *lwmR5* were cloned into the *argB*‐selective vector previously used for *lwmR6*. This was integrated in parallel to the adenine selective vector already described to give *A. oryzae* strains containing the full *lwm* BGC (*lwmA·R1·R2·R3·R4·R5·R6*) that were selected on media lacking arginine and adenine. Twenty transformants were selected and grown in liquid DPY media, but none produced any compounds in addition to the already observed **19a**, **20a**, and **21a** (see ESI).

The observation of **21a**, that possesses an additional methylene group compared to **19a**, raised an interesting possibility that the LwmR4 alkyl citrate synthase might accept the five‐carbon α‐ketoglutaric acid **22** in addition to the four‐carbon oxaloacetic acid **17** as a substrate. To test this possibility we investigated the selectivity of LwmR4 in vitro. The gene *lwmR4* was synthesized in codon‐optimized form and expressed from pET28a(+) in *E. coli* BL21 (DE3) using standard protocols. The his_6_‐tagged protein was purified from the lysed cells in one step using nickel–ion affinity chromatography to give pure protein of the expected 49 kDa. In vitro reactions were set up with hex‐3‐enoyl CoA **16a** or hexanoyl CoA **16b** (Scheme S9, Supporting Information) and either oxaloacetic acid **17** or α‐ketoglutaric acid **22**. The reaction mixtures were incubated at 30 °C for 2 h. Protein was precipitated with 1 equivalent of acetonitrile and removed by centrifugation. The assay supernatant was then analyzed directly by LCMS. In the case of oxaloacetic acid **17**, the expected alkyl citrate product **18a** was clearly observed (Figure S9 and ESI, Supporting Information), but in the α‐ketoglutaric acid **22** reactions, no new products were observed (Figure S9 and ESI, Supporting Information).

### 2.3 Biological Activity of Lienhwalides

We investigated the antimicrobial, cytotoxic, and antiviral properties of the isolated lienhwalides (Table 1). Inhibition of both Gram‐positive and Gram‐negative bacteria was observed for the tropolones. For instance, lienhwalide B **9** inhibited the Gram‐negative *Chromobacterium violaceum*, with an MIC value of 33.3 μg mL^−1^. In contrast, lienhwalide A **5**, which is the C‐8‐deoxy and C‐6‐hydroxy derivative of **9**, did not display antibacterial properties against *C. violaceum*. Both tropolone‐containing molecules exhibited moderate inhibition of the Gram‐positive bacteria *Bacillus subtilis* and *Staphylococcus aureus*. However, none of the congeners inhibited the growth of fungal test organisms.

Similarly, all tropolone‐containing lienhwalides demonstrated varying degrees of cytotoxic effects. Compound **9** displayed moderate cytotoxicity against both KB‐3.1 and L929 cell lines. Consequently, these molecules were evaluated against A549 and SKOV‐3 cell lines, where they demonstrated similar cytotoxic effects. In general, the presence of an *O*‐methyl group at C‐6 and a hydroxyl group at C‐8 appears to be associated with higher antibacterial and cytotoxic properties. To further evaluate the biological properties of the isolated lienhwalides, we tested their antiviral properties against the chikungunya virus (CHIKV). Only compounds **5** and **9** displayed rather weak anti‐CHIKV activities. However, in contrast to the antimicrobial and cytotoxicity assays, in this assay, compound **5** showed more potent effects than **9**.

The biological activities of the lienhwalides were then compared to antibiotic C **3**, an antibacterial and iron‐chelating tropolone, which is the product of the highly homologous *dba* BGC. In fact, antibiotic C **3–** and anhydrosepedonin **2**–derived molecules display strong antimicrobial effects against several microorganisms but also exhibit potent cytotoxicity against mammalian cell lines. It is notable that the tropolone‐containing lienhwalides retain their activity against Gram‐positive bacteria and against the Gram‐negative *C. violaceum* but lose the activity against *Acinetobacter baumannii*, *Mycobacterium smegmatis*, and fungal pathogens. In addition, all lienhwalides exert at least ≈100‐fold lower cytotoxicity against the tested cell lines compared to antibiotic C **3**. Similarly, when comparing tropolone‐containing lienhwalides to compound **6**, it is evident that the antibacterial properties might be attributed to the tropolone moiety, as aromatic lienhwalides lack antimicrobial or cytotoxic effects. The ring‐contracted lienhwalides **10** and **11** showed no activity in any of the assays tested. All the aforementioned suggest that the conjugation of the tropolone and anhydride moieties retains potent antibacterial effects with reduced toxicity to mammalian cells.

## 3 Discussion and Conclusion

The lienhwalides **5** and **9** are structurally unique fungal metabolites that consist of conjoined tropolone and maleidride units. The results of isotope feeding experiments support this hypothesis. Mining of the *H. lienhwacheense* genome revealed two separate BGCs that are highly likely to be involved. The *lwm* BGC was shown to produce the required maleidride component **19a** that is also known from byssochlamic acid **14** biosynthesis. In addition, the methylene homolog **21a** was also produced in vivo. Compound **21a** could be involved in the biosynthesis of the cordyanhydrides B **6** and C **7**. As expected, the citrate synthase component LwmR4 can condense the polyketide **16a** with oxaloacetic acid **17** to give the known precursor **19a** in vitro. However, the precise origin of **21a** remains mysterious as it cannot be produced directly from the polyketide **16a** and α‐ketoglutarate **22** in vitro.

**Scheme 2:**
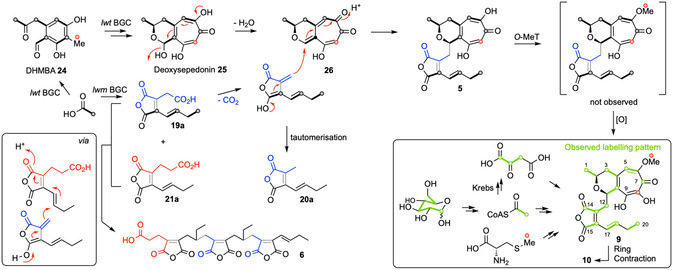
Biosynthetic hypotheses for the lienhwalides and cordyanhydrides.

The *lwt* BGC is highly homologous to the known BGCs involved in stipitatic acid **1** biosynthesis in *T. stipitatus* ^[^ ^23^ ^]^ and anhydrosepedonin **2** biosynthesis in *A. nidulans*.^[^ ^24^ ^]^ Since the *lwt* BGC is the only tropolone BGC found on the genome of *H. lienhwacheense*, it is highly likely to provide the tropolone moiety during biosynthesis. The pentaketide tropolone pathway is known to proceed *via* DHMBA **24** and may involve the known deoxysepedonin **25** (**Scheme** **2**).

Our heterologous expression experiments proved production of the key maleidride intermediate **19a**, but they were unable to answer questions regarding the linkage of the tropolone and maleidride moieties, or the biosynthesis of the cordyanydrides **6** and **7**, since expression of further genes from the *lwm* BGC in *A. oryzae* did not provide these compounds. However, our hypothesis for future investigations involves decarboxylation of **19a** and nucleophilic attack of the resulting enol(ate) on an electrophilic tropolone species—possibly a quinomethide such as **26** (Scheme 2). Similar chemistry is already known to be involved during the biosynthesis of nonadrides such as byssochlamic acid **14** ^[^ ^30^ ^]^ and tropolone quinomethides are implicated during the biosynthesis of tropolone meroterpenoids.^[^ ^39^ ^]^

We also propose similar chemistry to explain the biosynthesis of the cordyanhydrides where the same nucleophilic species derived from **19a** attacks a maleidride monomer such as **21a** or multimer, such as **6** itself, to produce **7**. Enzymes known as MDCs have been implicated in similar chemistry, but although an MDC is encoded in the *lwm* BGC, its expression in *A. oryzae* did not lead to the production of the cordyanhydrides or other new compounds in the presence of **19a** and **21a**.

Genes encoding the biosynthesis of fungal metabolites are almost always clustered together on genomes to provide easily recognized BGCs. This is usually also the case for metabolites derived from different biosynthetic classes. For example, fungal meroterpenoid biosynthesis BGCs normally encode the biosynthesis of the terpene and polyketides themselves, the linking functionality, and the tailoring enzymes.^[^ ^40^ ^]^ However, it is rarely observed that BGCs can be split into two or more loci. For example, prenylxanthone biosynthesis in *Aspergillus nidulans* involves genes from two separate loci.^[^ ^41^, ^42^ ^]^ Likewise, echinocandin B biosynthesis in *Aspergillus rugulosus* (syn. *Emericella rugulosa*) appears to be catalyzed by enzymes encoded by two separate BGCs^[^ ^43^ ^]^ while the biosynthesis of dothistromin in *Dothistroma septosporum* appears to be directed by three separate genetic loci.^[^ ^44^ ^]^ In the case of the lienhwalides, it appears that biosynthesis involves the interaction of tropolone and maleidride BGCs that are already well understood. However, key catalytic components do not appear to be encoded within the *lwt* and *lwm* BGCs. For example, *O*‐methylation of **5** to produce **9** would likely require an S‐adenosyl methionine‐dependent *O*‐methyltransferase. Hydroxylation of the tropolone ring to produce triol **9** would require a redox‐active enzyme such as the FAD‐dependent AsL4 and AsL6 oxygenases known from the xenovulene pathway.^[^ ^28^, ^39^ ^]^ These oxidative enzymes also appear to catalyze ring contraction reactions that could explain the presence of **11** and the other benzenoid congeners in *H. lienhwacheense*. However, such enzymes do not appear to be encoded in the *lwt* or *lwm* BGCs and further investigations will be required to find them. Likewise, the annotated MDC enzyme does not appear functional alone (at least in *A. oryzae*) and so may require another catalytic partner such as a PEBP‐type protein (also not encoded by the *lwt* and *lwm* BGCs) encoded elsewhere on the genome. The origin of the intriguing connection between the tropolone and maleidride components also remains speculative and further work will be required to elucidate this step. Although the cross talk mechanism between these BGCs remains unclear, it appears to confer an evolutionary advantage which might be related to an as‐yet uncovered ecological function, as lienhwalides retained their antibacterial activity while exhibiting reduced toxicity to mammalian cells. This suggests a natural optimization strategy for fungal tropolones, potentially reflecting their role in ecological competition or defense mechanisms.

## Supporting Information

Full supporting information including: all experimental methodology and full compound characterisation is available online.

## Conflict of Interest

The authors declare no conflict of interest.

## Author Contributions

**Katharina Schmidt** and **Esteban Charria‐Girón** contributed equally to this work.

## Supporting information

Supplementary Material

## Data Availability

The data that support the findings of this study are available from the corresponding author upon reasonable request.
