# Supplementary material for: Lienhwalides: Unique Tropolone–Maleidride Hybrids from Hypoxylon lienhwacheense
Source: Chembiochem. 2025 Apr 7;26(10):e202500037. doi: 10.1002/cbic.202500037 (PMC12117428; doi:10.1002/cbic.202500037)
Supplement: Supplementary file 1 — Supplementary Material [file CBIC-26-e202500037-s001.pdf]

Lienhwalides: Unique Tropolone-Maleidride Hybrids from *Hypoxylon lienhwacheense*

Katharina Schmidt, +[a] Esteban Charria-Girón,+[b,c] Tatiana E. Gorelik, [d,e] Christian Kleeberg[f], Jackson M. Muema,[g] Simone Heitkemper,[b] Bart Verwaaijen,[h] Eric Kuhnert,[a] Jennifer Gerke,[a] Jörn Kalinowski,[h] Kevin D. Hyde,[i] Marc Stadler,[b,c] Russell Cox*[a] and Frank Surup*[b,c]

[a] Dr. K. Schmidt, Dr. E. Kuhnert, Dr. J. Gerke, Prof. R. J. Cox
Institute for Organic Chemistry and BMWZ, Leibniz Universität Hannover
Schneiderberg 38, 30167 Hannover, Germany
E-mail: russell.cox@oci.uni-hannover.de

[b] E. Charria-Girón, S. Heitkemper, Prof. M. Stadler, Dr. F. Surup
Department Microbial Drugs
Helmholtz Centre for Infection Research (HZI), and German Centre for Infection Research (DZIF), Partner Site Hannover-Braunschweig
Inhoffenstrasse 7, 38124 Braunschweig, Germany
E-mail: frank.surup@helmholtz-hzi.de

[c] E. Charria-Girón, Prof. M. Stadler, Dr. F. Surup
Institute of Microbiology
Technische Universität Braunschweig
Spielmannstraße 7, 38106 Braunschweig, Germany

[d] T. E. Gorelik, ORCID: 0000-0002-0911-2039
Department of Structure and Function of Proteins
Helmholtz Centre for Infection Research (HZI)
Inhoffenstraße 7, Braunschweig, 38124, Germany1

[e] T. E. Gorelik, ORCID: 0000-0002-0911-2039
Ernst Ruska-Centre for Microscopy and Spectroscopy with Electrons (ER-C)
Forschungszentrum Jülich, 52425 Jülich, Germany

[f] C. Kleeberg, ORCID: 0000-0002-6717-4086
Institute for Inorganic and Analytical Chemistry
Technische Universität Braunschweig
Hagenring 30, 38106 Braunschweig, Germany

[g] Dr. J. M. Muema
Department Compound Profiling and Screening
Helmholtz Centre for Infection Research (HZI), and German Centre for Infection Research (DZIF), Partner Site Hannover-Braunschweig
Inhoffenstrasse 7, 38124 Braunschweig, Germany

[h] Dr B. Verwaaijen and Prof. J. Kalinowski
CeBiTec, University of Bielefeld
Universitätsstraße 27, D-33615 Bielefeld

[i] Prof. Kevin D. Hyde
Institute of Excellence in Fungal Research, Mae Fah Luang University
Chiang Rai 57100, Thailand

+ These authors contributed equally

Electronic Supplementary Information (ESI)

Table of Contents

1. Fungal material 3
2. Secondary Metabolite Profiling and Purification 3
3. Spectral Data 4
4. Derivatization of Lienhwalide B **9** with 4-Bromoaniline 5
5. Crystallography analysis of Lienhwalide B **9** 5
6. Derivatization of Lienhwalide D **11** with MTPA 8
7. Availability of Sequence Data 9
8. Details of Isotopic Feeding Experiments & NMR 10
9. Gene Cloning and Expression 11
10. LCMS Analysis for Gene Expression 19
11. NMR of Isolated Compounds 20
12. LCMS Traces for *A. oryzae lwm* BGC Transformants 41
13. Cloning, Expression and Purification of LwmR4; *in vitro* Assays & Analysis 42
14. Biological Testing 45
15. References 46

1. Fungal Material

The fungus *Hypoxylon lienhwacheense* was originally collected in the Chiang Mai Province, Thailand (Mae Tang district, highway 1095, at 22 km). The culture was isolated using multiple spore isolation techniques as described by Sir et al. (2016). After spore germination, hyphae were transferred to fresh YMA (Yeast Malt Agar) plates. The axenic culture was deposited in the Mae Fah Luang Culture Collection (MFLUCC) under the code MFLUCC 14-1231 and in the fungarium of the Helmholtz Centre for Infection Research (STMA) under the code STMA 14351. The herbarium material was deposited in the same collection under the code MFLU 14-231. This fungus was first classified as a member of the genus *Hypoxylon* based on its morphological characteristics according to the protocol of Ju and Rogers,[1] and further on confirmed to be *H. lienhwacheense* based on sequence data. [2]

2. Secondary Metabolite Profiling and Purification

Fresh stromata of *H. lienhwacheense* (3.54 g) were covered with ethyl acetate and extracted in an ultrasonic bath at 40 °C for 30 minutes. The obtained organic phase was filtered and evaporated to dryness, resulting in 503 mg of crude extract. This stromatal extract was analyzed by HPLC-DAD-MS (Figure S1).

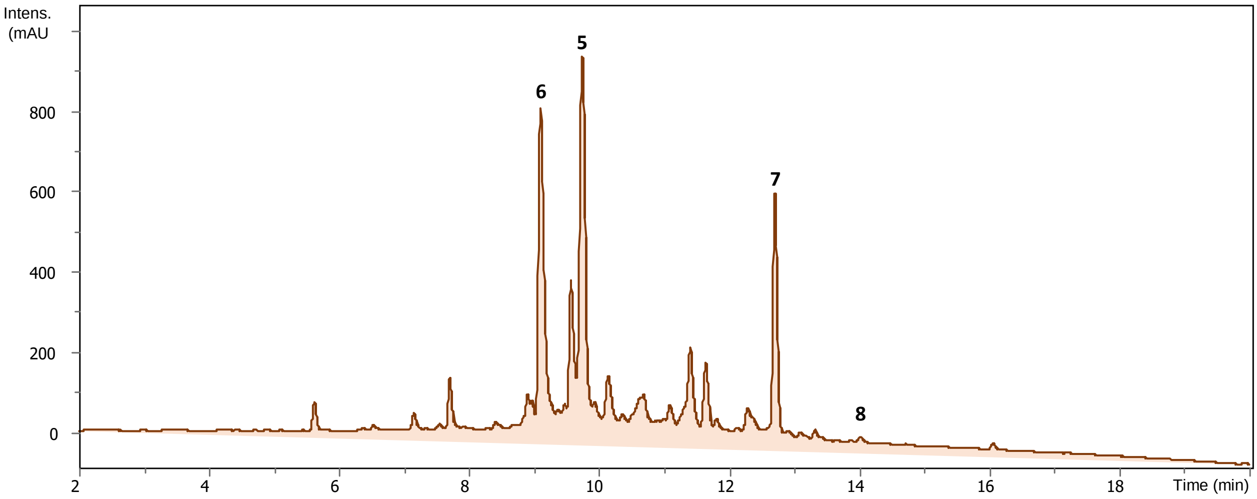

Figure S1. HPLC-UV/Vis chromatogram (210 nm) of the stromatal extract from *H. lienhwacheense* with isolated metabolites depicted in bold numbers.

Hypoxylon lienhwacheense STMA 14351 was grown on DPYA or YMA plates for 7–14 days at 28 °C. For scaled-up liquid cultivation (4 L), the fungus was cultured in 200 mL YM 6.3 medium within 500 mL shake flasks for 13 days at 25 °C under dark conditions with shaking at 150 rpm. For extraction, the cultures were separated into mycelia and supernatant by filtration, and each was extracted independently. For the supernatant, an equal volume of ethyl acetate was added, mixed in separatory funnels, and the organic phase was recovered. The extraction was repeated with the remaining aqueous phase, and afterwards the obtained organic phases were combined and evaporated to dryness. The mycelia were first covered with acetone and extracted in an ultrasonic bath at 40 °C for 30 minutes. The acetone phase was collected after filtration, and the extraction was repeated. Both acetone extracts were combined, evaporated to obtain an aqueous residue, which followed the same ethyl acetate extraction procedure as described for the supernatant. The resulting crude extracts were analyzed by HPLC-DAD-MS (Figure S2).

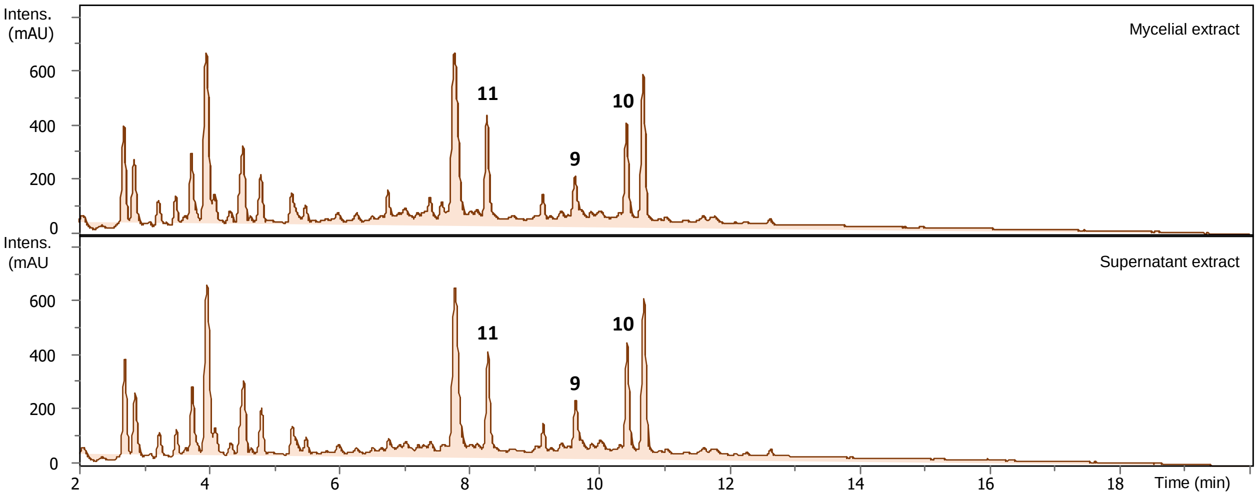

Figure S2. HPLC-UV/Vis chromatogram (210 nm) of the crude extracts obtained from the scaled-up cultivation of *H. lienhwacheense* in YM 6.3 liquid medium with isolated metabolites depicted in bold numbers.

Purification of stromatal secondary metabolites was carried out using a Gilson preparative HPLC system (Middleton, USA) equipped with a Nucleodur C_{18} ec column (125 × 40 mm, 7 µm; Macherey-Nagel, Düren, Germany). The mobile phase consisted of deionized water (A) and acetonitrile (B), both supplemented with 0.1 % formic acid (FA). The flow rate was set to 30 mL/min. The gradient was set up as follows: 30% to 80% B over 35 minutes, followed by an increase to 100% B over 5 minutes. Fractions were collected based on UV absorption and analyzed by HPLC-DAD-MS. Similar purification strategy was used for the crude extracts obtained from the scaled-up cultivation in YM 6.3 medium.

3. Spectral Data

Optical rotations were determined using an MCP 150 polarimeter at 20 °C (Anton-Paar Opto Tec GmbH, Seelze, Germany). UV/vis spectra were obtained using a UV–vis spectrophotometer UV-2450 (Shimadzu, Kyoto, Japan). HR-ESI-MS data were acquired with an Agilent 1200 Infinity Series HPLC-UV system (Agilent Technologies, Santa Clara, CA, USA) using a C_{18} Acquity UPLC BEH column [2.1 × 50 mm; 1.7 µm: Waters, Milford, MA, USA; the mobile phase consisted of deionized water (A) and acetonitrile (B), both supplemented with 0.1 % formic acid (FA), gradient starting at 5% B for 0.5 min, then increasing to 100% B in 19.5 min, and finally holding 100% B for 5 min, flow rate of 0.6 mL/min, UV/Vis detection (190–600 nm) connected to a time-of-flight mass spectrometer (ESI-TOF-MS, maXis, Bruker, Billerica, MA, USA; scan range 100–2500 *m/z*, rate 2 Hz, capillary voltage 4500 V, dry temperature 200 °C). Full NMR spectral data can be found in section 11.

Lienhwalide A **5**: Colorless amorphous powder; $[\alpha]_D^{20}$ +444.4 (c 0.003, $CDCl_3$); UV (ACN) λ_{max} (log ε) 320.8 (0.65), 249.8 (1.28), 210.8 (0.90); high-resolution electrospray ionization mass spectrometry (HR-ESI-MS): *m/z* 373.1281 $[M + H]^+$ (calculated for $C_{20}H_{21}O_7$, 373.1282).

Cordyanhidryde C **6**: Colorless amorphous powder; $[\alpha]_D^{20}$ +18.8 (c 0.0005, $CDCl_3$); UV (ACN) λ_{max} (log ε) 320.8 (0.65), 249.8 (1.28), 210.8 (0.90); ^{1}H NMR data (700 MHz, $CHCl_3$-*d*): δ_H 7.30 (dt, *J* = 15.8, 6.7 Hz, H–22), 6.20 (dt, *J* = 15.8, 1.7 Hz, H–21), 2.76 (m, H_2–2), 2.73 (m, H_2–3), 2.73 – 2.30 (m), 2.29 – 2.18 (m, H–7, H–12, H–17), 1.38 – 1.22 (m), 1.11 (t, *J* = 7.4 Hz, H–24), 0.99 – 0.95 (H_3–34, H_3–35, H_3–36) ppm; ^{13}C NMR data (175 MHz, $CHCl_3$-*d*): δ_C 173.6 (C–1), 166.6, 165.76, 165.73, 165.56, 165.54, 165.4, 165.1 (C–25), 163.8 (C–32), 151.0 (C–22), 144.5, 144.4, 144.14, 144.10, 144.08, 143.6, 138.6 (C–20), 136.4 (C–19), 115.8 (C–21), 38.14, 38.12, 37.5, 30.2 (C–2), 28.99, 28.98, 28.97, 28.83, 28.5, 28.1, 27.5 (C–23), 26.99, 26.95, 26.87, 19.8 (C–3), 12.4 (C–24), 10.72, 10.72, 10.71 ppm; high-resolution electrospray ionization mass spectrometry (HR-ESI-MS): *m/z* 723.2646 $[M + H]^+$ (calculated for $C_{38}H_{43}O_{14}$, 723.2653).

Lienhwalide B **9**: Colorless amorphous powder; $[\alpha]_D^{20}$ +250.5 (c 0.003, $CDCl_3$); UV (ACN) λ_{max} (log ε) 320.8 (0.65), 267.4 (1.25), 210.8 (0.90); high-resolution electrospray ionization mass spectrometry (HR-ESI-MS): *m/z* 403.1237 $[M + H]^+$ (calculated for $C_{21}H_{23}O_8$, 403.1387).

Lienhwalide C **10**: Colorless amorphous powder; $[\alpha]_D^{20}$ +288.8 (c 0.001, $CDCl_3$); UV (ACN) λ_{max} (log ε) 318.0 (0.40), 249.8 (1.28), 208.4 (0.67); high-resolution electrospray ionization mass spectrometry (HR-ESI-MS): *m/z* 375.1309 $[M + H]^+$ (calculated for $C_{20}H_{23}O_7$, 375.1438).

Lienhwalide D **11**: Colorless amorphous powder; $[\alpha]_D^{20}$ +324.5 (c 0.001, $CDCl_3$); UV (ACN) λ_{max} (log ε) 320.8 (0.65), 249.8 (1.28), 210.8 (0.90); high-resolution electrospray ionization mass spectrometry (HR-ESI-MS): *m/z* 413.1062 $[M + Na]^+$ (calculated for $C_{20}H_{22}NaO_8$, 413.1207).

4. Derivatization of Lienhwalide B 9 with 4-Bromoaniline

25 mg of lienhwalide B **9** were transferred to a 5 mL brown glass vial and dried under a nitrogen stream at 40 °C. After complete drying, 102 mg (9.7 equivalents) of 4-bromoaniline were added, and the mixture was stirred at 75 °C for 16 hours. The reaction product was dissolved in ethyl acetate (EtOAc) and washed with water, afterwards the organic phase was collected, dried under vacuum at 40 °C, and analyzed by HPLC-DAD-MS. The crude reaction product was purified using reverse-phase HPLC (Büchi, Pure C-850, 2020, Switzerland) with an X-Bridge C_{18} column (250 mm × 19 mm, 5 µm, Waters, Milford, MA) as the stationary phase and the mobile phase consisted of deionized water (A) and acetonitrile (B), both supplemented with 0.1 % formic acid (FA). The flow rate was set to 20 mL/min. The gradient was set up as follows: 5% to 45% B over 5 minutes, then from 45 % to 70 % B in 40 min, followed by an increase to 100% B over 10 minutes. Fractions were collected based on UV absorption and analyzed by HPLC-DAD-MS.

Compound **9a**: Yellow to colorless amorphous powder; ^{1}H NMR data (500 MHz, $CHCl_3$-*d*): δ_H 7.55 (br d, *J* = 8.8 Hz, H–3'/H–5'), 7.24 (dt, *J* = 15.9, 6.7 Hz, H–18), 7.19 (br d, *J* = 8.8 Hz, H–2'/H–6'), 6.78 (br s, H–5), 6.43 (dt, *J* = 15.9, 1.6 Hz, H–17), 5.61 (dd, *J* = 7.4, 3.7 Hz, H–11), 4.03 (a, H_3–6OMe), 3.70 (dqd, *J* = 10.8, 6.1, 1.6 Hz, H–2), 3.20 (dd, *J* = 13.3,3.8 Hz, H–12a), 3.01 (dd, *J* = 13.3, 7.4 Hz, H–12b), 2.88 (br dd, *J* = 16.1, 10.8 Hz, H–3a), 2.65 (br dd, *J* = 16.1, 1.6 Hz, H–3b), 2.27 (m, H_2–19), 1.29 (d, *J* = 6.1 Hz, H_3–1), 1.11 (t, *J* = 7.4 Hz, H_3–20) ppm; ^{13}C NMR data (125 MHz, $CHCl_3$-*d*): δ_C 170.1, 169.4, 160.5, 160.1, 153.1, 147.8, 146.5, 138.9, 136.0, 134.1, 132.0, 131.0, 127.8, 127.2, 120.9, 117.9, 115.6, 73.4, 68.7, 57.0, 42.6, 29.0, 27.5, 21.2, 12.7 ppm; high-resolution electrospray ionization mass spectrometry (HR-ESI-MS): *m/z* 556.0991 $[M + H]^+$ (calculated for $C_{27}H_{27}BrNO_7$, 556.0971).

5. Crystallography Analysis of Compound 9a

Crystals of **9a** precipitated from an acetone/water solution. The crystal structure was initially determined using synchrotron X-ray diffraction data. For data collection, a few crystals were cryo-protected with 10% (*v/v*) (*R,R*)-2,3-butane-diol and flash-cooled in liquid nitrogen. Data collection was carried out at 100 K on beamline P11 of the PETRA III storage ring at the Deutsches Elektronen-Synchrotron (DESY, Hamburg, Germany). The HDF diffraction files were converted to CBF data format using the *eiger2cbf* converter. The CBF files were then processed in CrysalisPro. All analyzed crystals appeared severely twinned, so although the crystal structure could be determined, the refinement was not conclusive and no absolute configuration could be determined.

We then turned to electron diffraction, hoping that small crystals would not demonstrate as much twinning. For a known structure, the requirements for absolute structure determination through dynamical refinement are not very restrictive.[3] We collected 3D ED data in a GLACIOS TEM at liquid nitrogen temperature. Indeed, the nanocrystals were single crystalline. Yet, the crystal quality was low, with the data resolution being in the range of 2 Å. The data were processed in PETS2 and dynamical refinement was performed in JANA.[4,5] However, the data quality did not allow us to draw conclusions about the handiness of the structure.

We finally returned to X-ray diffraction and selected a few crystals for in-house single crystal X-ray data analysis. After scanning several crystals, we managed to select one with no twinning and determined the crystal structure together with the absolute configuration. The relevant crystallographic data are presented in Table S1. The crystals undergo a reversible phase transition between 220 K and 180 K with symmetry reduction. The higher temperature phase is orthorhombic $P2_12_12$ (No. 18, Z = 8, Z' = 2,), whereas at the lower temperatures a monoclinic phase is observed (in $P2_1$ with Z = 8, Z' = 4, β = 92.4°) and twinning *via* two-fold axis parallel c. A refinement of the structure of the low-temperature form was not successful. At 220 K, a complete data set was obtained, which was reliably refined. The phase transition upon cooling explains why all our previous attempts to refine the crystal structure were unsuccessful – the synchrotron and electron diffraction data were collected at liquid nitrogen temperature – from the poorly crystalline low-temperature polymorph. The crystal structure of

high-temperature polymorph of **9a** (Figure S3) contains two independent molecules in the asymmetric unit (Z' = 2), which differ considerably in their conformations. These two independent molecules form an asymmetric dimer *via* H-bridges (Figure S4).

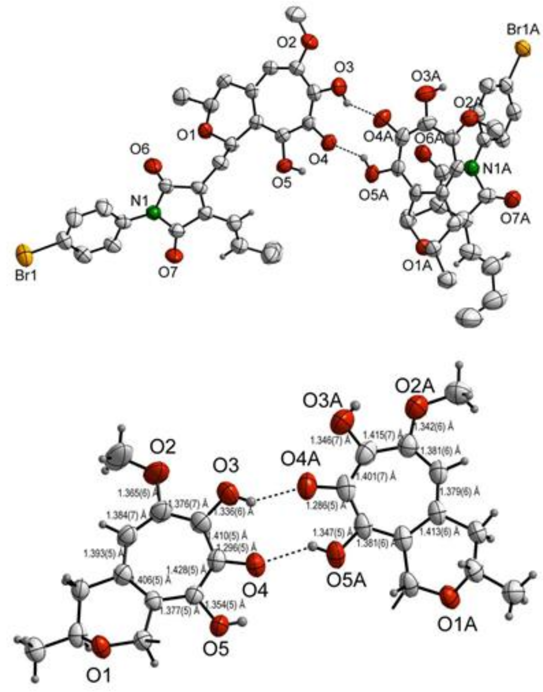

Figure S3. Crystal structure of the high-temperature polymorph of **9a** – two symmetry independent molecules (top) and the close up of selectedintermolecular interactions (bottom).

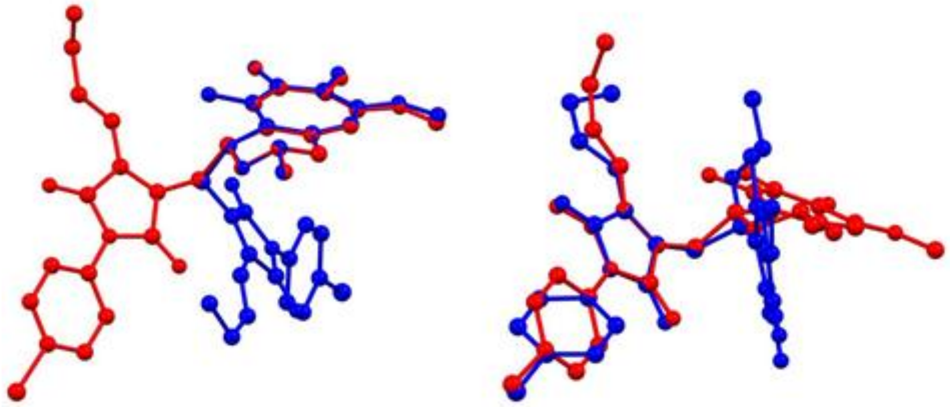

Figure S4. Overlay of the two independent molecules in the high-temperature polymorph of **9a**. Fitted atoms: O1/O1A, C2/C2A, C11/C11A, (left) and N1/N1A, C13/C13A, C16/C16A, (right).

The 7-rings are planar with only slight deviations from planarity (deviation from the best plane: C10 0.079(4) Å, C10A 0.066(4) Å). The C-C distances also suggest an essentially conjugated 7-ring. In line with this, C-O distances between the values for a single and double bond are found for the exo-cyclic oxygen atoms. The distance to C-O4/O4A is slightly smaller than the average and can be interpreted as a double bond, i.e., C=O. However, this does not rule out the (dynamic) presence of other tautomers. It is also conceivable that oxygen atoms are not exclusively present as substituents on the 7-ring. After refinement of the two independent molecules, residual electron density remains in the structure, which could not be meaningfully described and was taken into account using the BYPASS algorithm (see deposited data for details). Nevertheless, a reliable assignment of the absolute stereochemistry was possible without any doubt. The two polymorphs have very similar crystal structures, with only a slight shift in the positions of the molecules (Figure S5).

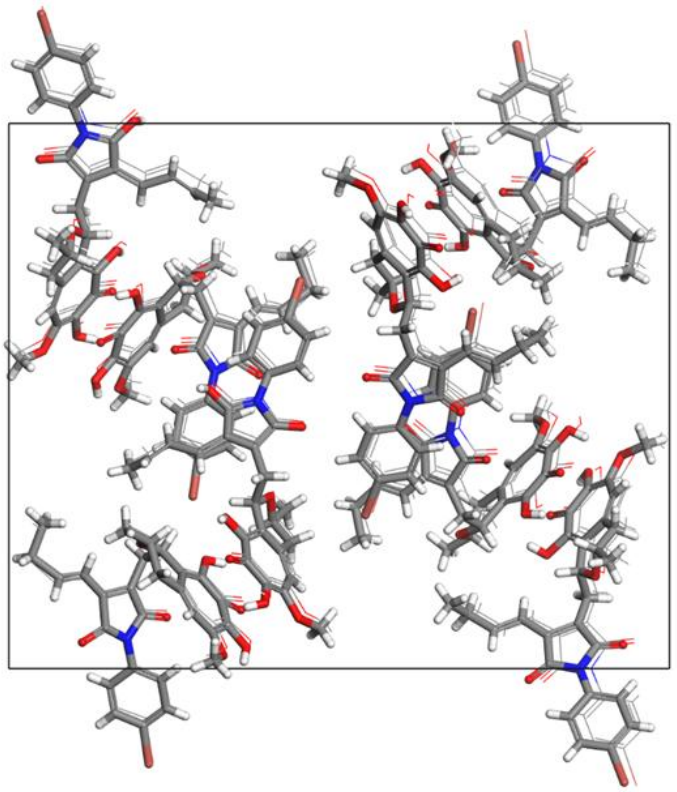

Figure S5. Overlay of the two crystal structures of **9a**: the low-temperature phase is shown in stick representation, while the high-temperature polymorph is depicted in wire representation. The view is along the c-axis of the orthorhombic phase, with the a-axis horizontal and the b-axis vertical.

Table S1. Crystal data and structure refinement for **9a**.

	Low-temperature phase[a]	High-temperature phase
**Sum formula**	$C_{27}H_{26}BrNO_7$	$C_{27}H_{26}BrNO_7$
**Moiety formula**	$C_{27}H_{26}BrNO_7$	$C_{27}H_{26}BrNO_7$
**Formula weight**	556.40	556.40
**Temperature**	100 K	220(2) K
**Wavelength**	0.61992 Å	1.54184 Å
**Instrument (scan mode)**	P11 / PETRA III, one axis scan	XtaLAB Synergy, Single source at home/near, HyPix (w scans)
**Crystal system, Space group**	Monoclinic, *P2₁* (no. 4)	Orthorhombic, *P2₁2₁2* (no. 18)
**Unit cell dimensions**	a = 7.82(4) Å<br>b = 24.288(15) Å<br>c = 29.380(19) Å<br>a= 90°<br>b= 91.63(14)°<br>g = 90°	a = 30.026(7) Å<br>b = 24.775(6) Å<br>c = 7.856(2) Å<br>a= 90°<br>b= 90°<br>g = 90°
**Volume, Z, Z'**	5580(26) $Å^3$, 8, 4	5844(3) $Å^3$, 8, 2
**Cell determination**		3943 refl., 2.936° <= q <= 70.329°
**Density (calculated)**		1.265 Mg/m^3
**Absorption coefficient**		2.265 mm^{-1}
**F(000)**		2288
**Crystal habitus**	irregular (clear colorless)	irregular (clear colorless)
**Crystal size**	0.1 x 0.1 x 0.05 mm^3	0.160 x 0.110 x 0.070 mm^3
**Data collection**		2.312° < q < 83.989°
**Completeness to q = 71.000°**		99.9 %
**Index ranges**		-36 <= h <= 37, -31 <= k <= 30, -10 <= l <= 9
**Reflections collected / indep. / obs.**	23911 / 23911 / 6577	167780 / 12130 (R_{int} = 0.0377) / 11498
**Absorption correction**		Gaussian
**Max. and min. transmission**		1.000 and 0.629
**Solution method**		iterative methods
**Refinement method**		Full-matrix least-squares on F^2
**Hydrogen solution / refinement**		geom / constr
**Data / restraints / parameters**	23911 / 1/ 1271	12130 / 0 / 659
**Goodness-of-fit on F^2**	0.77	1.029
**Final R indices [I>2s(I)]**	0.114, 0.308,	R_1 = 0.0410, wR_2 = 0.1171
**R indices (all data)**		R_1 = 0.0435, wR_2 = 0.1186
**Absolute structure parameter**		-0.012(3)
**Largest diff. peak and hole**		0.614 and -0.406 e.$Å^{-3}$
**Weighting scheme**		$w=1/[s^2 (F_o)^2+(0.0721P)^2+2.0163P]$ where $P=(F_o^2+2F_c^2)/3$
**CSD deposition code**	2388480	2388253

[a] The crystal was twinned. No convincing refinement of the structure was possible.

6. Derivatization of Lienhwalide D (11) with MTPA

Lienhwalide D (**11**) was dissolved in pyridine-d_5 (50 µL) and transferred into a 250 µL glass vial, and (R)-(−)-α-methoxy-α-(trifluoromethyl) phenylacetyl chloride (4 µL) was added. The mixture was incubated for 16 h at 40 °C before being transferred to an NMR tube (600 µL) and diluted with pyridine-d_5 to a final volume of 350 µL for the measurement of NMR spectra. Similarly, the (R)-MTPA ester derivative was obtained analogously with (S)-(+)-α-methoxy-α-(trifluoromethyl) phenylacetyl chloride (4 µL). The NMR spectral data for the MTPA-esters of **11** are found in section 11.

7. Availability of Sequence Data

The nucleotide sequences of the BGCs are available at GenBank under the accession numbers PQ671038 (*lwm* BGC) and PQ671039 (*lwt* BGC). An overview of the content of both BGCs is summarized in Table S2.

Table S2 Gene cluster analysis for the *lwm* and *lwt* BGCs.

Gene	Length [bp] / [aa]	Putative function	blastx hit, identity	Identity dba-BGC protein
lwtS	7833 / 2611	nr-PKS	Type I Iterative PKS, *Coccidioides posadasii str. Silveir,* 65 %	62 %
lwtR1	1389 / 463	FAD-dependent monooxygenase	FAD/NAD(P)-binding domain-containing protein, *Hypoxylon sp. FL0890*, 78 %	62 %
lwtR2	1395 / 465	FAD-dependent oxidoreductase	FAD/NAD(P)-binding domain-containing protein, *Hypoxylon fuscum*, 76 %	48 %
lwtR3	1623 / 541	transcription factor	fungal-specific transcription factor domain-containing protein, *Hypoxylon rubiginosum*, 77 %	45 %
lwtR4	1023 / 341	enoyl-reductase-like enzyme	oxidoreductase, *Daldinia loculata*, 82 %	58 %
lwtR5	1416 / 472	transporter	MFS general substrate transporter *Hypoxylon trugodes*, 82 %	58 %
lwtR6	330 / 110	YCII-domain containing protein	YCII-related domain-containing protein, *Hypoxylon sp. EC38*, 78 %	47 % (each)
lwtR7	1002 / 334	non-heme iron-dependent (NHI) dioxygenase	Clavaminate synthase-like protein, *Hypoxylon sp. NC0597*, 90 %	69 %
**Gene**	**Length [bp] / [aa]**	**Putative function**	**blastx hit, identity**	**Identity bf-BGC protein**
lwmA	7830 / 2610	hr-PKS	Type I Iterative PKS, *M. purpureus*, 54 %	47 %
lwmR1	666 / 222	hydrolase	esterase alnB, *A. nidulans,* 40 %	54 %
lwmR2	684 / 228	MDC	ketosteroid isomerase-like protein - *Paecilomyces fulvus,* 39 %	39 % (BfL6) 37 % (BfL10)
lwmR3	1482 / 494	alkylcitrate dehydratase	2-methylcitrate dehydratase-like OryR, *A. oryzae*, 51 %	65 %
lwmR4	1326 / 442	alkylcitrate synthase	citrate synthase OryE, *A. oryzae*, 43 %	50 %
lwmR5	1215 / 405	unknown	-	-
lwmR6	1758 / 586	acyl-CoA ligase	acyl-CoA ligase EasD, *A. nidulans*, 41 %	-

8. Details of isotopic feeding experiments

Feeding experiments with 13C-labelled precursors (^{13}C-acetate, ^{13}C-methionine, 1-^{13}C- and *U*-$^{13}C_6$-glucose) were conducted as in Surup et al.[6] Enrichment of lienhwalide B (**9**) was analyzed by ^{13}C NMR spectroscopy (Table S3).

Table S3 ^{13}C NMR Data of lienhwalide B (**9**) from feeding experiments.

**Pos**	**δ_C, mult.**	**[1-^{13}C]-methionine[a]**	**[1-^{13}C]-glucose[b]**	**[*u*-$^{13}C_6$]-glucose[c]**	**[1-^{13}C]-glucose[d]**
1	20.9, CH_3	0.0	0.7	41	2
2	68.6, CH	-0.1	-0.5	41	1
3	42.4, CH_2	-0.1	0.6	42	4
4	139.0, C	-0.6	-0.8	42	3
5	114.9, CH	-0.1	0.5	72	6
6	153.3, C	-1.0	-0.8	72	5
7	147.1, C	-0.9	-0.4	17	9
8	159.2, C	27.2	-0.6		
9	161.2, C	-0.9	-0.8	17	7
10	127.5, C	-0.7	-0.4	44	11
11	73.5, CH	-0.4	-0.4	44	10
12	29.1, CH_2	-0.1	0.4		13
13	136.0, C	-0.7	-0.4	62	12
14	165.8, C	-0.8	-0.8	62	(13)
15	164.6, C	-0.9	-0.9	60	16
16	138.7, C	-0.7	-0.3	60	15
17	117.0, CH	-0.1	-0.5	68	18
18	149.0, CH	-0.2	1.0	68	17
19	27.3, CH_2	0.1	-0.2	34	20
20	12.4, CH_3	0.0	1.4	34	19
6-OMe	56.9, CH_3	84.9	0.0		

[a] ^{13}C specific incorporation referenced to atom: C-1. [b] ^{13}C specific incorporation referenced to atom: C-6-OMe. Significant specific incorporations are indicated by shading. [c] coupling constants (in Hz) extracted from ^{13}C NMR spectrum. [d] Couplings to signals in INADEQUATE NMR spectrum

9. Gene Cloning and Expression

9.1 Construction of Vectors

Hypoxylon lienhwacheense was grown in YMG liquid medium for 4 days. Mycelia was collected and used for total RNA preparation (RNA Clean and Concentrator™ Kit, *Zymo Research*) following manufacturers instructions. The RNA was reverse transcribed to cDNA using an oligo-dT primer (High Capacity RNA-to-cDNA Kit, *Thermo Fisher Scientific*) following manufacturers instructions to give intron-free sequence. The obtained cDNA was used as the template for all subsequent cloning procedures. Vectors for heterologous expression were constructed by yeast recombination and LR-recombination methods, as described in Pahirulzaman et al.[7] The template for PCR amplification (Figure S6 A and D) was *H. lienhwacheense* cDNA with appropriate primer pairs (H1/H2, 2MDH1/2MDH2, CS1/CS2, Table S4). The primer included overhangs of 30 bp homologous to regions to the sequence of the insertion site of the vector to result in appropriate fragments for yeast recombination (Figure S6 A, D). The genes *lwmR1*, *lwmR3* and *lwmR*4 were inserted into the fungal expression vector pTYGS·*ade* after restriction digestions with the enzyme *Asc*I (Figure S6 E).

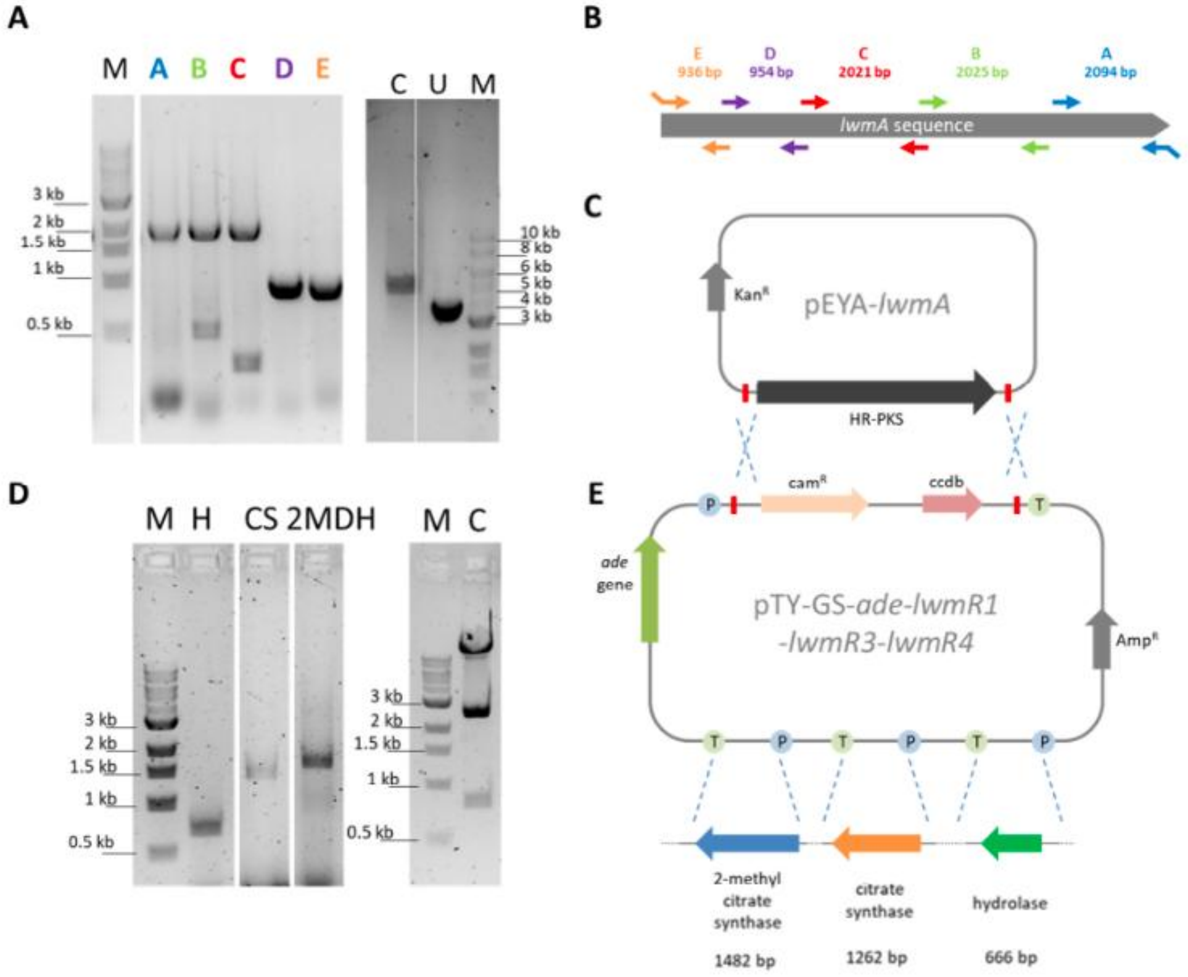

Figure S6 Vector construction of fungal expression vector including four putative genes for maleic anhydride biosynthesis: **A**, fragments used for pEYA·*lwmA* construction (C = cut vector, U = uncut vector); **B**, scheme of *lwmA* fragments for yeast recombination; **C**, resulting pEYA·*lwmA* vector after yeast recombination; **D**, fragments used for construction of the expression vector (C = cut vector); E, resulting pTYGS·*ade·lwmR1·lwmR3·lwmR4* vector.

The putative PKS-gene *lwmA* was first reconstructed in the entry vector pEYA. The vector was previously digested using the restriction enzymes *Not*I and *Asc*I. Due to the length of approx. 7800 bp, yeast recombination was performed using five overlapping fragments (Figure S9.1.1 A-B, fragments A-E) of the PKS sequence using primers pairs A1-E2 (Table S9.1). After recombination, the expected resulting vector (Figure S9.1.1 C) was verified with PCR amplification and following sequencing methods as described previously. Subsequent performance of *in vitro* LR recombination of vectors pEYA·*lwmA* and pTYGS·a*de·lwmR1·lwmR3·lwmR4* completed the construction of the final expression vector pTY·*ade·lwmA ·lwmR1·lwmR3·lwmR*4.

Table S4 Summary of used oligonucleotides for lienhwalide BGC expression experiments.

Description	Name	Sequence
lwmA	A1	TGCCAACTTTGTACAAGAAAGCTGGGTCGGCTAGTCCCCCTTTTCTGCCT
	A2	GAAGGTTTCATCAGCATCGAGGTC
	B1	GTCTTTGCGGGTGAGGCCTGA
	B2	CCATGTTCGTTGCGCTGGGGT
	C1	CAGCTTTGCGCCTGACCCCA
	C2	AAGCCAAACCGATACGTACCC
	D1	GGACTGGCTTCTTCTTAAGGG
	D2	TGGATAGTGTCATGGTCTCTCAAGG
	E2	GCCAACTTTGTACAAAAAAGCAGGCTCCGCATGACACCAATTTCCCTCGAAG
	E1	TGGATAGTGTCATGGTCTCTCAAGG
	LWPKS_FR1_rev	CACGAACCGAGAATGGAGCGTT
	LWPKS_FR2_fw+30	CTAGCCTGAACGCTCCATTCTCGGTTCGTGAGGAAATCACGCGGAACCCTG
	LWPKS_FR3_rev	CCTAAGAACCATCGCTGCTTGGA
	LWPKS_FR4_fw+30	GGAGTTATCCAAGCAGCGATGGTTCTTAGGGATAACTATATCGAGAAGATGACCGTGG
lwmR1	H1	TTCTATGCGTTATGAACATGTTCCCTGGCGCTACCATACATGCAAAACTTCCAT
	H2	TTTCAACACAAGATCCCAAAGTCAAAGGCGATGCCTTTAAGATTACTTTGCCTTC
lwmR3	2MDH1	CAATGTCCATATCATCAATCATGACCGGCGTCATAGCTTCGGACCAGAGCC
	2MDH2	GCTACCCCGCTTGAGCAGACATCACCGGCGATGACTCCTACCCCTAAATCTAGC
lwmR4	CS1	GGCTGGTAGACGTCATATAATCATACGGCGCTACAGCTTTGACGAGACATCCCTG
	CS2	ACTGACCAATTCCGCAGCTCGTCAAAGGCGATGTCGGAAGGGACTCTTCACGTG
	CS_pET28_NdeI_fw	CGGCAGCCATATGATGAGCGAAGGCACCCTGCA
	CS_pET28_NotI_re	GCTCGAGTGCGGCCGCTTACAGTTTGCTGCTAACATCAC
lwmR2	KI1	ACTTTAAGAAGGAGCCCTTCACCAAGGGTGATGAAGATCTCTTTTACGATTCCCGC
	KI2	AATGCCAACTTTGTACAAGAAAGCTGGGTCTTAAGGAGCAAGAGAATTGGGATCG
	KI_exSP_fw	GCCAACTTTGTACAAAAAAGCAGGCTCCGCATGGCGCATCGGGGATATGCCAAC
	KI-fw-pET28a	CATATGGCTAGCATGGCGCATCGGGGATATGC
	KI-rev-pET28a	GTGGTGCTCGAGTTAAGGAGCAAGAGAATTGGGA
lwmR6	ACL1	TTCTTTCAACACAAGATCCCAAAGTCAAAG ATGGTCTTCGTATCTTTCCCTAAAG
	ACL2	TTTCATTCTATGCGTTATGAACATGTTCCC CTAAAGTCTCGCTGCTGTGGC
lwmR5	R5gpdA_fw	TGACCCACTGGGGTTTTAGGAGGTCAATTG ATGCTAGAGTCTTTCTTCGGT
	R5eno_rev	CAGGTTGGCTGGTAGACGTCATATAATCATACG CTAACTATAATTAATGTTACTT
	Lwt30R (pET28a-LwtR1)	ATCTCAGTGGTGGTGGTGGTGGTGCTCGAGTCATTCATATGCGACAGCACCGTTC
	Lwt8F	TCTTTCAACACAAGATCCCAAAGTCAAAGGATGCCTGAAATGATCAATGA
	Lwt8R	CTATGCGTTATGAACATGTTCCCTGGCGCGTCATTCATATGCGACAGCAC

9.2 Transformation of Core Genes

The four core genes *lwmA* (PKS), *lwmR1* (hydrolase), *lwmR4* (alkylcitrate synthase), *lwmR3* (2-methycitrate dehydratase of the proposed pathway were all shown to be present in the vector pTY·*ade*·*lwmA*·*lwmR1*·*lwmR3*·*lwmR4* by PCR. This vector was transformed into the heterologous host *A. oryzae* NSAR1. The transformants were selected on media lacking adenine, cultivated in liquid media extracted and the residues analysed by LCMS. Overall, 16 transformants were obtained. Four transformants showed production of maleic acid monomer **19a** (Figure S7), characterized by a new peak at 6 min with the typical UV maximum of 314 nm and a characteristic *m/z* of 165 ([M - CO_2]) in ES spectra. These characteristic data and the retention time indicate the maleic acid anhydride compound previosly identified by our group.[8]

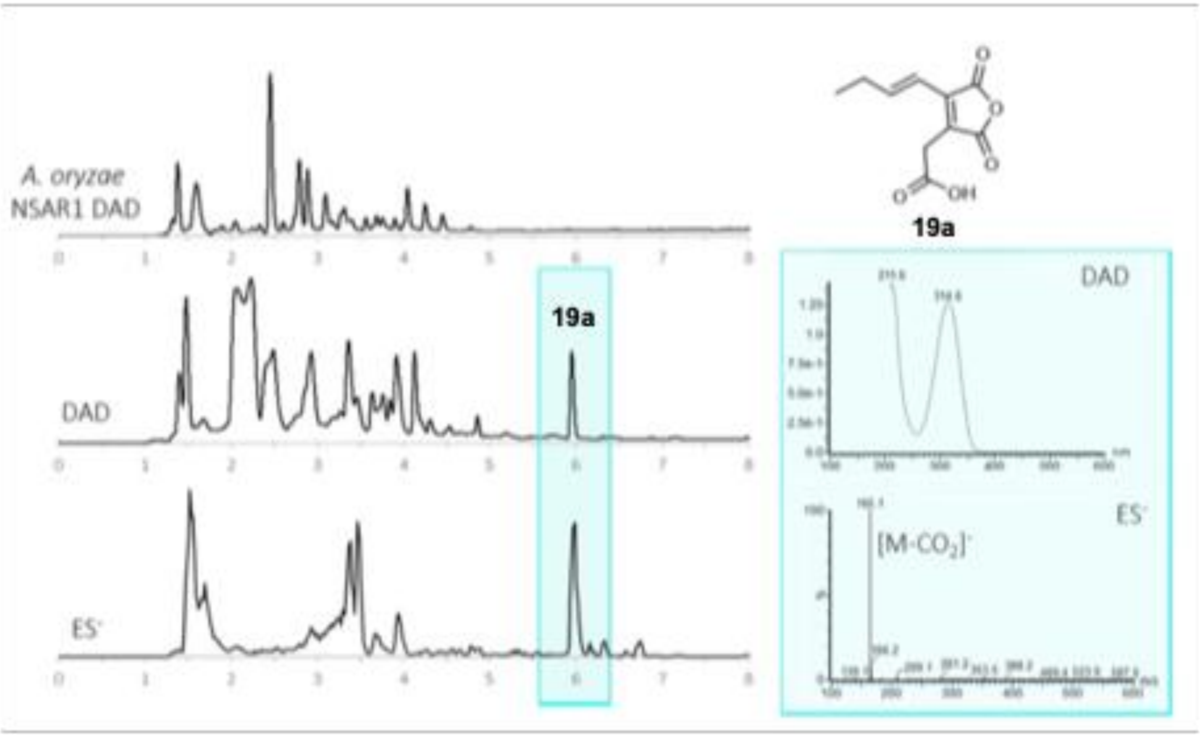

Figure S7 LCMS analysis of transfomant including *lwmA, lwmR1, lwmR3*, *lwmR4*: DAD chromatograms (210 – 600 nm) of WT and transformant, ES^- and ES^+ chromatograms of transformant and corresponding spectra at 6 min.

9.3 Co-expression of LwmR6 with LwmA + R1 + R3 + R4

The putative ligase encoded by *lwmR6* was added to the heterologous expression system with the vector pTYGS·*argB*·*lwmR6* (constructed with previously described methods with primers ACL1/ACL2), which was simultaneously transformed with the previously obtained vector pTY·*ade*·*lwmA*·*lwmR1*·*lwmR3*·*lwmR4* followed by selection on media lacking arginine and adenine, cultivation and LCMS analysis. Thirteen out of seventeen of the selected transformants produced maleic acid monomers **19a** (Figure S8) in high amounts.

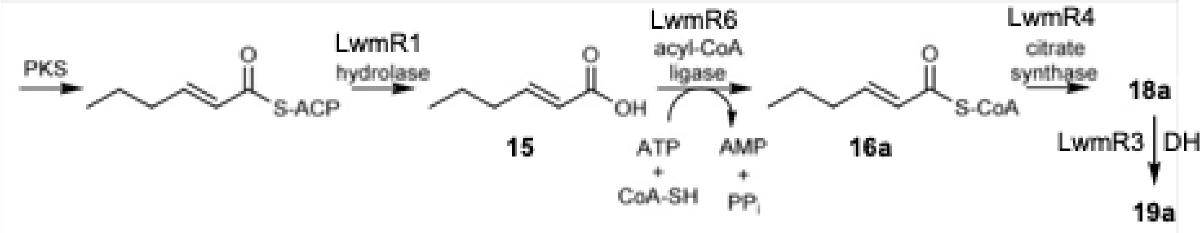

Figure S8 Reaction of Acyl-CoA ligase LwmR6.

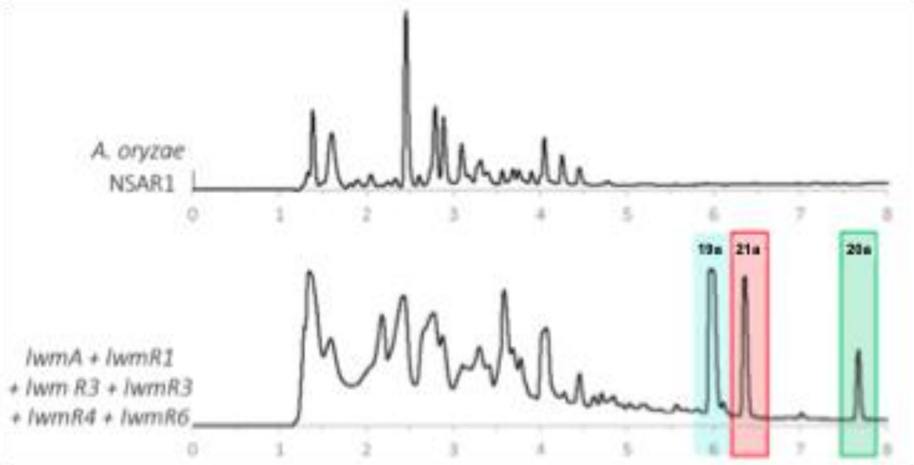

Figure S9 DAD chromatogram (200–600 nm) of representative *A. oryzae* transformant *lwmA·R1·R3·R4·R6*.

In comparison to the previous expression of transformations including the core 4-gene set of enzymes for the pathway, two additional peaks were observed (Figure S9, red and green). The peak at 7.5 min (green) was identified as **20a**, previously identified by us as the decarboxylation product of **19a**.[7]

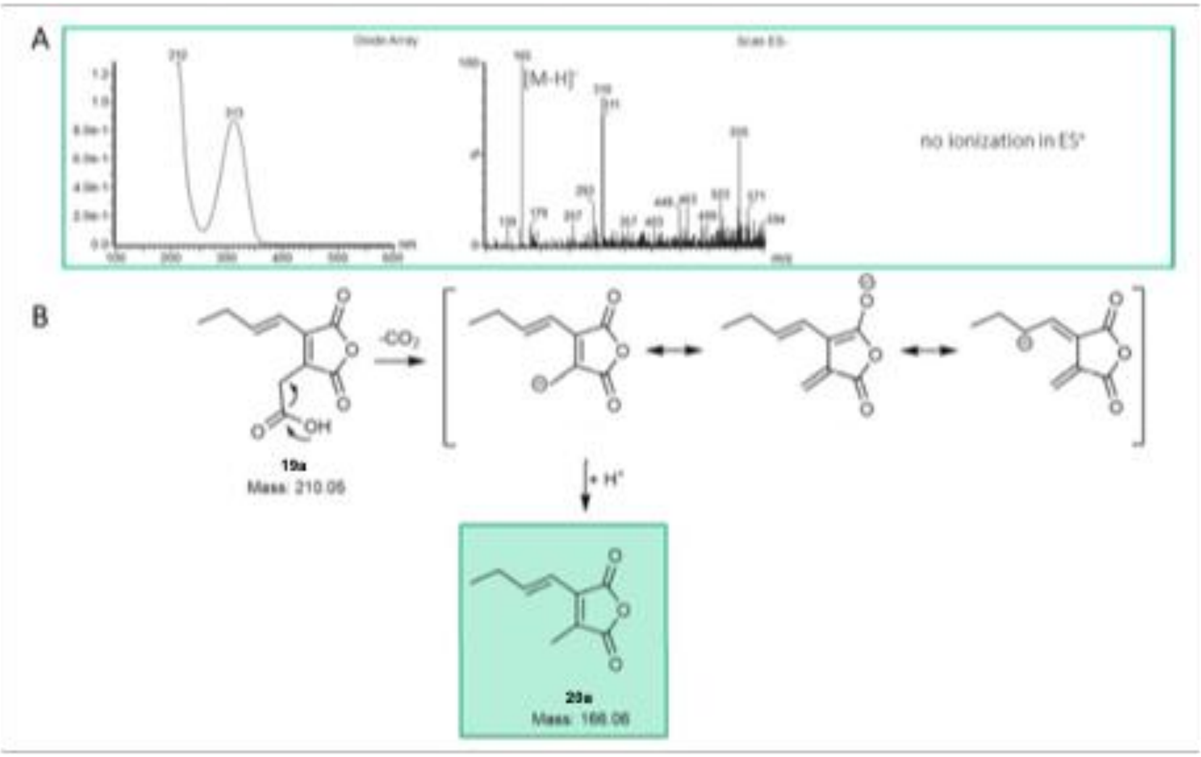

Figure S10 Characterisation of the peak at 7.7 min: **A**, LCMS spectra; **B**, proposed mechanism.

Twelve of the transformants produced new compound **21a** at 6.5 min. The characteristic UV at 311 nm (Figure S10) indicated a relationship to the maleic acid anhydrides **19a** and **21a**. The mass was speculated to be 224 Da by ES^+/ES^- spectra with peaks at a *m/z* of 447 ($[2M - H]^-$), 179 ($[M - CO_2 - H]^-$) and 225 ($[M + H]^+$).

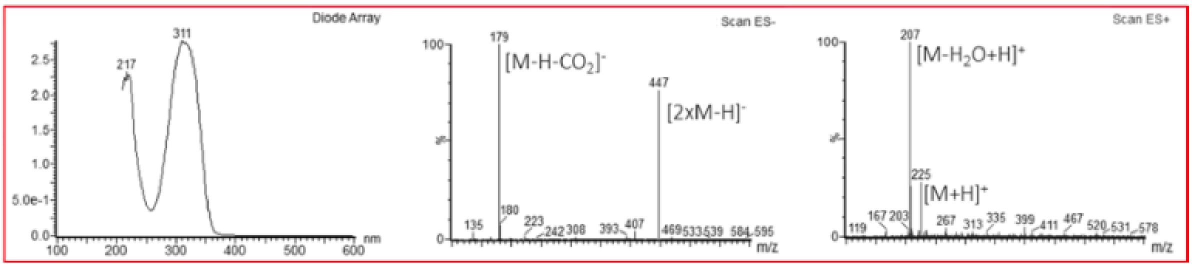

Figure S11 Characterisation of the peak **21a** at 6.5 min.

For the identification of compound **21a** the transformant was cultivated at higher scale (1.9 litre) and **21a** was isolated by preparative LCMS. Overall, approx. 1.4 mg/L were obtained. The structure of **21a** was elucidated by full NMR analysis (Section 11). A full overview of the expression analysis of the *lwm* BGC can be found in Figure S12 and in the Scheme S1.

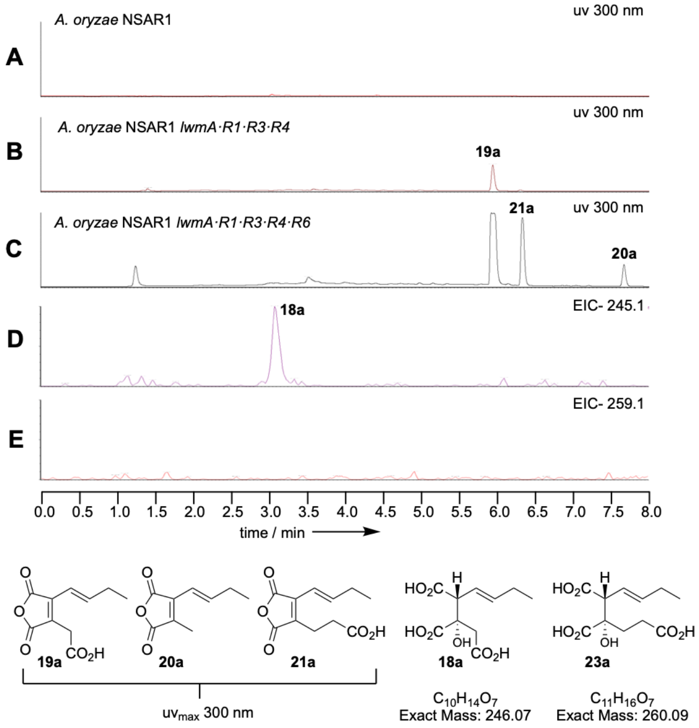

Figure S12. LCMS analysis of expression of the *lwm* BGC in *A. oryzae*. **A**, Analysis (uv 300 nm) of untransformed *A. oryzae* NSAR1; **B**, Analysis (uv 300 nm) of *A. oryzae* NSAR1 *lwmA·R1·R3·R4*; **C**, Analysis (uv 300 nm) of *A. oryzae* NSAR1 *lwmA·R1·R3·R4·R6*; **D**, LCMS analysis (extracted ion chromatogram, EIC) of *in vitro* reaction **16a** and **17** catalysed by LwmR4; **E**, LCMS analysis (extracted ion chromatogram, EIC) of *in vitro* reaction of **16a** and **22** catalysed by LwmR4.

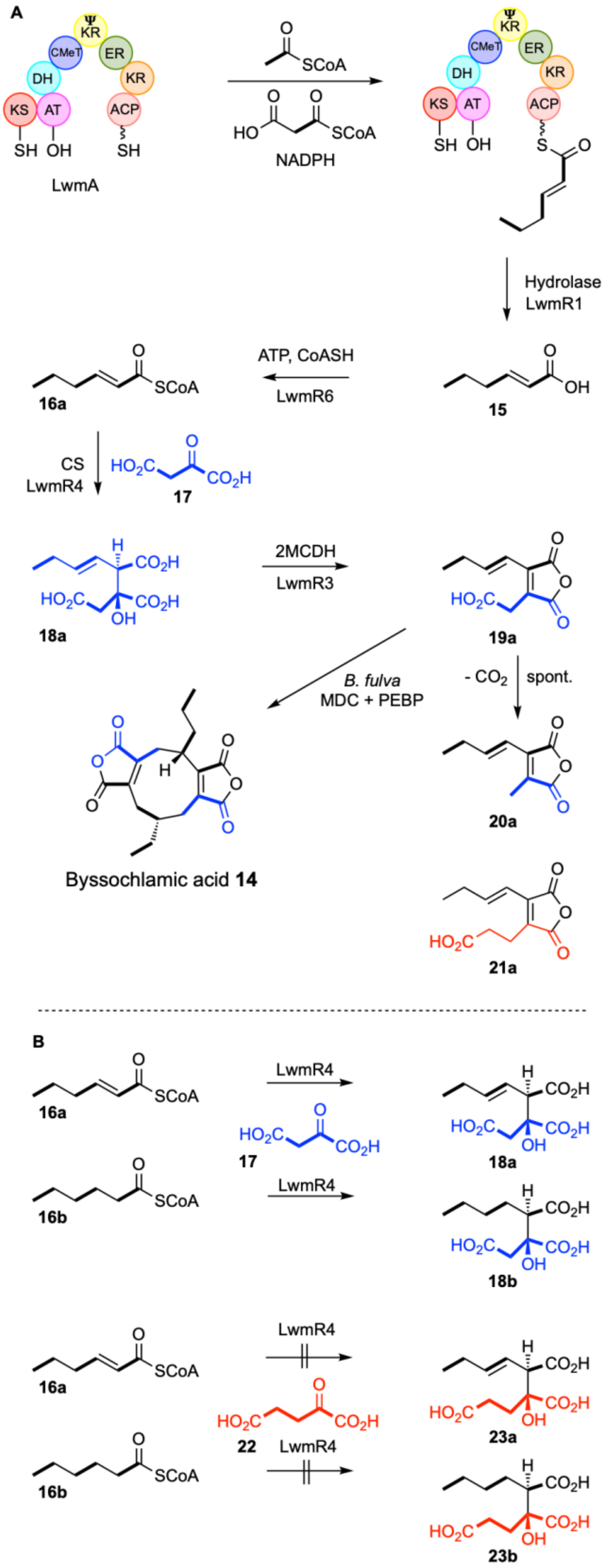

Scheme S1. **A**, Results of heterologous expression of genes *lwmA·R1·R6·R4·R3* in *Aspergillus oryzae*; **B**, *in vitro* analysis of LwmR4.

9.4 Growth and Transformation of Bacterial, Yeast and Fungal Strains

9.4.1 Heat-shock Transformation of *E. coli* Strains

Escherichia coli was grown on LB-agar or in liquid LB-medium with corresponding antibiotics. The cells were cultivated at 37 °C for approx. 12 h. If grown in liquid media, the culture was shaking at 200 rpm. For long-term storage, the liquid culture was mixed with 50 % glycerol to a final concentration of 25 % glycerol and stored at -80 °C. Competent *E. coli* strains were thawed on ice after -80 °C storage. 60–100 ng purified plasmid was added to 50 µl *E. coli* cells and placed on ice for 30 min, followed by a heat shock at 42 °C for 30 s and cooling on ice for 2 min. Finally, 250 µl SOC-medium was added to the cells and the mixture was incubated at 37 °C and 300 rpm for 1 h. The transformed cells were spread out on LB-agar plates containing appropriate antibiotics and incubated at 37 °C overnight.

9.4.2 Transformation of *S. cerevisiae* and Yeast Recombination

Saccharomyces cerevisiae was grown on solid YPAD agar at 30 °C for 3–5 days. One single colony was used for inoculation for 10 mL liquid YPAD medium. The culture was grown overnight at 30 °C and 200 rpm. After transformation of cells with vectors containing ura3 cultivation took place with selective SM-URA-Agar and at 30°C for 3–5 days. The transformation was done using the LiOAc/SS carrier DNA/PEG protocol developed by Gietz and Woods.[9,10] Therefore, a single yeast colony was picked to inoculate 10 mL YPAD medium and grown overnight at 30 °C while shaking at 200 rpm. 40 mL fresh YPAD medium was added to the pre-culture the next day and incubated under the same conditions for 4-5 h. After harvesting the cells by centrifugation (all following centrifugations: Pico™ 17 or Fresco™ 21 Microcentrifuge, Thermo Scientific) at 3000 x g for 5 min at 4 °C they were washed with 25 mL water. The cell pellet was then re-suspended with 1 mL water and transferred to a 1.5 mL tube. The mixture was centrifuged again for 30 s at 11000 x g. The supernatant was discarded. A 1 mL suspension was obtained from the pellet with water, which was divided into 100 µl aliquots. For the preparation of the transformation mixture the following ingredients (Table S5) were mixed together on ice. The cells were incubated with the transformation mix for 42 °C for 50 min. Cells were pelleted at 11000 x g for 30 s and re-suspended with 500 µl water. 250 µl of the cell mixture was spread on selective SM-URA plates and incubated for 3–5 days at 30 °C.

Table S5 Transformation mixture for yeast recombination**.**

Volume	Ingredient	Comment
50 µL	ssDNA (single-Strand Carrier DNA or salmon sperm DNA, 2 mg/mL)	prepared by boiling at 95 °C for 5 min
240 µl	PEG 3350	50 % w/v
36 µl	LiAc	1 M
34 µl	DNA in water (cut plasmid + fragments, equimolar)	up to 5 µg

9.4.3 Fungal Strains and Transformation

Aspergillus oryzae NSAR1 was grown on DPY-plates at 28 °C for 5–7 days. The mycelium was used to inoculate 50 mL GN-medium in 250 mL shake flask. The culture was incubated at 28 °C and 110 rpm for approx. 18h. The biomass was separated from the media by filtration with a miracloth filter. Mycelia was incubated with 10 mL *Trichoderma* lysing enzyme (Sigma Aldrich) solution or VinoTaste® Pro (Novozymes) solution (10 mg/mL enzyme, previously sterilised by disposable sterile filter with 0.45 µm pore size [Roth]) while shaking at room temperature and 2 rpm for 3–5 hours. After incubation, the biomass was gently pipetted up and down to release the protoplasts from hyphal strands. The protoplasts were obtained by filtration with a miracloth filter and centrifuged at 3000 x g for 5 min. The resulting pellet was resuspended with transformation solution 1 (100 µl per transformation). 500–4000 ng of prepared DNA (depending on the number of vectors used) was added to 100 µl protoplast suspension and incubated on ice for 2 min. Then 1 mL of transformation solution 2 was added to the mixture and incubated at room temperature for 20 min. After incubation, 5 mL of appropriate selective soft agar was added to the mixture and overlaid over prepared plates with corresponding selection agar. Plates were incubated at 28 °C for 4–6 days. When mycelia were visible on the plate, the transformants undergo two further selection steps to avoid false-positive transformants. The colonies are picked from the agar, placed on fresh selection agar plates, and grown for 3–5 days. For the preparation of liquid cultures, the transformants were grown on DPY agar plates for 5 days. The spores were used to inoculate 100 mL of DPY or CMP liquid medium.

9.5 Molecular Biology Methods

9.5.1 DNA and RNA Extraction

The different methods and kits used to purify and extract DNA/RNA are summarised in Table S6. The manufacturer's instructions and buffers were used. The concentration of DNA and RNA samples was determined with a DeNovix® DS-11+ Spectrophotometer.

Table S6 List of kits used for DNA/RNA extraction.

Source	DNA/RNA	Kit	Comment
E. coli	vector DNA	Nucleospin® Plasmid kit (*Machery-Nagel*)	from overnight culture
S. cerevisiae	vector DNA	Zymoprep Yeast Plasmid Miniprep II kit (*Zymo Research*)	after growing on SM-URA plates
A. oryzae NSAR1 transformants	genomic DNA	GeneElute™ Plant Genomic DNA Miniprep Kit (*SIGMA Life Science*)	from mycelia from appropriate plates
H. lienhwacheense	mRNA	RNA Clean and Concentrator™-5 (*Zymo Research*)	from liquid cultures under producing conditions
	mRNA to cDNA	High Capacity RNA-to-cDNA Kit (*Thermo Fisher Scientific*)	
PCR	PCR fragments	Nucleospin® Gel an PCR Clean-up kit (*Machery-Nagel*)	
agarose gel	linearized vectors		

9.5.2 Cloning Procedures

Polymerase Chain Reaction

PCR was used to amplify DNA Fragments from genomic DNA, cDNA or vectors. The proofreading Q5® 2x Master Mix (New England Biolabs) was used to obtain DNA fragments needed for further cloning procedure. The information provided by the manufacturer served as a template. The OneTaq® 2X Master Mix (New England Biolabs) was used to control if the GOI is present in a vector or gDNA. The information provided by the manufacturerserved as a template. For colony PCR one single colony was picked and prepared with OneTaq® 2X Master Mix (New England Biolabs). The initial heating temperature time was increased from 30 s to 7 min.

Agarose Gel Electrophoresis

Agarose gel electrophoresis was used to visualize DNA or RNA. Therefore, the gel was prepared with 0.5–2 % agarose in TAE-Buffer. 1 µl Roti®-Safe GelStain (Roth) was added to 25 mL agarose.The volume of 5 µl of DNA was mixed with 6 x loading buffer. When using OneTaq® 2X Master Mix no additional loading buffer was necessary. As a marker 2 µl of the 1 kb DNA Ladder (New England Biolabs) was used. The gel was run at 110 V and 400 mA for 25 min in a Bio-Rad gel chamber containing 0.5 % TAE-buffer. In case of large DNA fragments (> 5000 bp), for example after restriction digestion, 0.5 % Agarose gel was used, and the gel was run at 90 V for 40 min. The DNA was visualized with the Molecular Imager Gel doc XR+ (Bio-Rad) system under UV·light (312 nm).

Restriction Enzyme Digestion

All enzymes (Table S7) used in this work were purchased from New England Biolabs (Beverly, MA, USA) and used according to the manufacturer's instructions with appropriate buffers.

Table S7 List of enzymes.

Enzyme	Vector/Fragment	Digestion site
AscI	pTY GS *argB/ade/sC*	GG'CGCGCC
NotI-HF	pTY GS *argB/ade/sC*<br>pET-28a + lwmR4	GC'GGCCGC
NdeI	pET-28a + lwmR4	CA'TATG
NheI-HF	pET-28a + lwmR2	G'CTAGC
XhoI	pET-28a + lwmR2	C'TCGAG

Gateway Cloning

GatewayTM LR ClonaseTM II Enzyme mix kit (Invitrogen) was used to transfer genes from the entry vector to the destination vector. The manufacturer's instructions were followed. For *E. coli* Top10 transformation, the vector mixture (10 µl) was added to 50 µl competent cells.

DNA Sequencing

DNA samples were sequenced by Eurofins Genomics (Mix2Seq OVERNIGHT, Ebersberg).

10. LCMS Analysis for Gene Expression

10.1 Extraction of Fungal Liquid Cultures

After cultivation of liquid cultures, the biomass was homogenized with a blender and flirted by vacuum filtration in most cases (*H. lienhwacheense*, *A. oryzae* NSAR1 transformants of lienhwalide project). If not stated differently, the supernatant was acidified with 2 M HCl to pH 2–4. The media was extracted twice with an equal amount of ethyl acetate. The organic layers were dried with anhydrous magnesium sulfate ($MgSO_4$). The organic phase was removed with a rotary evaporator. The extract was then dissolved in an appropriate amount of solvent (methanol, acetonitrile or dichloromethane) filtered with glass wool and analysed by LCMS.

10.2 Analytical LCMS

Analytical LCMS consisted of a Waters 2767 autosampler, Waters 2545 pump, a Phenomenex Kinetex column (2.6 µm, C_{18}, 100 Å, 4.6 x 100 mm), a Phenomenex Security Guard precolumn (Luna, C_5, 300 Å) was used with a flow rate of 1 mL/min. The equipped detectors were a diode array detector (Waters 2998) in the range 210 to 600 nm and an ELSD detector (Waters 2424) together with a mass spectrometer, Waters SQD-2 mass detector (ES+ and ES-, 150 to 1000 *m/z*). For elution, a solvent gradient was run for 15 min starting at 10 % acetonitrile/90 % HPLC grade water (0.05 % formic acid) and ramping to 90 % acetonitrile water (0.045 % formic acid).

10.3 Preparative LCMS

Isolation of compounds was achieved using a Waters 2767 autosampler, a Waters 2454 pump system using a flowrate of 20 mL/min and a Waters mass-directed autopurification system (equipped with a Phenomenex Kinetex Axia column [5µ; C_{18}; 100 Å; 21.2 x 250 mm] and a Phenomenex Security Guard column [Luna C_5; 300 Å]). The flow was split after the column (100:1). The major part of the flow was collected with the fraction system and the other minor part was analysed by the equipped detectors. The minority flow was compensated with a mixture of acetonitrile and water (1:1) at 0.8 mL/min. Then, the minority flow was analysed using the same detectors as described previously. A solvent gradient was starting at 10 % acetonitrile/90 % HPLC grade water (0.05 % formic acid) and ramping to 90 % acetonitrile water (0.045 % formic acid). The compounds were collected according to the mass analysis in glass tubes. After combining identical fractions from repeating LCMS runs, residual acetonitrile was removed *in vacuo*. The remaining aqueous suspension was frozen and lyophilised (Alpha 1-4 LDplus, Martin Christ).

11. NMR Spectral Data

11.1 Nuclear Magnetic Resonance Spectroscopy (NMR)

Compound samples were measured on one of the following spectrometers: Bruker Ascend 600 MHz, Bruker Ultrashield 500 MHz, Bruker Ascend 400 MHz, Bruker Ultrashield 400 MHz. Data was acquired at 400/500/600 MHz. Chemical shifts are defined by parts per million (ppm) relative to tetramethysilane standard. Raw data was referenced by the deuterated solvent used. 2D experiment data were obtained for complete structural elucidation including Correlation Spectroscopy (COSY), Heteronuclear Single Quantum Coherence (HSQC), and Heteronuclear Multiple Bond Correlation (HMBC). The data was analysed using the MestReNova 14.2.3 software (MestreLab Research).

NMR spectra were recorded with an Avance III 700 spectrometer (Bruker, Billerica, MA, USA; ^{1}H NMR: 700 MHz and ^{13}C NMR: 175 MHz) and an Avance III 500 spectrometer (Bruker, Billerica, MA, USA, ^{1}H NMR: 500 MHz, and ^{13}C NMR: 125 MHz).

11.2 Lienhwalide A 5

Table S8 NMR data (^{1}H 500 MHz, ^{13}C 125 MHz) of **5** in chloroform-*d*.

Pos	δ_C, mult.	δ_H, mult.	COSY	HMBC	ROESY
1	20.8, CH_3	1.23, d (6.1)	2	2 ,3	2, 3
2	68.5, CH	3.66, dqd (9.0,6.1,3.7)	1, 3	11	1, 3, 11
3	42.3, CH_2	2.65, m	2, 11	1, 2, 4, 5, 10	5, 1, 2
4	145.3, C				
5	115.3, CH	6.77, s		3, 4, 6, 7, 10	3
6	161.9, C				
7	173.8, C				
8	164.9, C				
9	113.6, CH	6.89, s		6, 7, 8, 10, 11	
10	128.5, C				
11	73.8, CH	5.37, m	12a, 12b, (3)	4, 10, 12, 13	12a, 12b, 3
12	30.5, CH_2	3.21, dd (13.3,4.3)<br>3.05, dd (13.3,6.3)	11, 12b<br>11, 12a	10, 11, 13, 14, 16<br>10, 11, 13, 14, 16	12b, 11<br>12a, 11
13	137.2, C				
14	166.1, C				
15	165.1, C				
16	138.7, C				
17	118.2, CH	6.42, dt (16.0,1.6)	18	13, 15, 16, 18, 19	12a, 12b
18	147.7, CH	7.14, dt (16.0,6.5)	17, 19	16, 19, 20	19
19	27.2, CH_2	2.25, qdd (7.5,6.5,1.6)	18, 20	17, 18, 20	20
20	12.3, CH_3	1.06, t (7.5)	19	18, 19	19

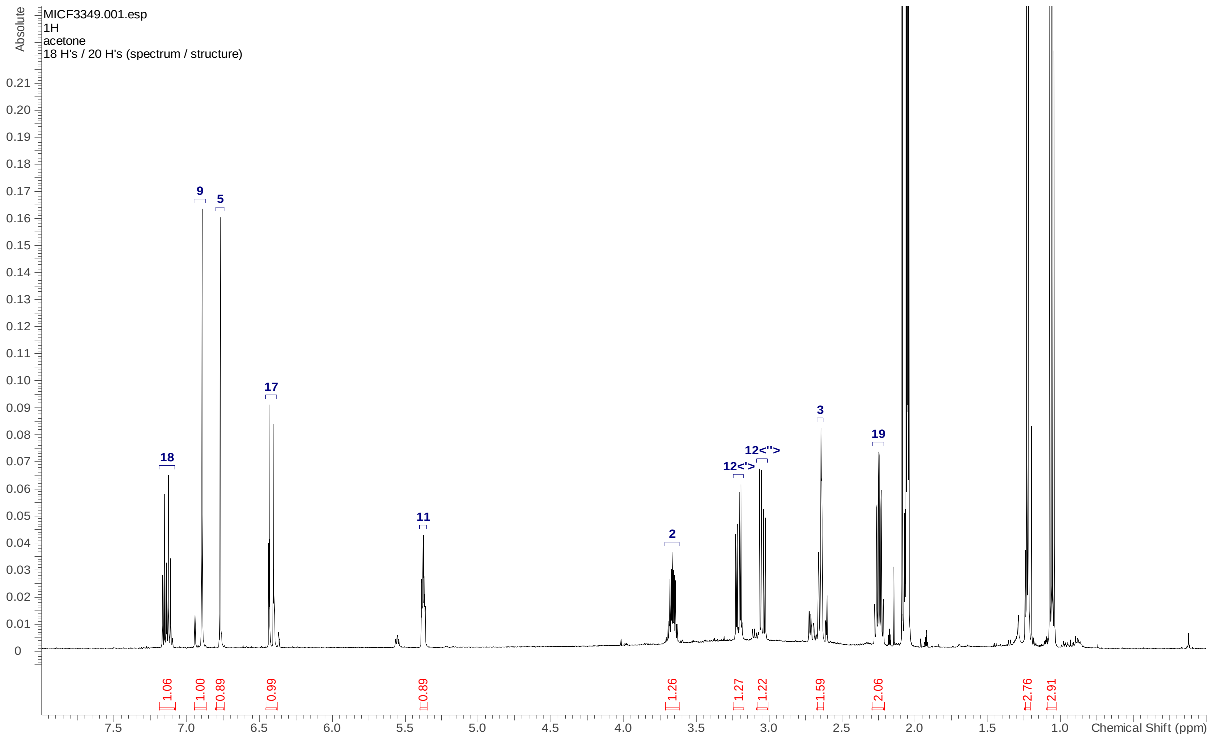

Figure S13. ^{1}H NMR spectrum (500 MHz, acetone-d_6) of liehnwalide A **5**.

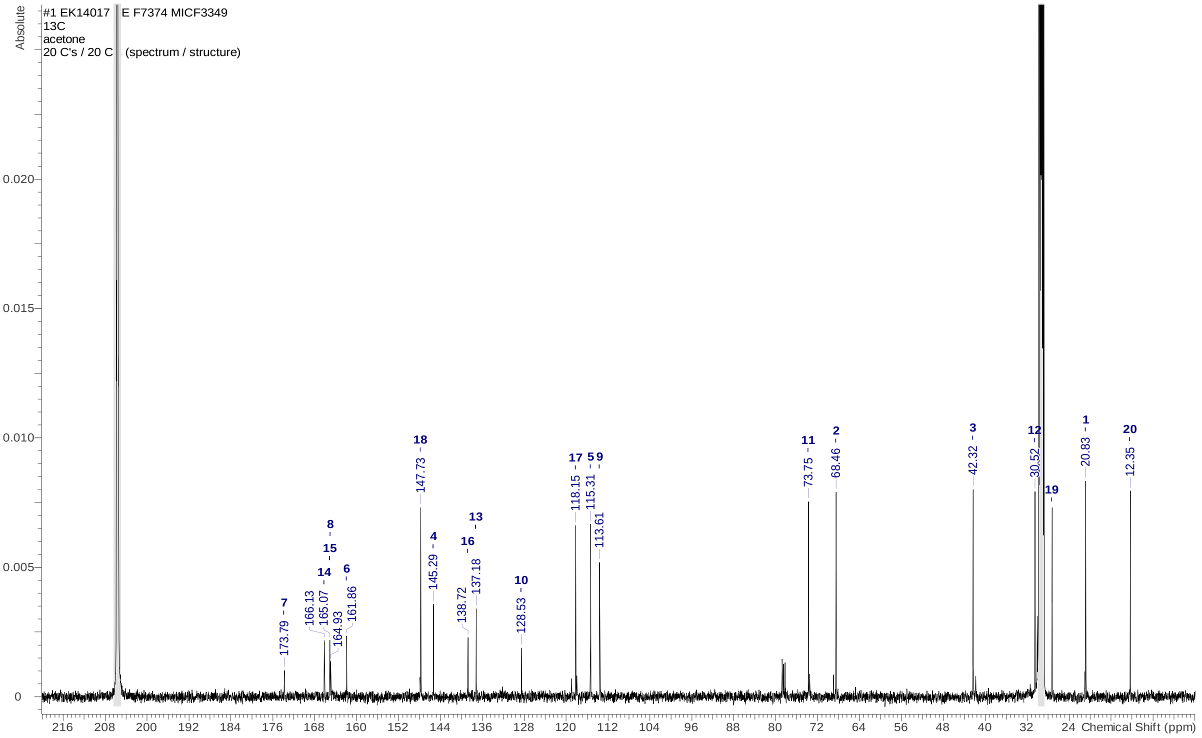

Figure S14. ^{13}C NMR spectrum (125 MHz, acetone-d_6) of liehnwalide A **5**.

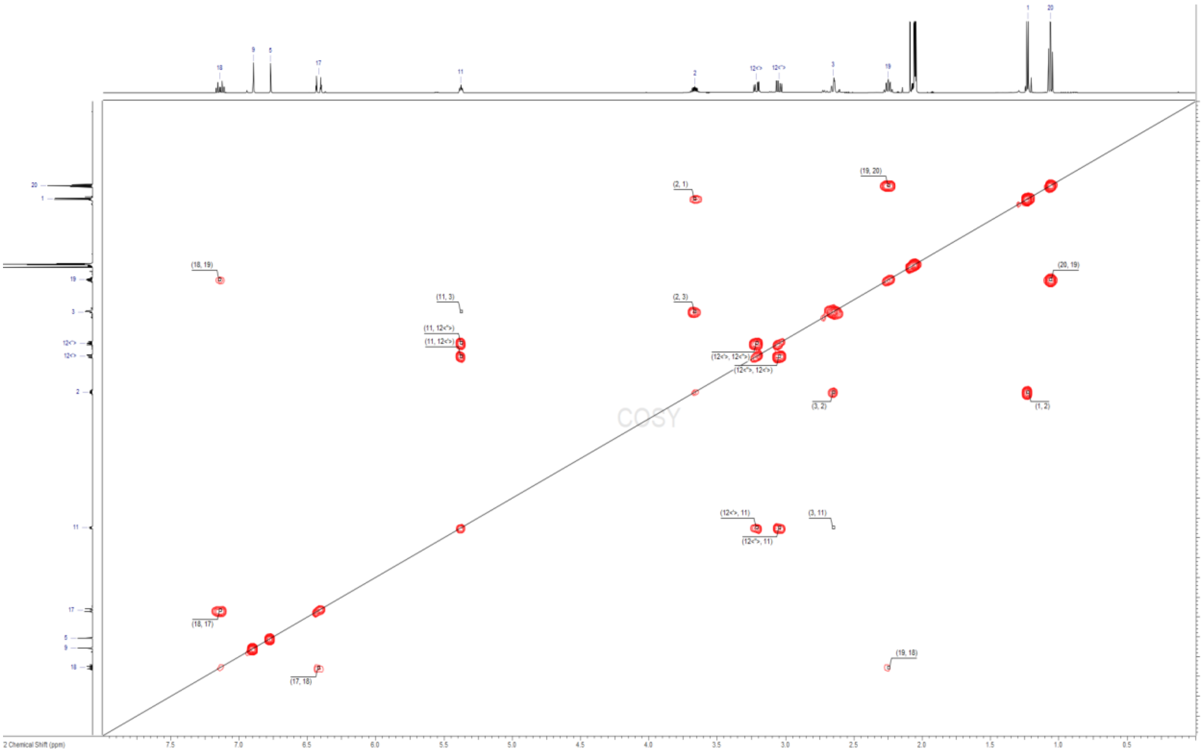

Figure S15. COSY NMR spectrum (500 MHz, acetone-d_6) of liehnwalide A **5**.

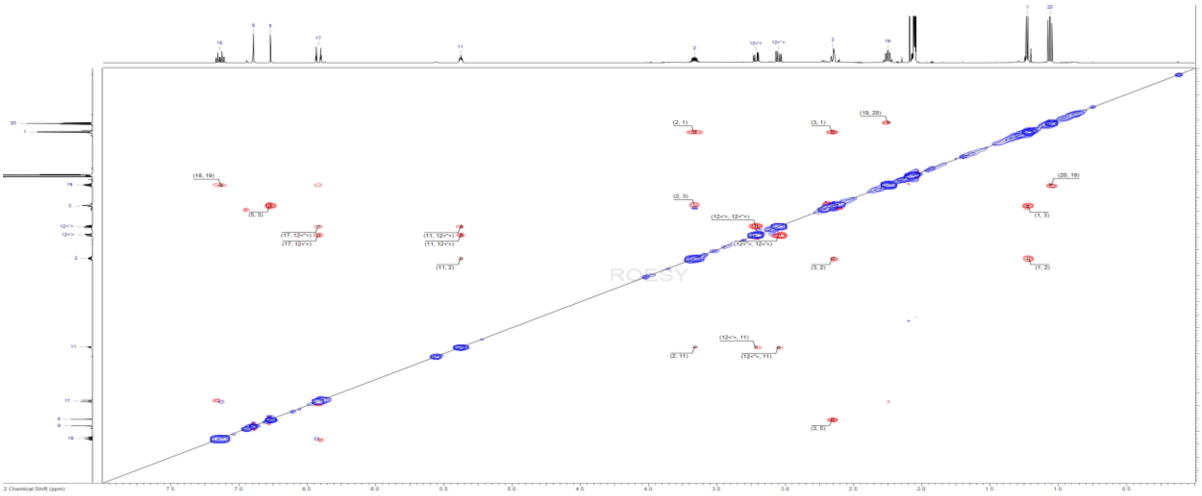

Figure S16. ROESY NMR spectrum (500 MHz, acetone-d_6) of liehnwalide A **5**.

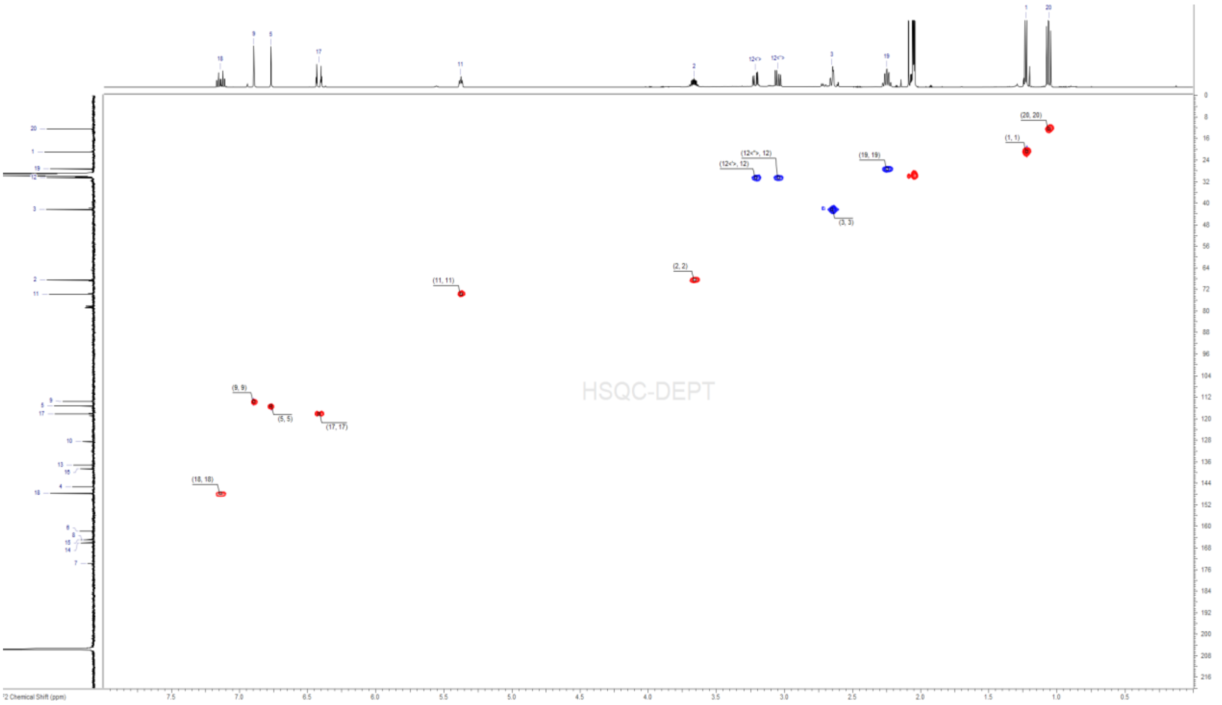

Figure S17. HSQC NMR spectrum (500 MHz, acetone-d_6) of liehnwalide A **5**.

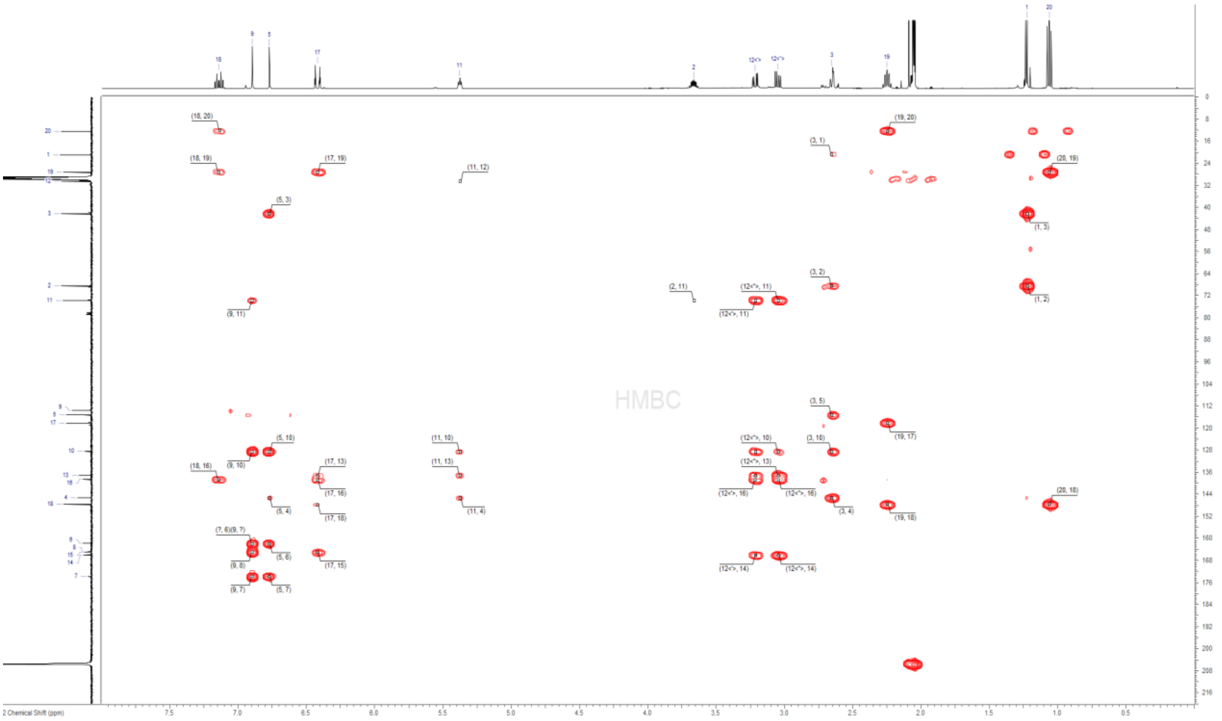

Figure S18. HMBC NMR spectrum (500 MHz, acetone-d_6) of liehnwalide A **5**.

11.3 Lienhwalide B 9

Table S9. NMR data (^{1}H 700 MHz, ^{13}C 175 MHz) of **9** in chloroform-*d*.

Pos	δ_C, mult.	δ_H, mult.	COSY	HMBC	ROESY
1	20.9, CH_3	1.25, d (6.5)	2	2, 3	2, 3a, 3b
2	68.6, CH	3.65, dqd (10.5,6.5,1.6)	1, 3	1, 4, 11	1, 3a, 11
3	42.4, CH_2	2.77, m	2, 3b<br>2, 3a	1, 2, 4, 5, 10<br>2, 4, 5, 10	1<br>1, 2, 5
4	139.0, C				
5	114.9, CH	6.71, s	3b, 6OMe	3, 4, 5, 6, 7	3a, 3b, 6OMe
6	153.3, C				
7	147.1, C				
8	159.2, C				
9	161.2, C				
10	127.5, C				
11	73.5, CH	5.52, dd (7.0,3.8)	12a, 12b	2, 4, 9, 10, 12 ,13	2, 12a, 12b
12	29.1, CH_2	3.21, dd (13.4, 3.8)<br>2.99, dd (13.4, 7.0)	11, 12b<br>11, 12a	10, 11, 13, 14, 16<br>10, 11, 13, 14, 16	11, 17<br>11
13	136.0, C				
14	165.8, C				
15	164.6, C				
16	138.7, C				
17	117.0, CH	6.35, d (15.9)	18, 19	13, 15, 16, 18, 19	12a, 12b, 19
18	149.0, CH	7.22, dt (15.9, 6.7)	17, 19	16, 19, 20	19
19	27.3, CH_2	2.25, dq (7.3, 6.7)	18, 20	17, 18, 20	17, 20
20	12.4, CH_3	1.08, t (7.3)	19	18, 19	19
6-OMe	56.9, CH_3	4.02, s	5	6	5

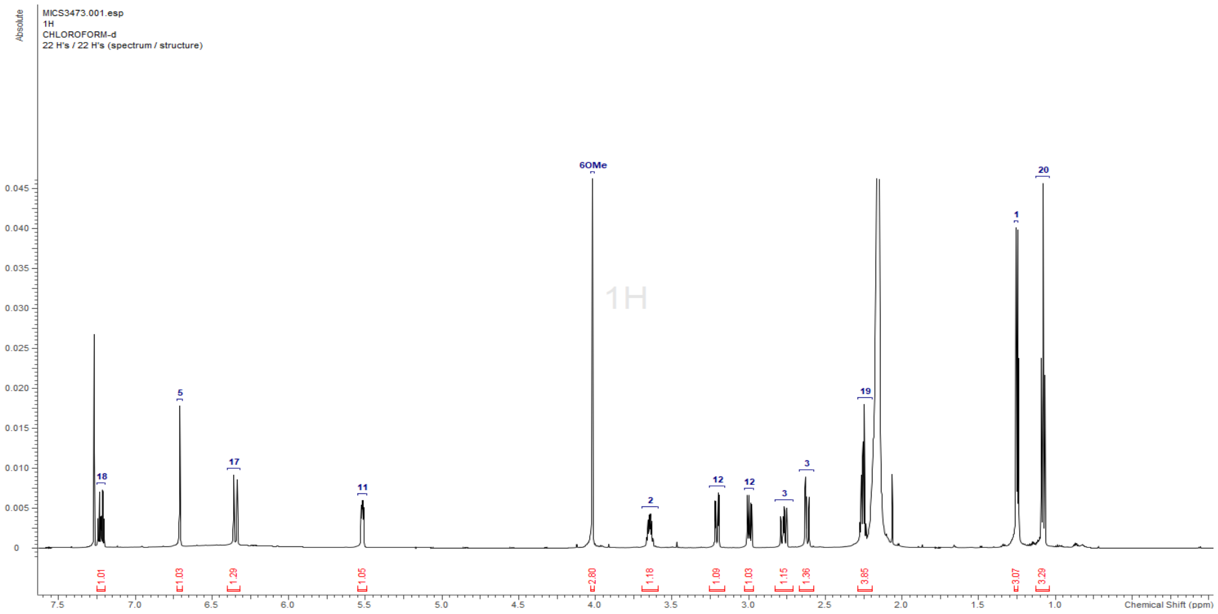

Figure S19. ^{1}H NMR spectrum (500 MHz, chloroform-d_6) of liehnwalide B **9**.

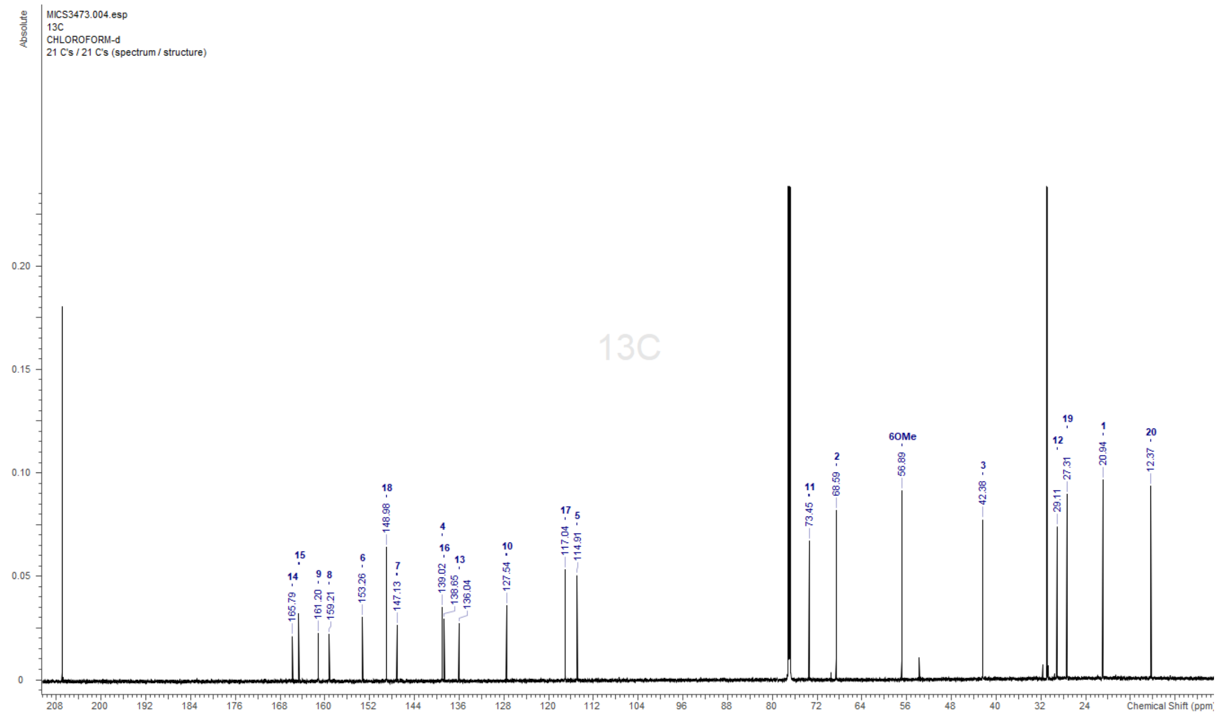

Figure S20. ^13^C NMR spectrum (125 MHz, chloroform-d_6) of liehnwalide B **9**.

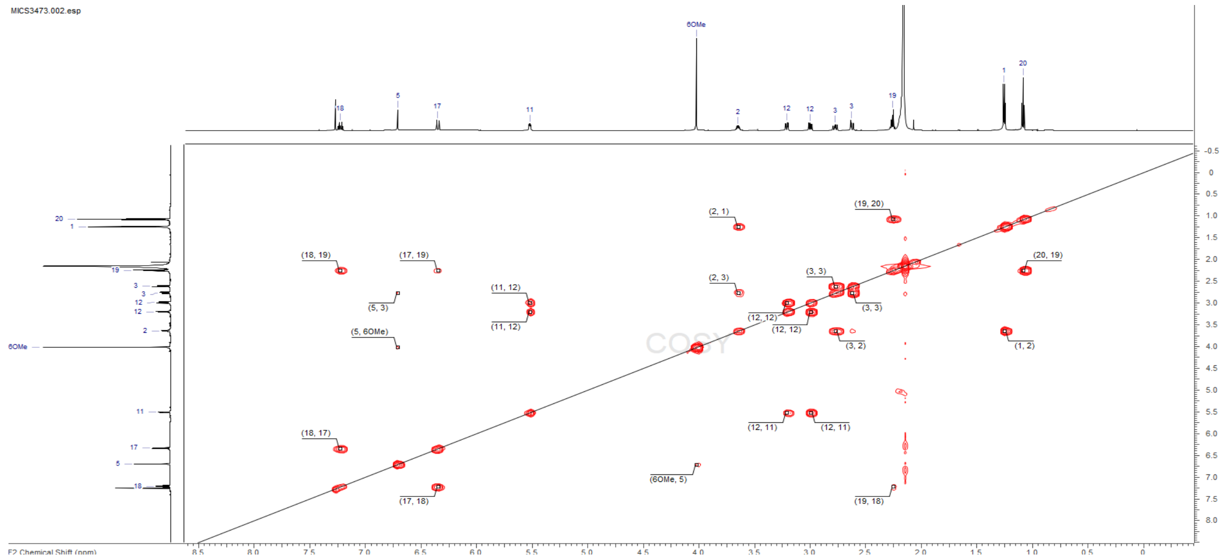

Figure S21. COSY NMR spectrum (500 MHz, chloroform-d_6) of liehnwalide B **9**.

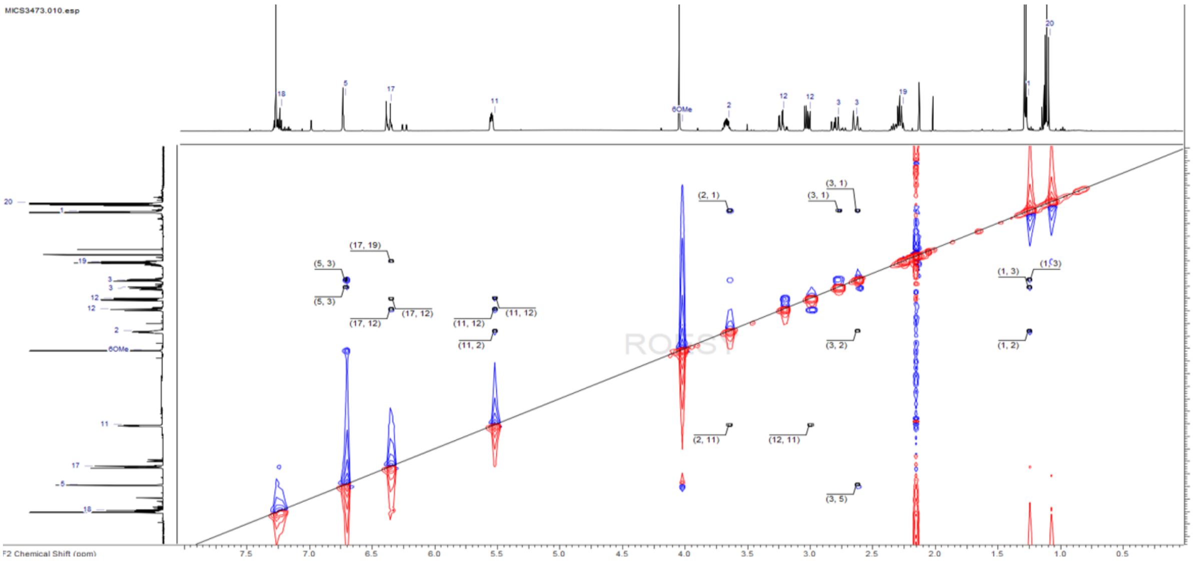

Figure S22. ROESY NMR spectrum (500 MHz, chloroform-d_6) of liehnwalide B **9**.

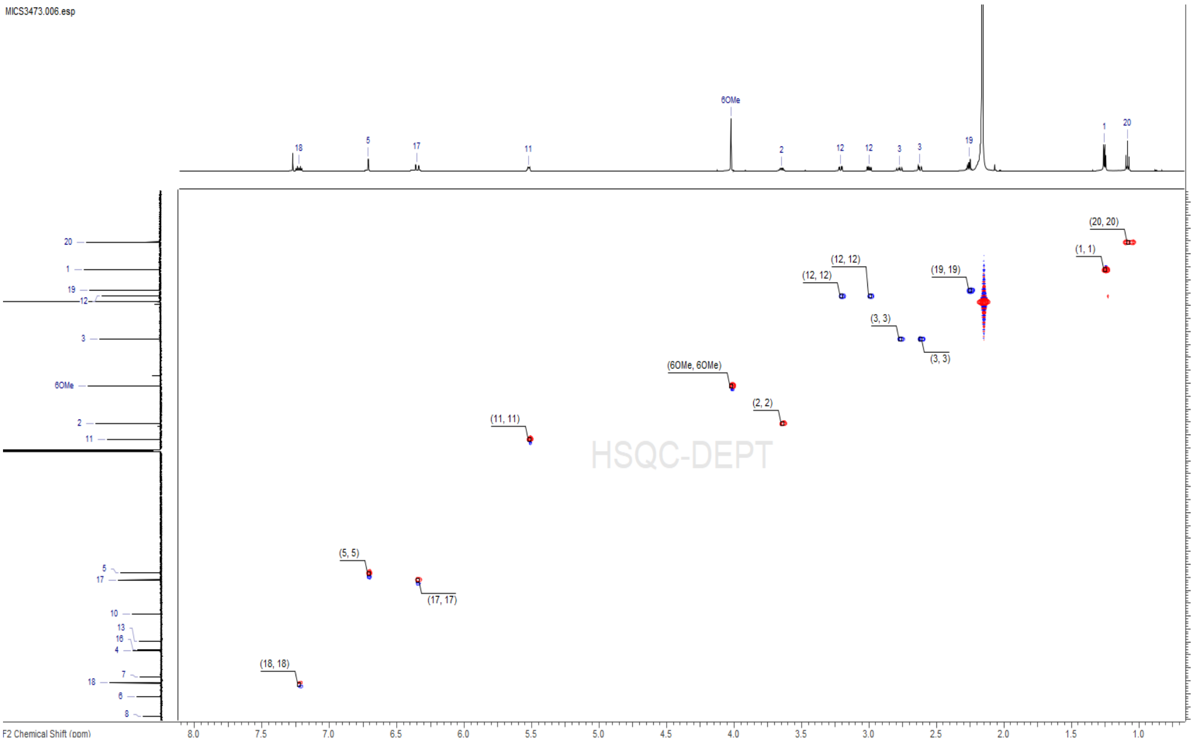

Figure S23. HSQC NMR spectrum (500 MHz, chloroform-d_6) of liehnwalide B **9**.

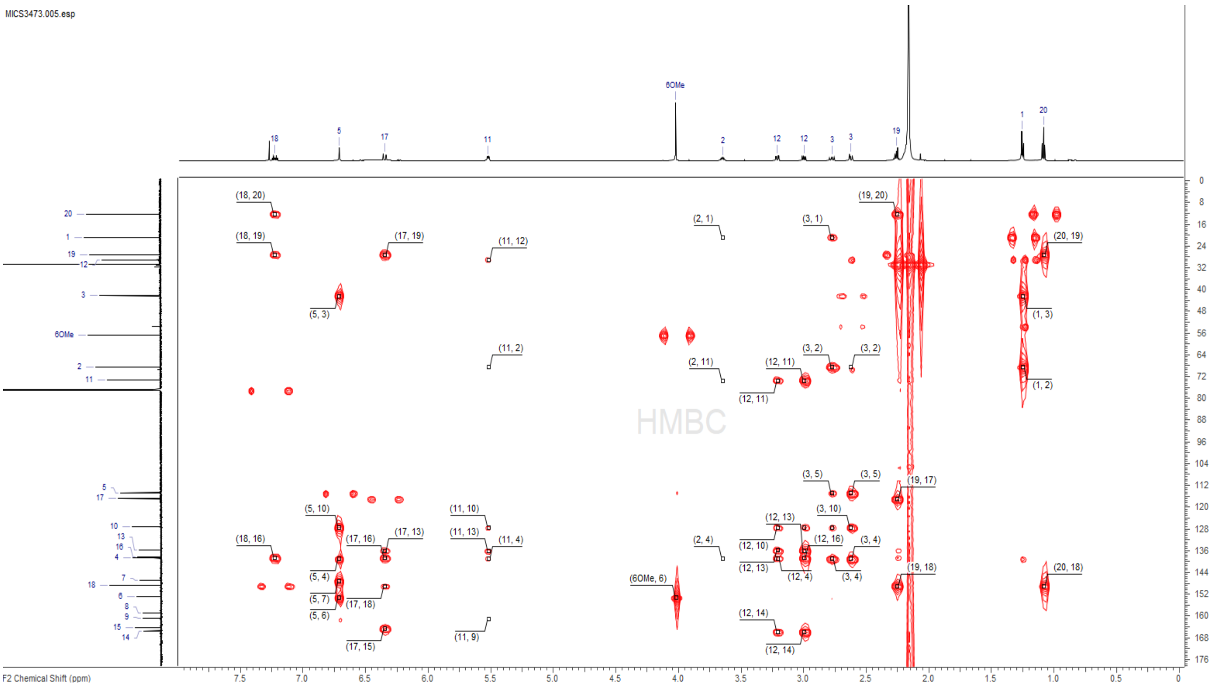

Figure S24. HMBC NMR spectrum (500 MHz, chloroform-d_6) of liehnwalide B **9**.

11.4 Lienhwalide C 10

Table S10. NMR data (^{1}H 500 MHz, ^{13}C 125 MHz) of **5** in chloroform-*d*.

Pos	δ_C, mult.	δ_H, mult.	COSY	HMBC	ROESY
1	21.4, CH_3	1.22, d (6.1)	2	2, 3	2, 3
2	69.7, CH	3.60 , dqd (10.7,6.1,1.0)	1, 3	1, 4, 11	1, 3, 11
3	36.7, CH_2	2.42, m	2, 11	1, 2, 4, 5, 10	1, 2, 5
4	127.0, C				
5	102.7, CH	6.17, s	3	3, 6, 7, 10	3, 6OMe
6	145.7, C				
7	130.6, C				
8	140.5, C				
10	115.4, C				
11	72.4, CH	5.25, m	3, 12a, 12b	4, 8, 10, 12, 13	2, 12a, 12b
12	29.6, CH_2	3.35, dd (13.4,3.7)<br>3.07, dd (13.4,6.4)	11, 12b<br>11, 12a	10, 11, 13, 14, 16<br>10, 11, 13, 14, 16	12b, 11, 17<br>12a, 11, 17
13	137.0, C				
14	166.0, C				
15	165.0, C				
16	138.6, C				
17	117.6, CH	6.33, dt (16.0,1.6)	18, 19	13, 15, 16, 18, 19	12a, 12b, 19
18	147.8, CH	7.18, dt (16.0, 6.7)	17, 19	16, 19, 20	19
19	27.3, CH_2	2.24, m	17, 18, 20	17, 18, 20	17, 20
20	12.5, CH_3	1.08, t (7.5)	19	18, 19	19
6-OMe	56.0, CH_3	3.85, s		6	5

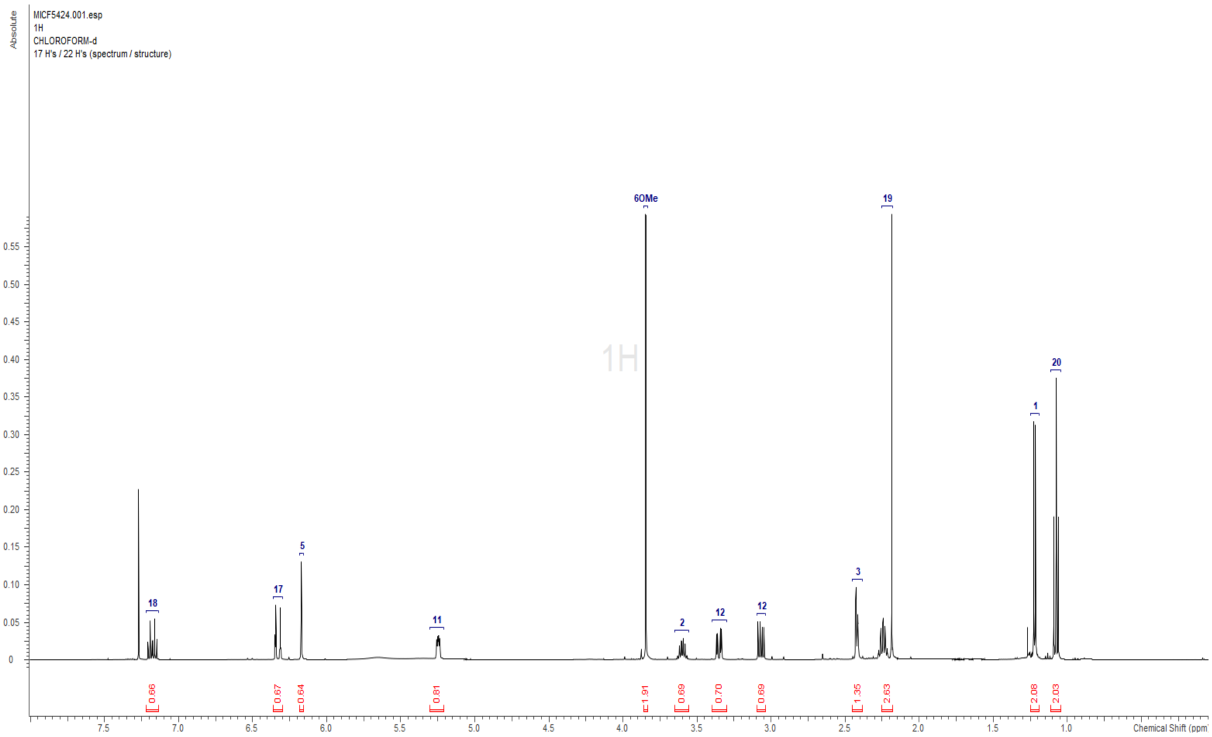

Figure S25. ^{1}H NMR spectrum (500 MHz, chloroform-d_6) of liehnwalide C **10**.

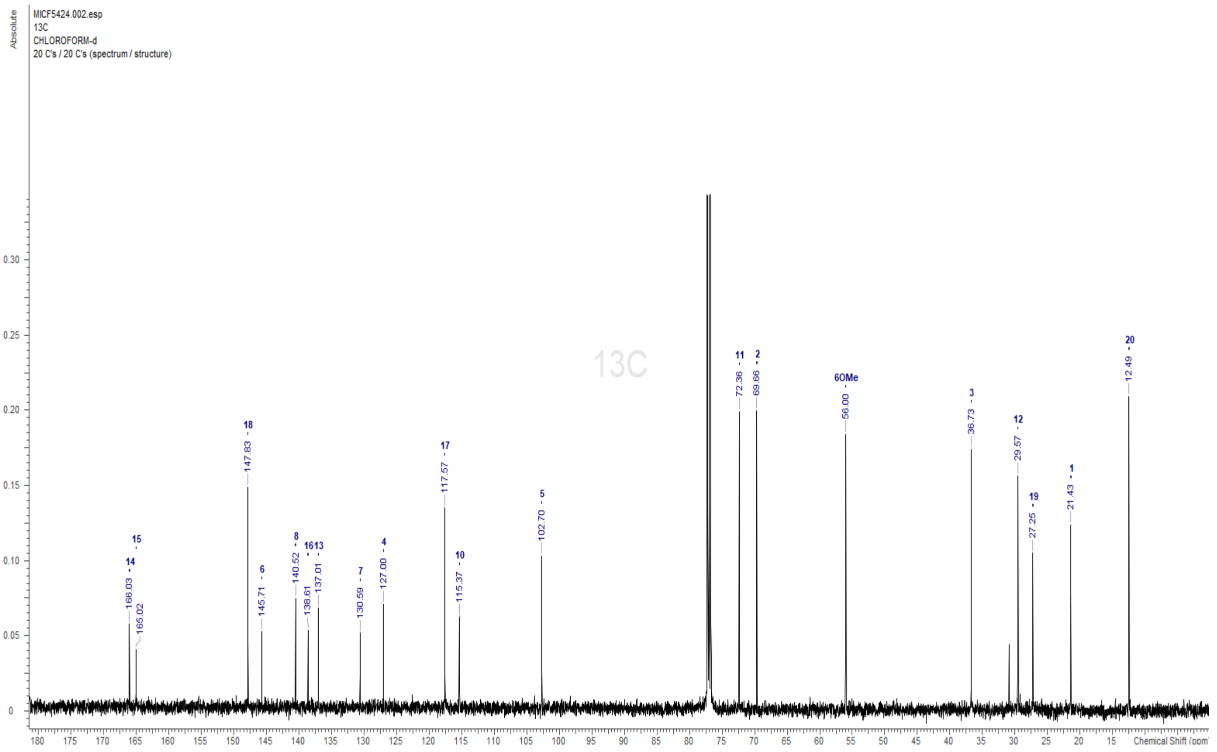

Figure S26. ^{13}C NMR spectrum (125 MHz, chloroform-d_6) of liehnwalide C **10**.

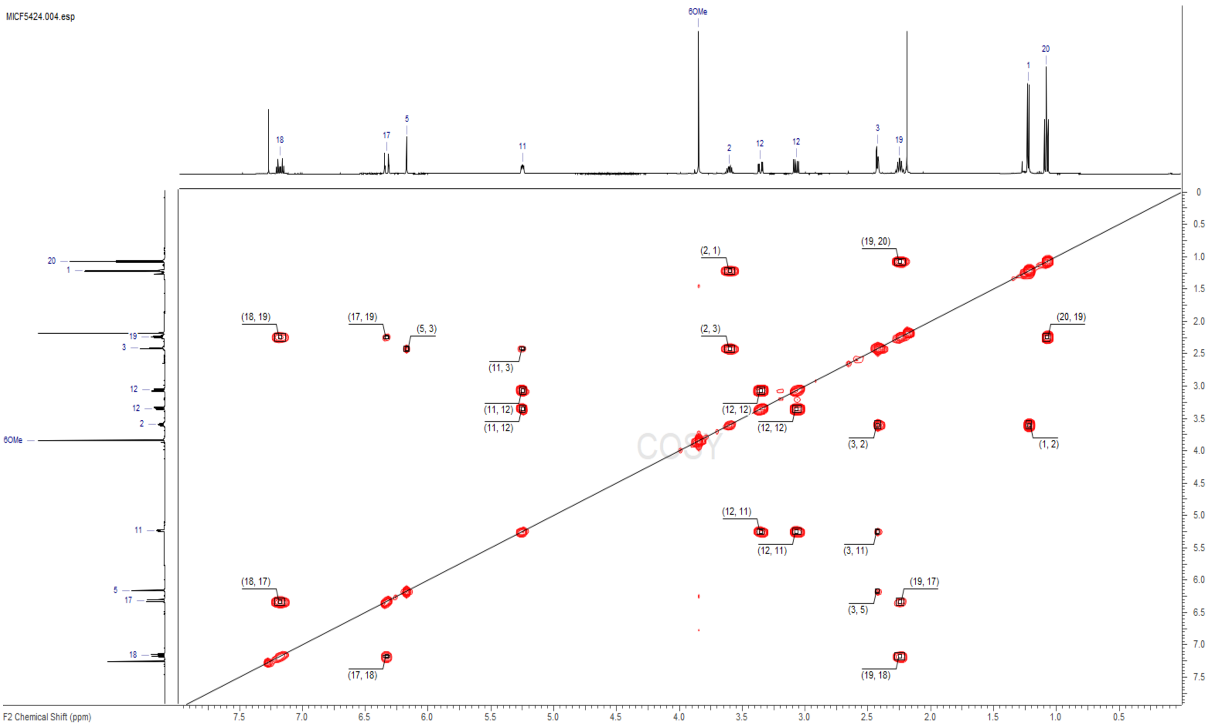

Figure S27. COSY NMR spectrum (500 MHz, chloroform-d_6) of liehnwalide C **10**.

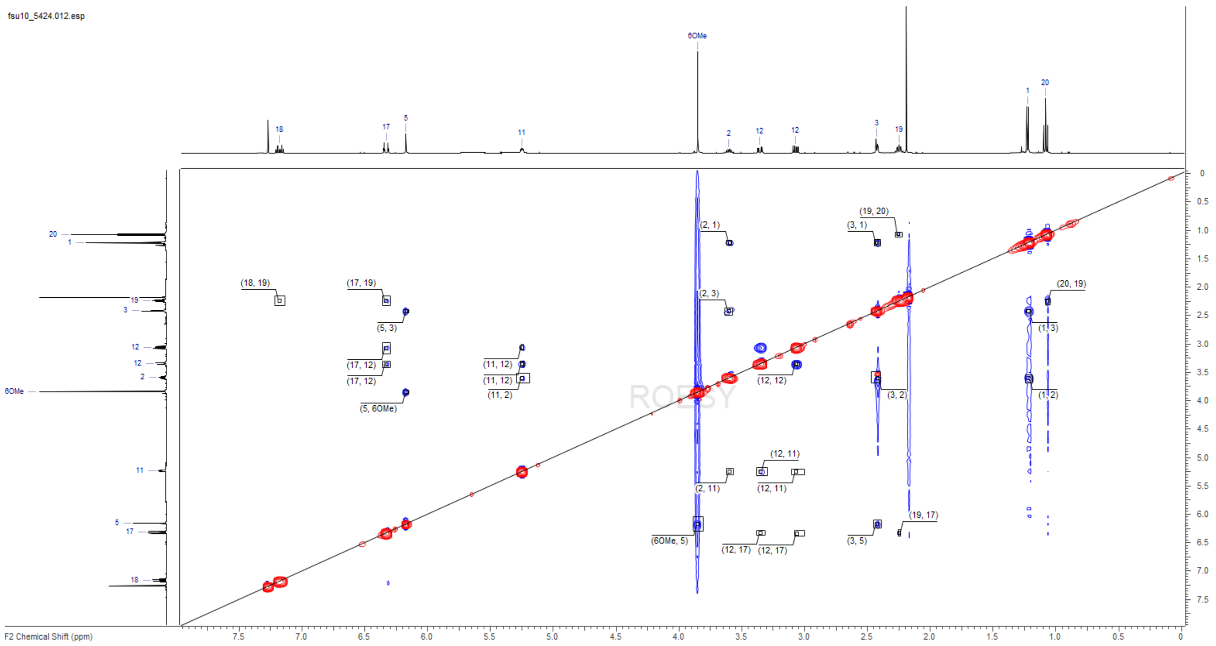

Figure S28. ROESY NMR spectrum (500 MHz, chloroform-d_6) of liehnwalide C **10**.

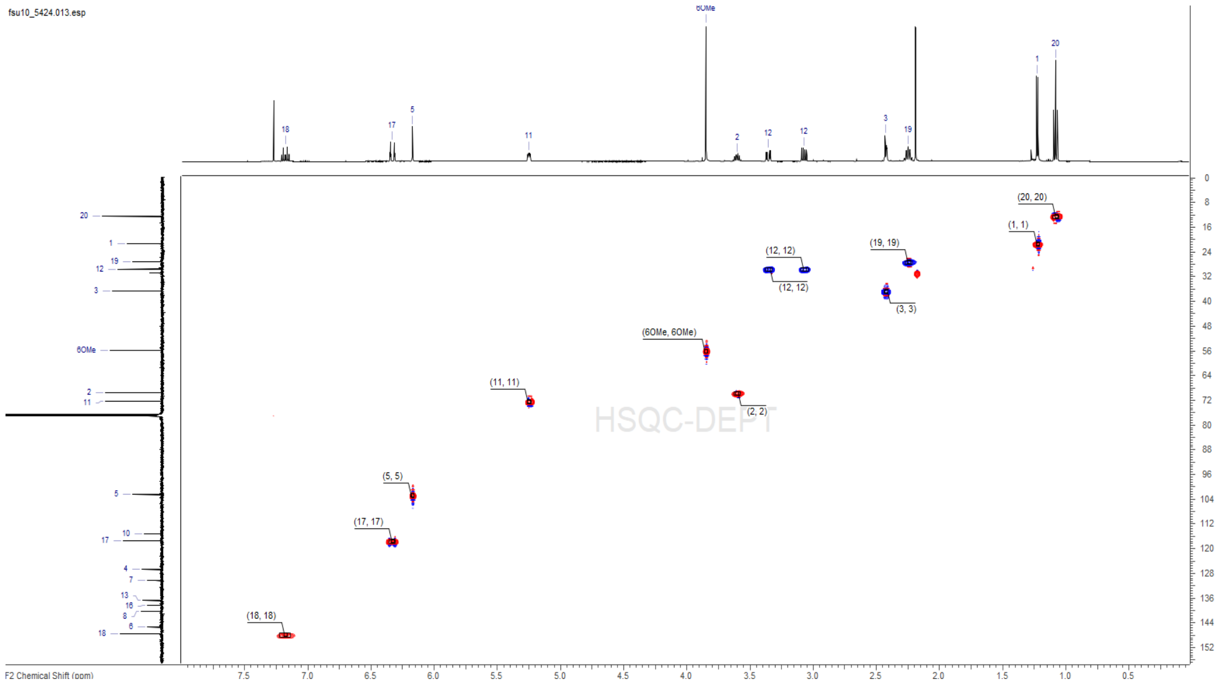

Figure S29. HSQC NMR spectrum (500 MHz, chloroform-d_6) of liehnwalide C **10**.

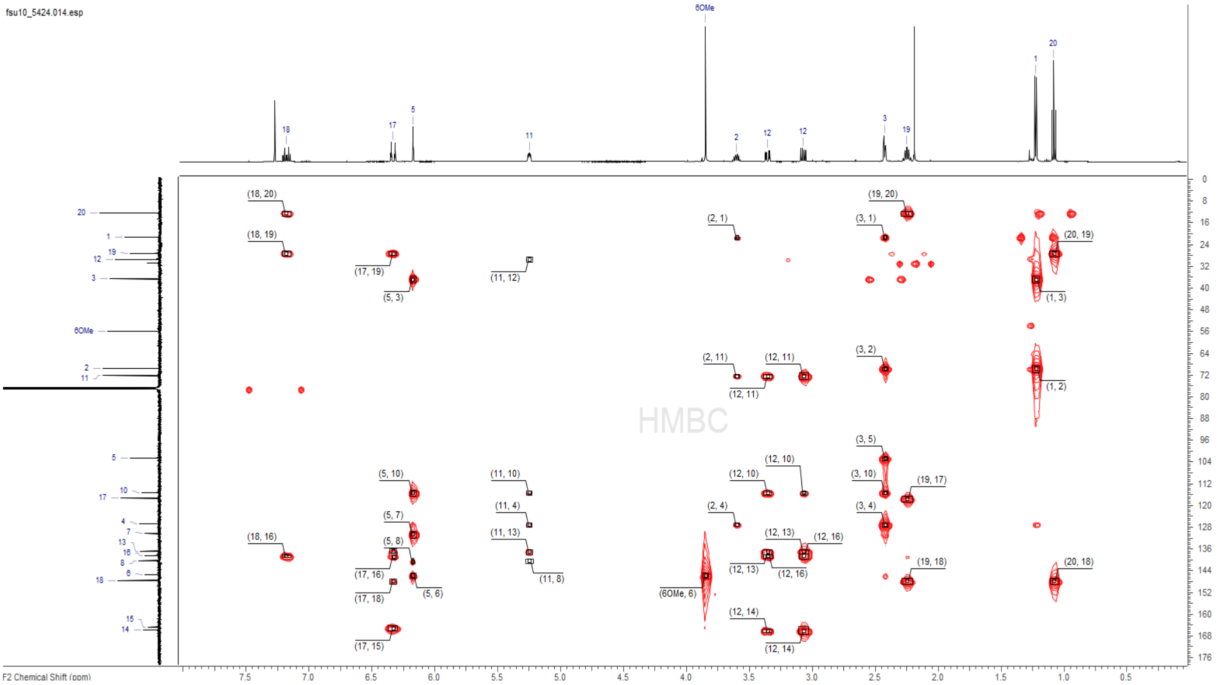

Figure S30. HMBC NMR spectrum (500 MHz, chloroform-d_6) of liehnwalide C **10**.

11.5 Lienhwalide D 11

Table S11. NMR data (^{1}H 700 MHz, ^{13}C 175 MHz) of **11** in pyridine-d_5.

Pos	δ_C, mult.	δ_H, mult.	COSY	HMBC	ROESY
1	18.1, CH_3	1.52, d (6.2)	2	2, 3	2, 3
2	73.1, CH	3.86, q (6.2)	1, 3	1, 3, 11	1, 3, 11
3	68.9, CH	4.47, s	2, 5	1, 2, 4, 5, 10	1, 2, 5
4	129.7, C				
5	104.6, CH	6.71, s	3, 6OMe	3, 4, 6, 7, 10	3, 6OMe
6	148.4, C				
7	135.4, C				
8	144.2, C				
9					
10	117.2, C				
11	73.3, CH	5.77, dd (6.5,4.0)	12a, 12b	4, 8, 10, 12, 13	2, 12a, 12b > 17
12	30.6, CH_2	3.51, dd (13.2,4.0)<br>3.45, dd (13.2,6.5)	11, 12b<br>11, 12a	10, 11, 13, 14, 16<br>10, 11, 13, 14, 16	11, 12, 17<br>11, 12a, 17
13	138.7, C				
14	167.2, C				
15	166.1, C				
16	139.2, C				
17	118.6, CH	6.58, d (15.9)	18, 19	13, 15, 16, 18, 19	12a, 12b, 19 > 11
18	146.6, CH	7.25, dt (15.9,6.7)	17, 19	16, 19, 20	19
19	27.4, CH_2	2.10, m	17, 18, 20	17, 18, 20	17, 18, 20
20	12.8, CH_3	0.94, t (7.4)	19	18, 19	19
6-OMe	55.9, CH_3	3.71, s	5	6	5

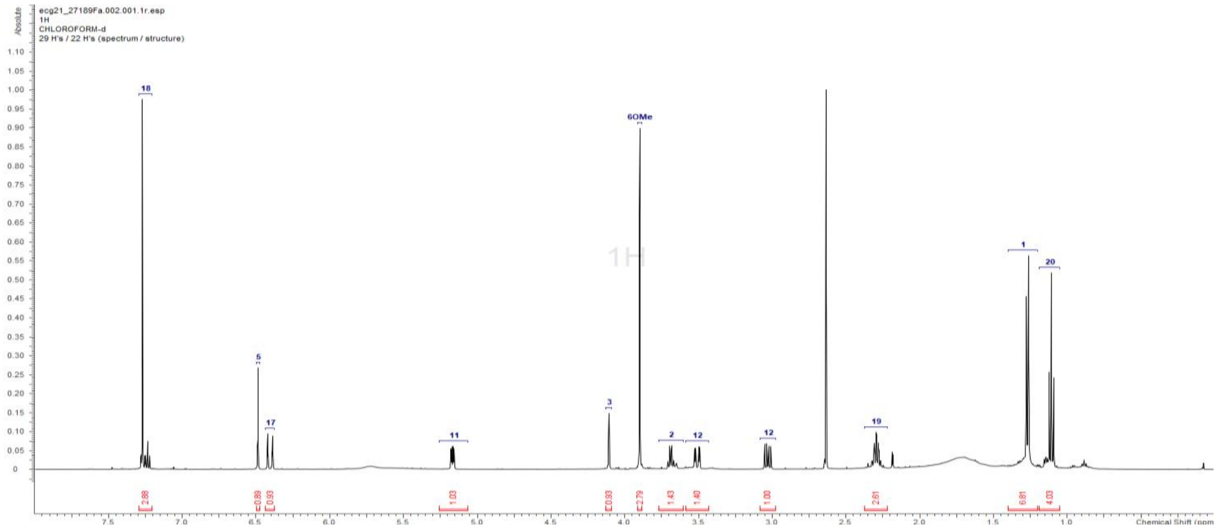

Figure S31. ^{1}H NMR spectrum (500 MHz, chloroform-d_6) of liehnwalide D **11**.

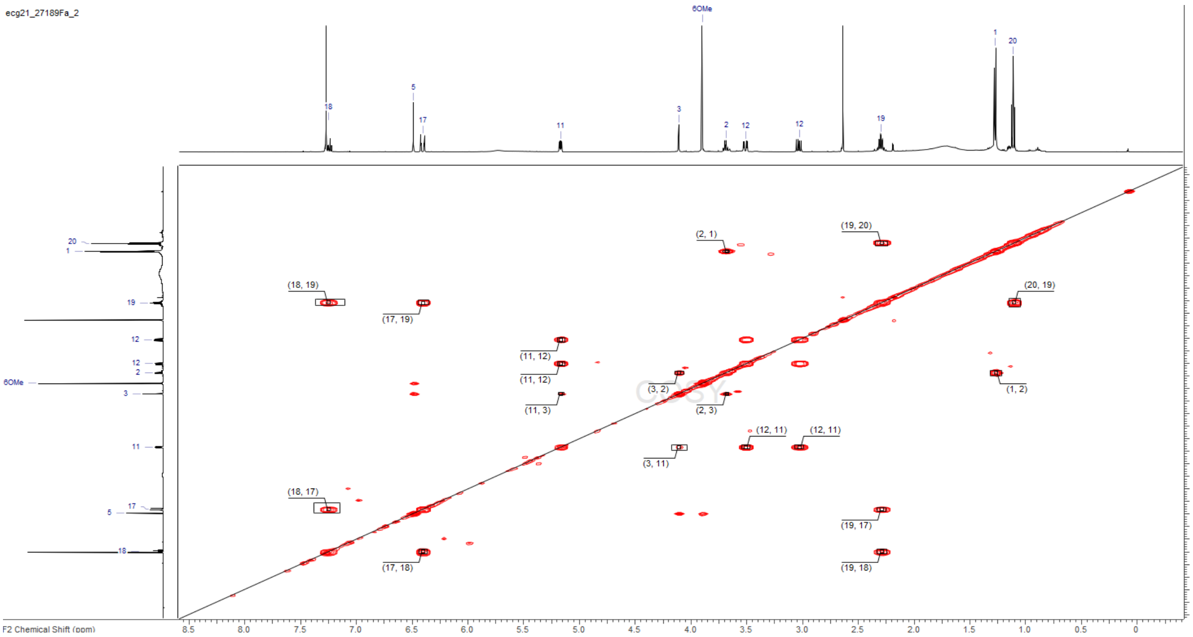

Figure S32. COSY NMR spectrum (500 MHz, chloroform-d_6) of liehnwalide D **11**.

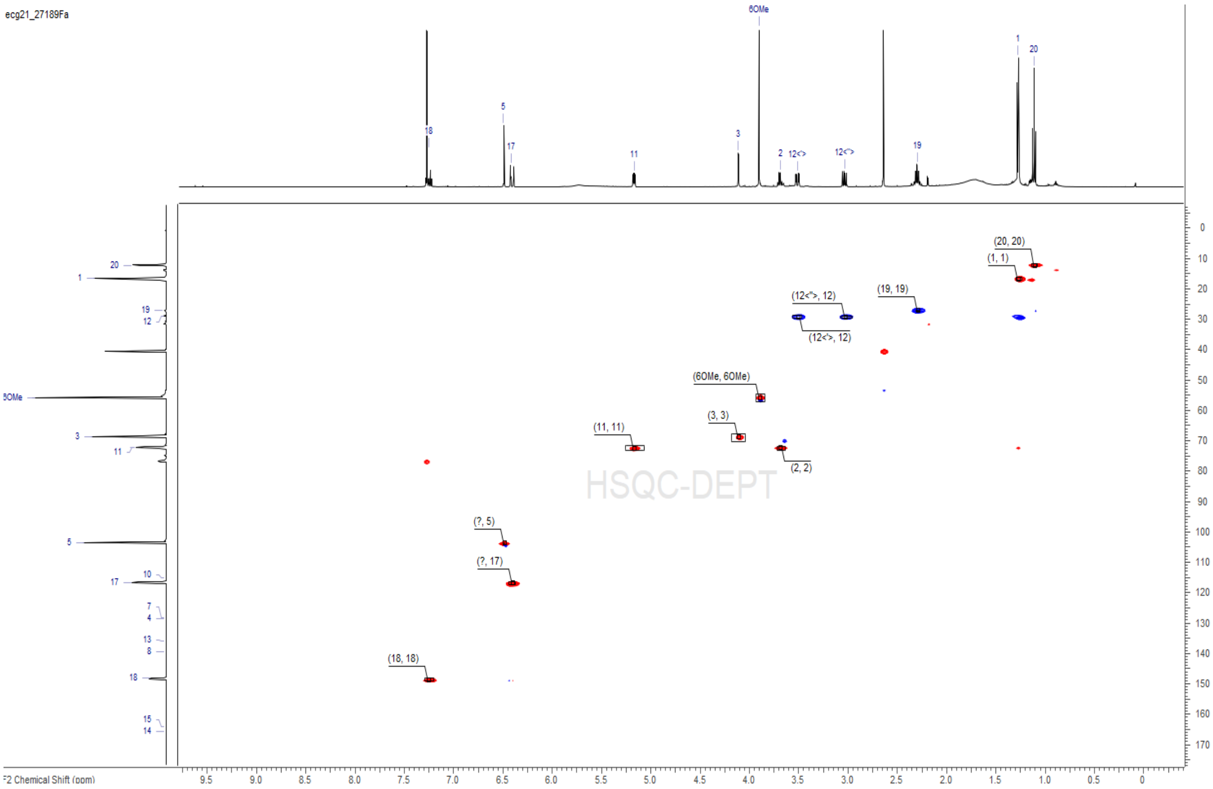

Figure S33. HSQC NMR spectrum (500 MHz, chloroform-d_6) of liehnwalide D **11**.

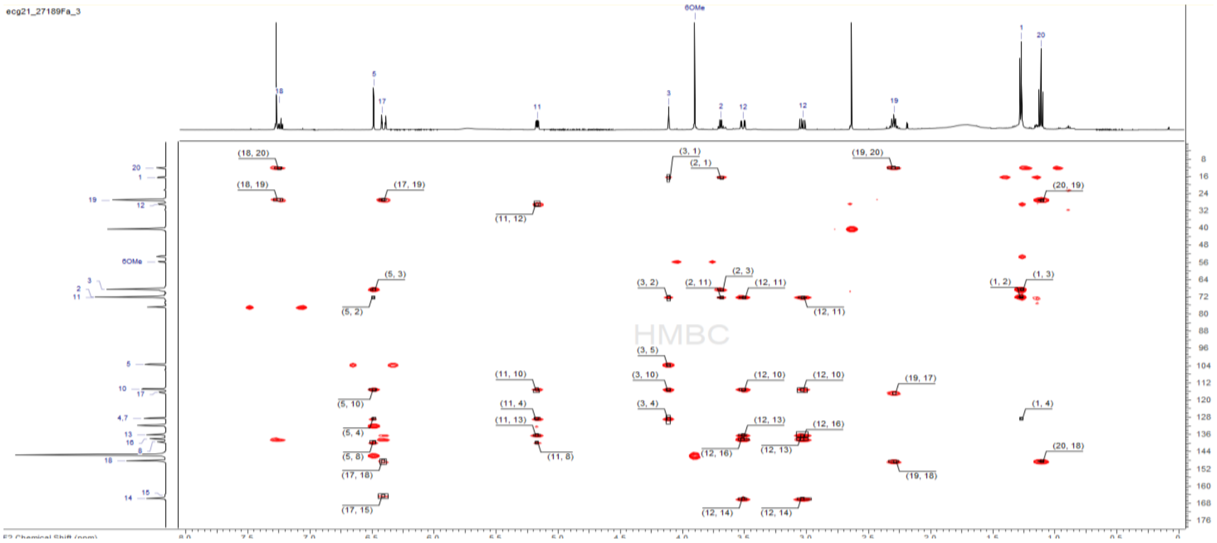

Figure S34. HMBC NMR spectrum (500 MHz, chloroform-d_6) of liehnwalide D **11**.

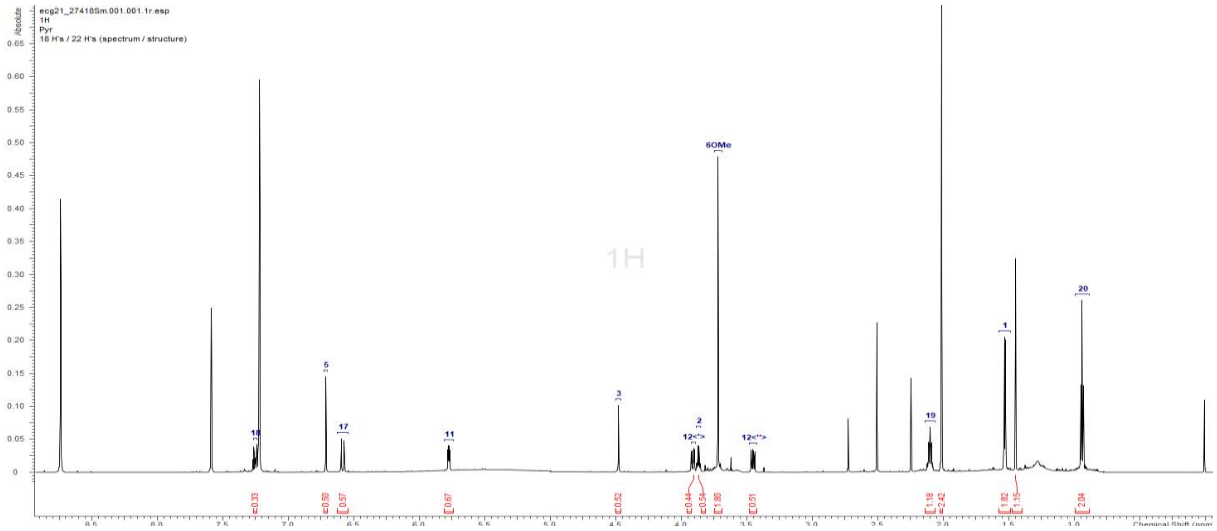

Figure S35. ^{1}H NMR spectrum (700 MHz, pyridine-d_5) of liehnwalide D **11**.

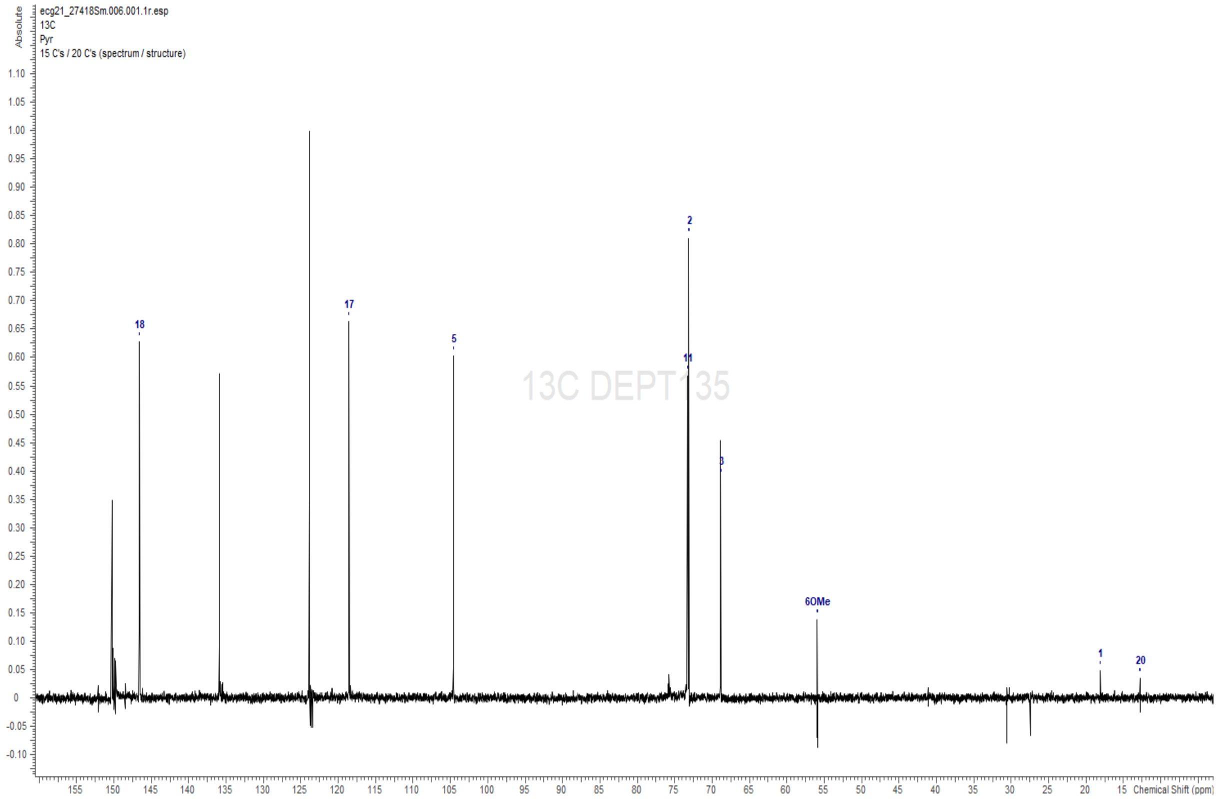

Figure S36. ^{13}C NMR spectrum (175 MHz, pyridine-d_5) of liehnwalide D **11**.

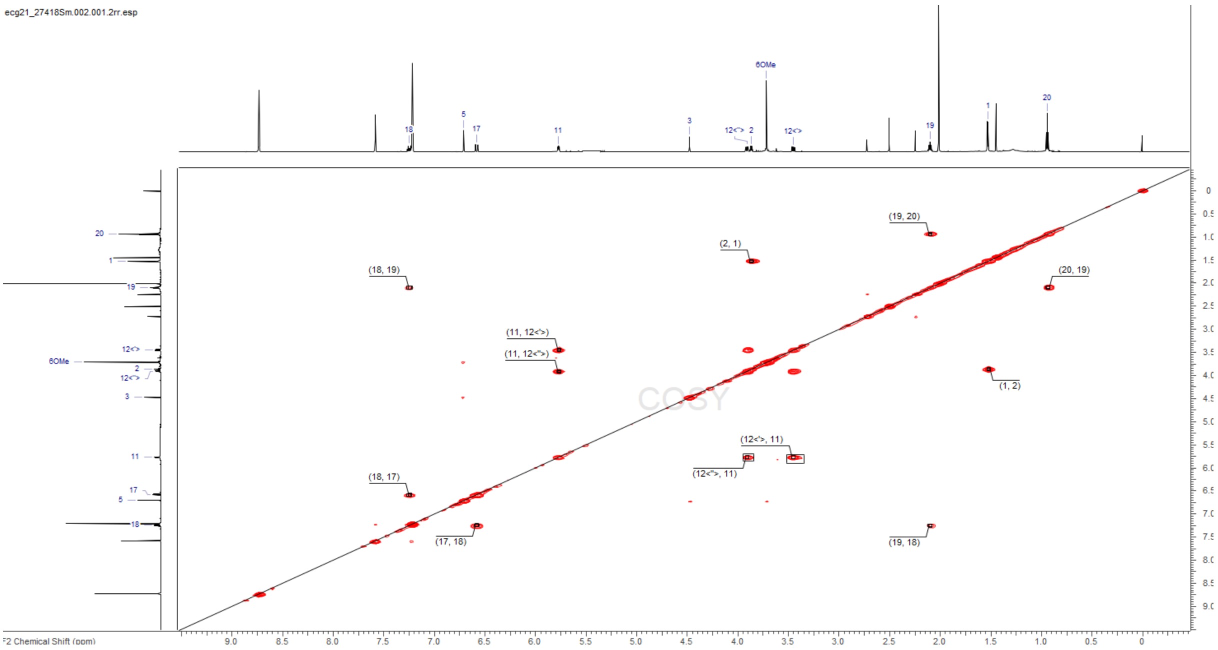

Figure S37. COSY NMR spectrum (700 MHz, pyridine-d_5) of liehnwalide D **11**.

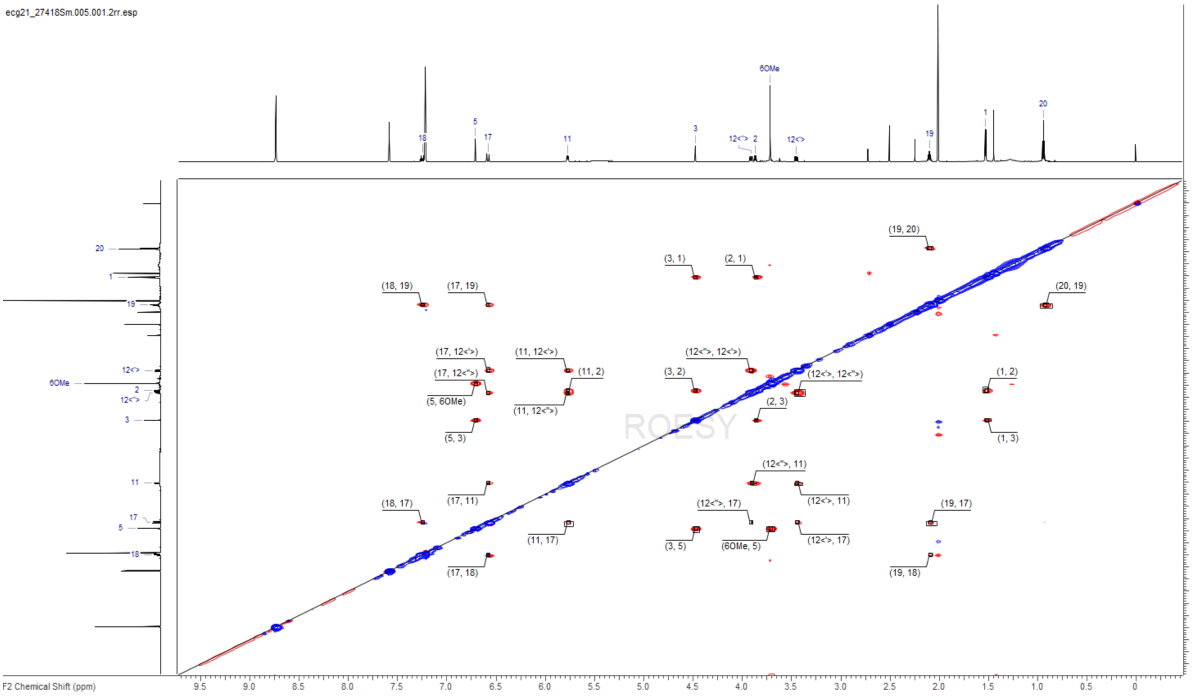

Figure S38. ROESY NMR spectrum (700 MHz, pyridine-d_5) of liehnwalide D **11**.

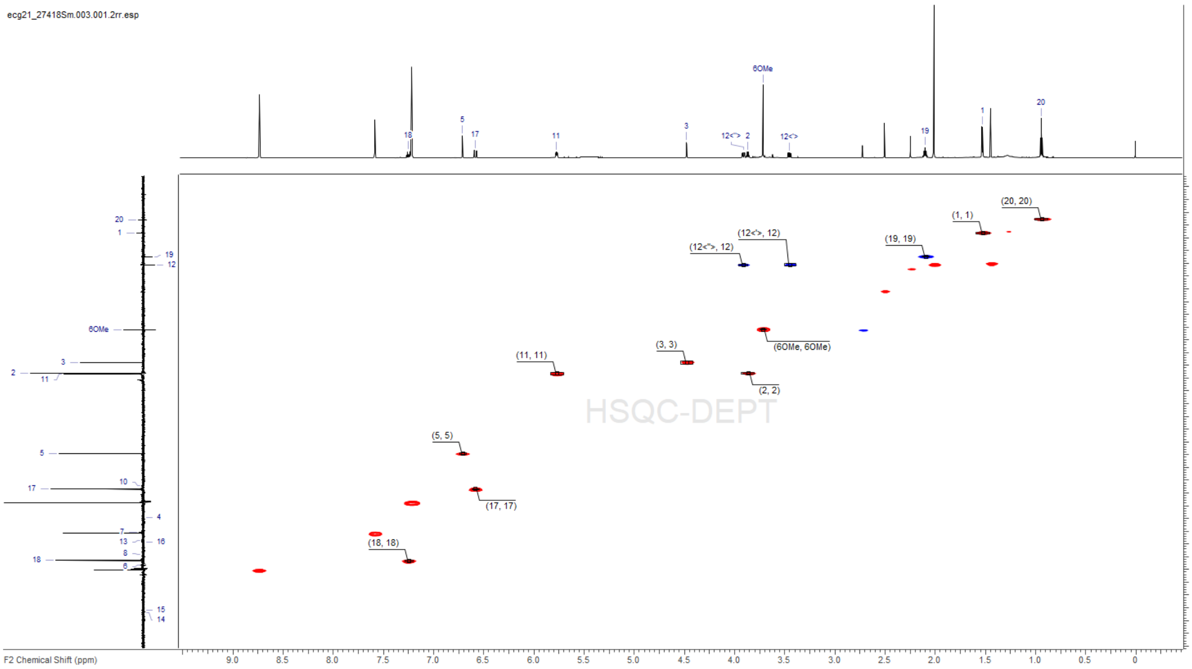

Figure S39. HSQC NMR spectrum (700 MHz, pyridine-d_5) of liehnwalide D **11**.

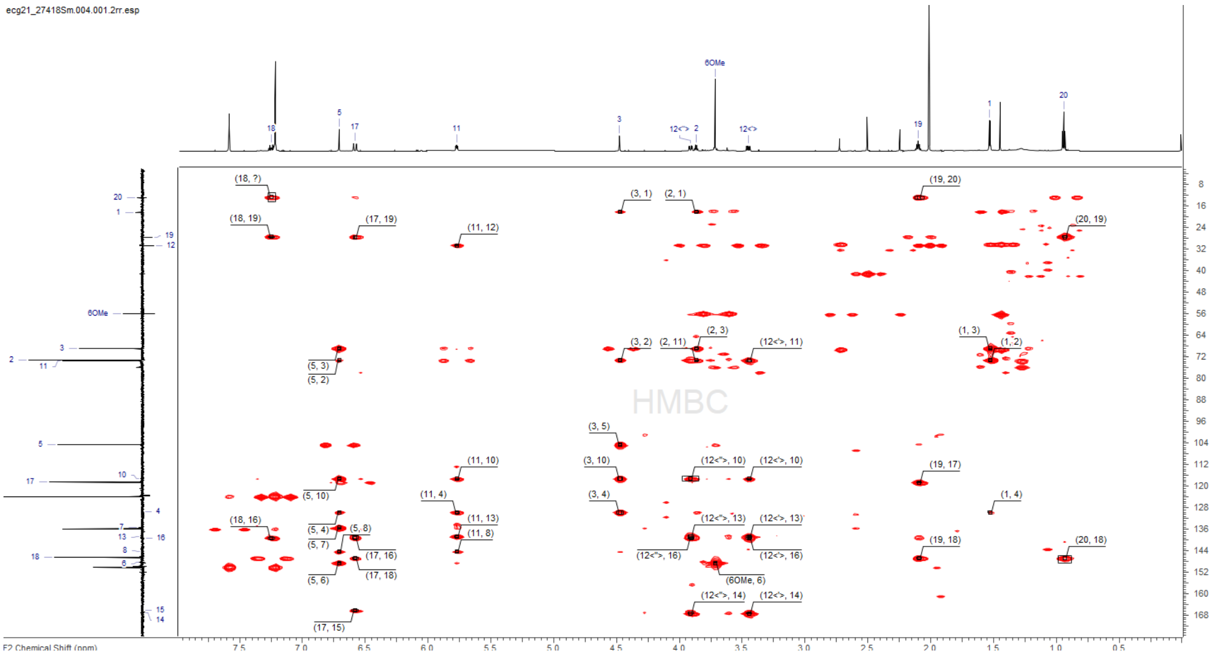

Figure S40. HMBC NMR spectrum (700 MHz, pyridine-d_5) of liehnwalide D **11**.

11.6 Compound 21a

Table S12. Chemical shifts of compound **21a** in $CDCl_3$ (600 MHz).

Position	C- type	δ_C/ppm	δ_H/ppm (mult, *J*/Hz)	COSY	HMBC	literature[166]	
						δ_C/ppm	δ_H/ppm
**1**	CH_3	12.6	1.14 (t, 7.42)	2	2, 3	12.5	1.11
**2**	CH_2	27.6	2.35 (p, 7.36)	1, 3, 4	1, 3, 4	19.2	2.35
**3**	CH	150.5	7.31 (m)*	2, 4	1, 2, 5	116.4	7.27
**4**	CH	116.3	6.35 (d, 15.89)	2, 3	2, 3, 5, 6, 8	150.2	6.31
**5**	C	138.5				165.9 or 164.3 or 138.4 or 136.3	
**6**	C	165.9					
**7**	C	164.2					
**8**	C	136.1					
**9**	CH_2	19.3	2.85 (t, 7.25)	10	5, 7, 8, 10, 11	31.3 or 27.5	2.79
**10**	CH_2	31.0	2.76 (t, 7.25)	9	8, 9, 11		2.79
**11**	C	176.4				-	
							** overlapped by solvent peak*

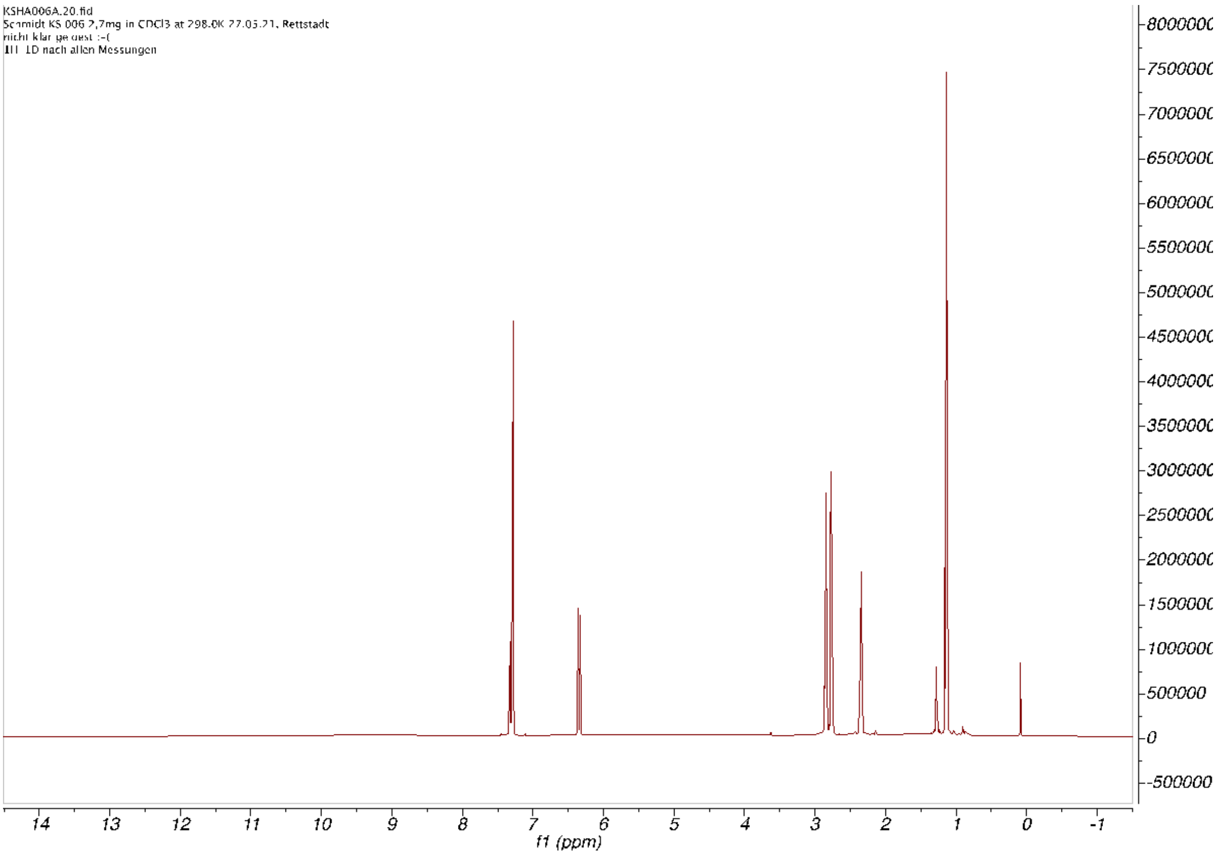

Figure S41. ^1H NMR (600 MHz) of compound **21a** in $CDCl_3$.

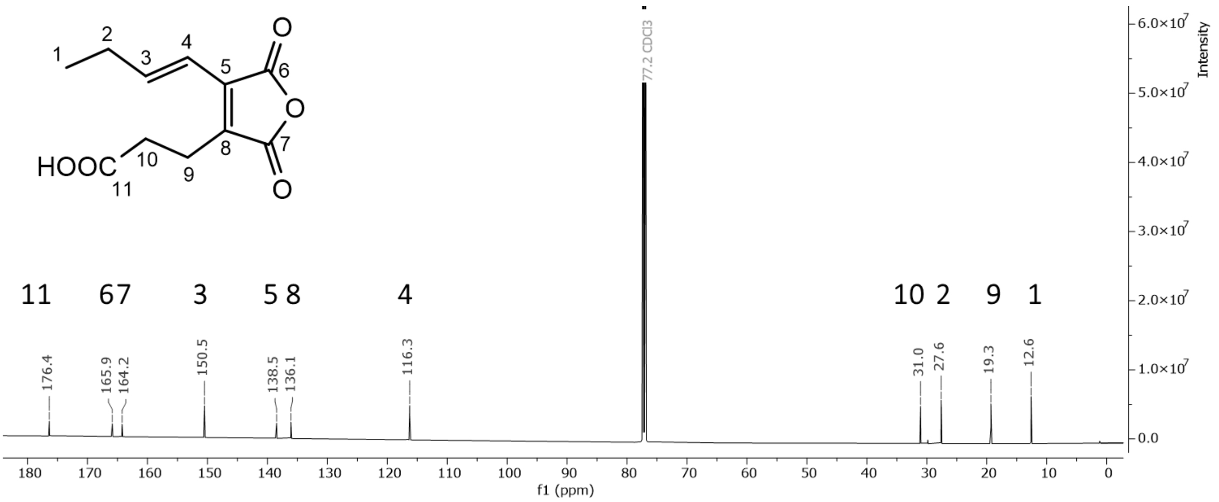

Figure S42. ^{13}C NMR (150 MHz) of compound **21a** in $CDCl_3$.

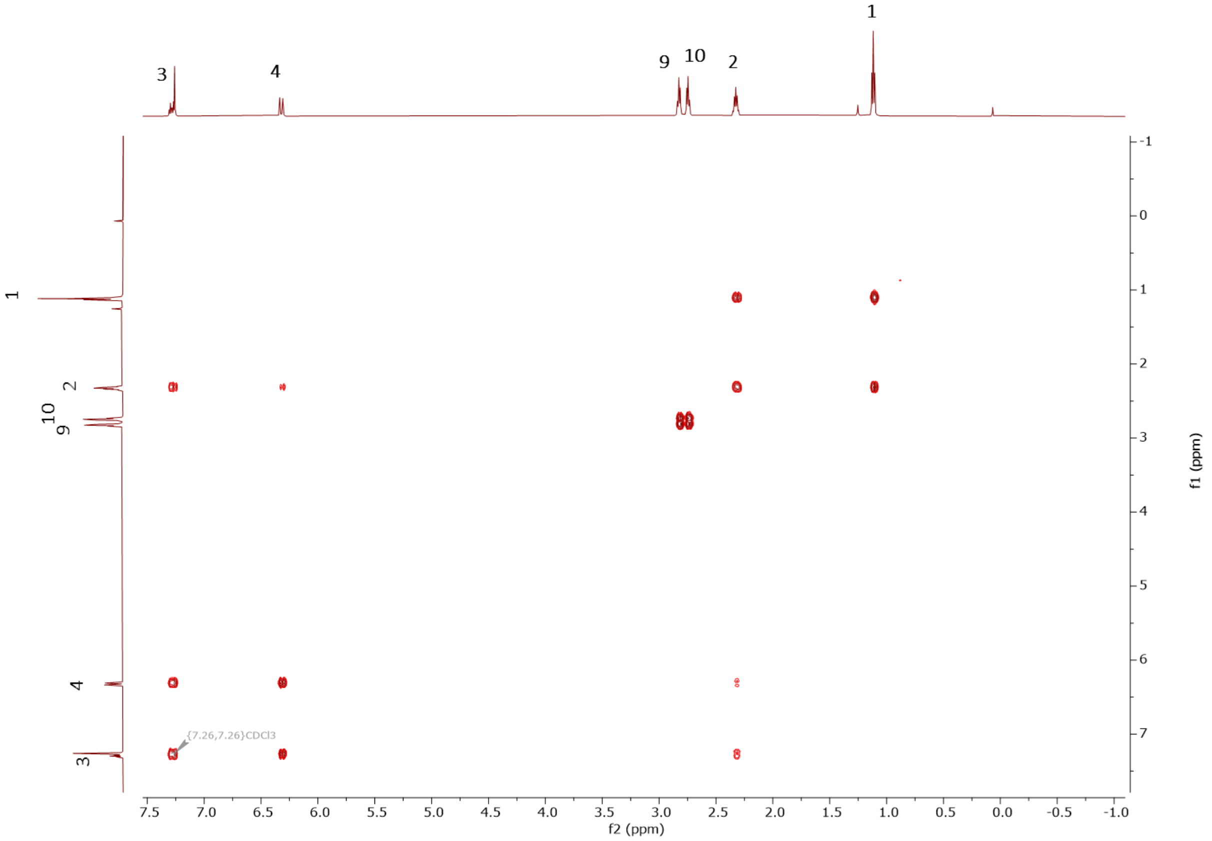

Figure S43. COSY (600 MHz) of compound **21a** in $CDCl_3$.

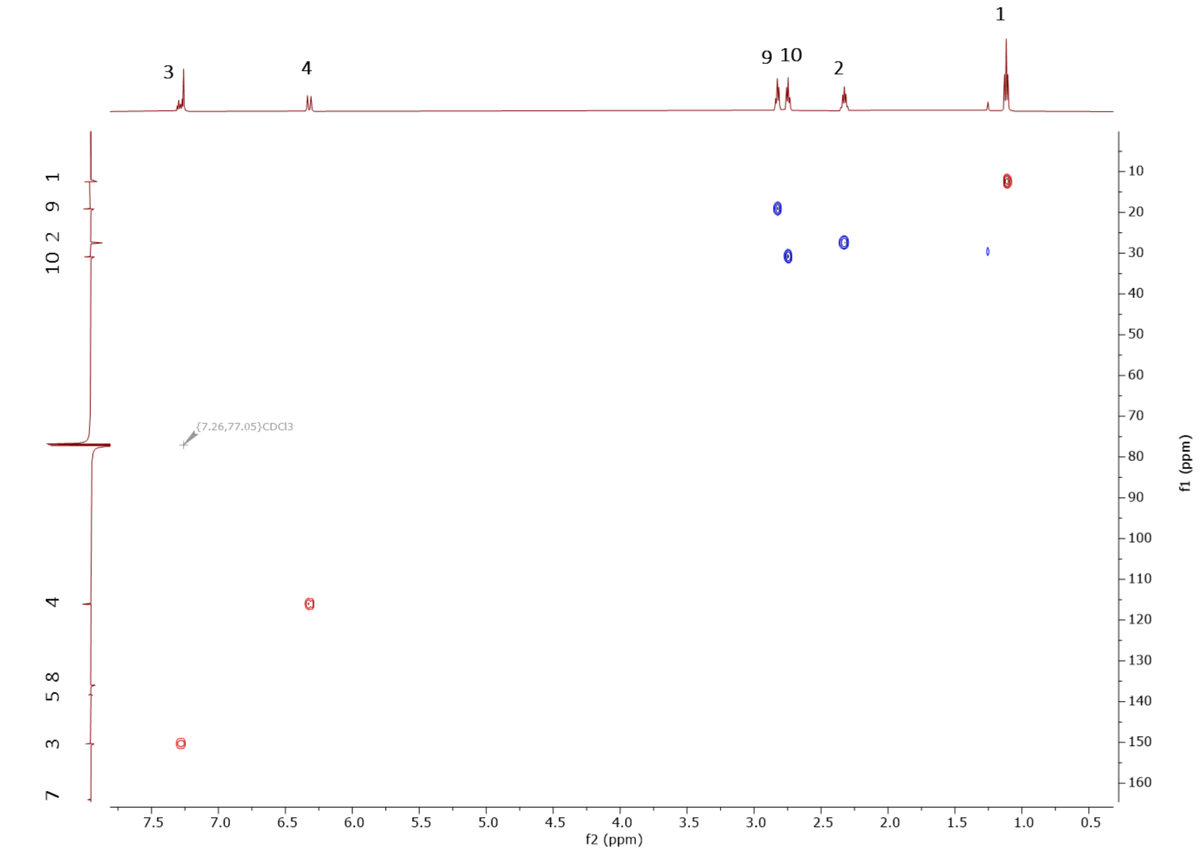

Figure S44. HSQC (600/150 MHz) of compound **21a** in $CDCl_3$.

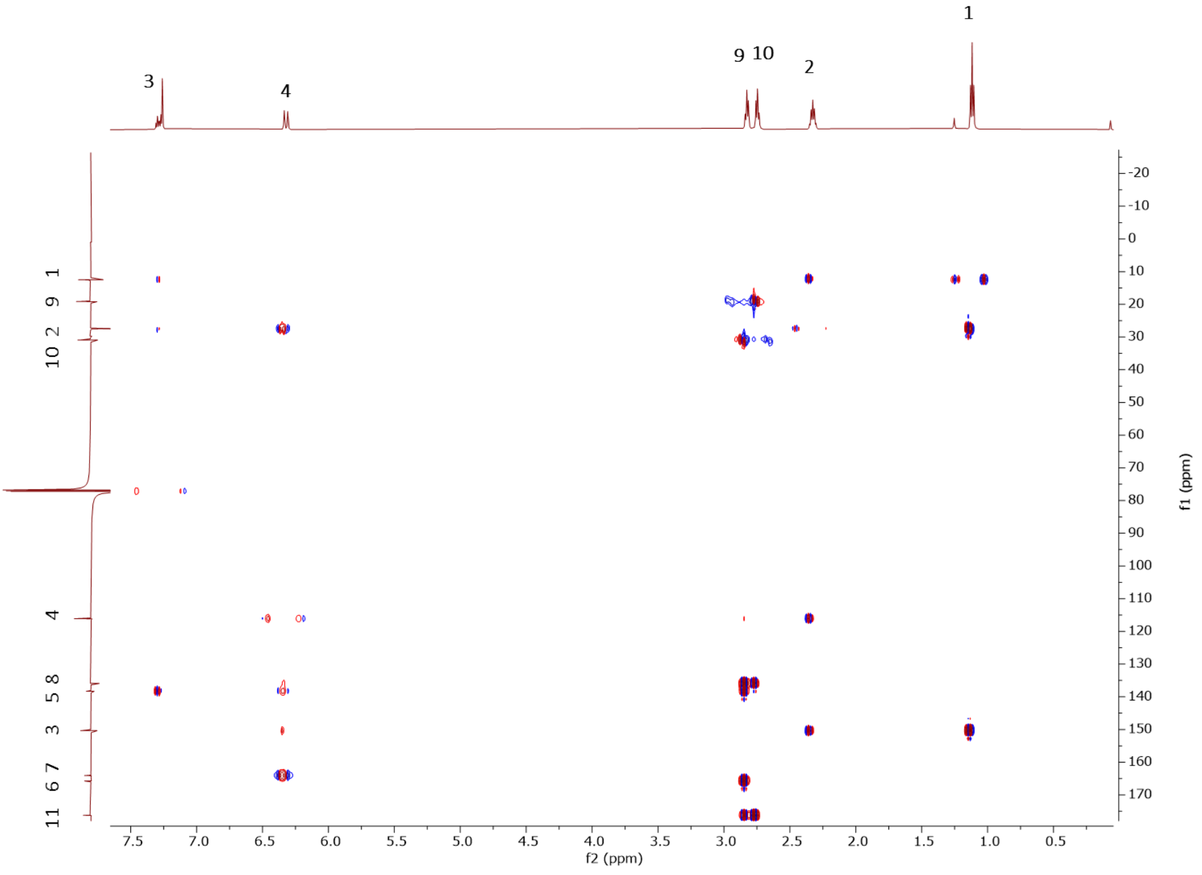

Figure S45. HMBC (600/150 MHz) of compound **21a** in $CDCl_3$.

12. LCMS traces for *A. oryzae lwm* BGC Transformants

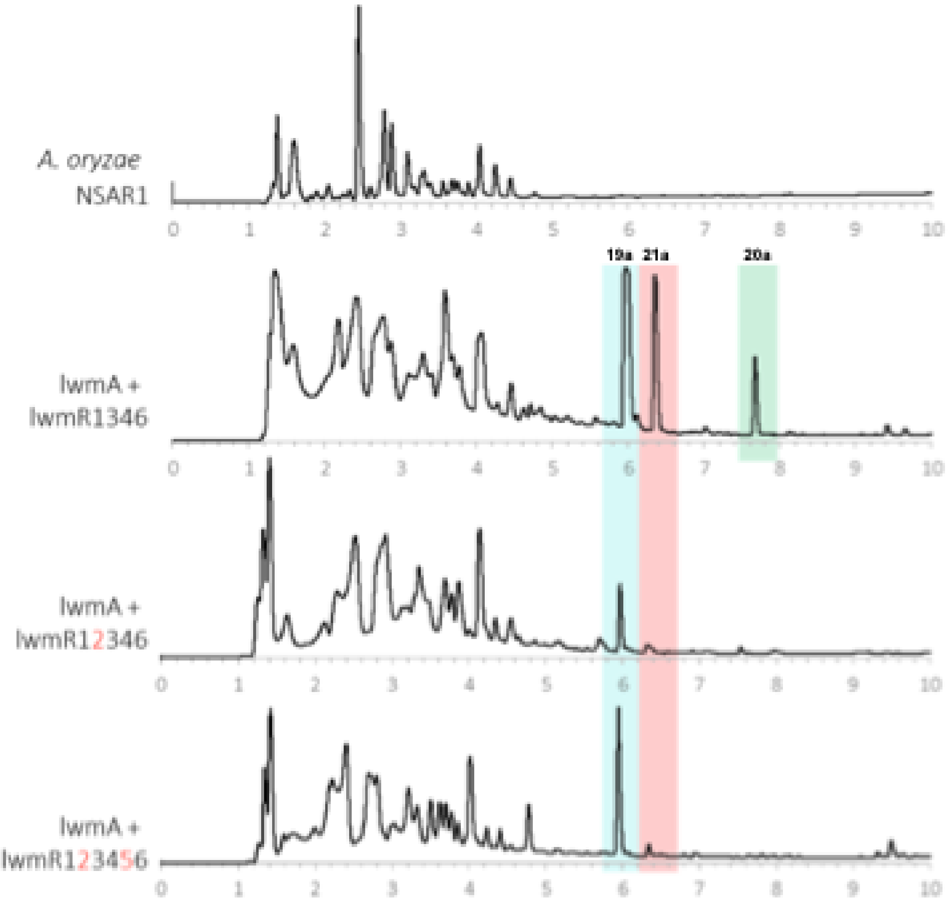

Figure S46. DAD chromatograms of heterologous co-expression of core enzymes + *lwmR2* (+ *lwmR5*)**.**

Table S13. Summary of *lwm* –BGC pathway *in vivo* and *in vitro* experiments.

	***lwm* genes**							**detected compounds**		
	**A**	R1	R2	R3	R4	R5	R6	**19a**	**20a**	**21a**
**A**	✓	✓	-	✓	✓	-	-	✓	-	-
**B**	✓	✓	-	✓	✓	-	✓	✓	✓	✓
**C**	✓	✓	✓	✓	✓	-	✓	✓	-	✓
**D**	✓	✓	✓	✓	✓	✓	✓	✓	-	✓
**E** (*in vitro*)					✓			**18a**		

13. Cloning, expression and purification of LwmR4; *in vitro* assays & analysis

13.1 Chemical Synthesis of *E*-Hex-2-enoyl CoA[8,11]

E-hex-2-enoic acid (6 mg, 0.052 mmol) was dissolved in 1.5 mL anhydrous CH_2Cl_2. After cooling to 0 °C, 5.5 µl (0.052 mmol) ethylchloroformate was added and incubated for 2 h. The CH_2Cl_2 was removed and 1.5 mL DMF was added. Coenzyme A trilithium salt (21.4 mg, 0.026 mmol) dissolved in 1.5 mL aqueous $NaHCO_3$ was added to the reaction mixture at room temperature. After 10 minutes 100 µL formic acid was added for acidification. The reaction mixture was then added to 50 mL water and frozen for the following lyophilisation (Alpha 1-4 LDplus, Martin Christ). The product (71.9 mg) was purified by preparative LCMS.

13.2 Cloning, Expression and Purification of LwmR4

The *lwmr4* sequence was codon optimized for *E. coli* (Thermo Fisher Scientific) and purchased as a synthetic fragment (Twist Bioscience). The sequence was amplified with primer overhangs appropriate for the restriction enzymes used for vector digestion (*Nde*I and *Not*I). The PCR product was digested with these restriction enzymes and ligated into pET28a(+) to create pET28a·*lwmR4*. The correct integration of the gene was verified by PCR and sequence analysis. The vector was then transformed into *E. coli* BL21 (DE3).

A 10 mL LB-media seed-culture of *E. coli* BL21(DE3) pET28a·*lwmR4* with the appropriate antibiotic (kanamycin) was inoculated with transformed cells. The culture was incubated at 37 °C and 200 rpm for 12–16 h. LB media (1.0 litre) in a 2 l shake flask with antibiotic was inoculated using the seed culture and incubated at 37 °C and 220 rpm. At an OD600 of 0.25 the temperature was decreased to 16 °C. At OD_{600} of 0.6 the culture was inoculated with IPTG (isopropyl β-D-1-thiogalactopyranoside, 250 µM) and incubated for another 16 h at 170 rpm and 16 °C. The cells were harvested by centrifugation at 6000 x g for 15 min (Thermo Scientific™ Sorvall LYNX 6000 Superspeed Centrifuge). The obtained cell pellet was homogenized in 20 mL of appropriate lysis buffer (phosphate buffer or Tris-HCl buffer). The cell disruption was achieved by sonication (SONOPULS KE76, Bandelin) for 7 min with 10 s pulse intervals and 34 % amplitude on ice. Cell debris was separated from the lysate by centrifugation for 40 min at 20000 x g at 4 °C (Thermo Scientific™ Sorvall LYNX 6000 Superspeed Centrifuge). The lysate supernatant was obtained by carefully decanting.

2 mL Ni-NTA resin was prepared according to manufacturer's instructions, added to the lysate and incubated at 4 °C for 1 h under light rotation. The mixture was carefully pipetted into CHROMABOND® empty column with PE filter element (15 mL; Macherey-Nagel) and the flow through was stored on ice for analysis with SDS-PAGE. The resin was washed with buffers including an increasing concentration of imidazole (from 20 mM up to 500 mM). Each fraction was collected and stored on ice for the analysis by SDS PAGE. The appropriate fractions were collected and concentrated using Amicon® Ultra centricons (Merck Millipore) with an appropriate cut-off for each protein. The obtained protein was stored at 20 mg/mL at -20/-80 °C, or used immediately for assay reactions. The concentration of protein solutions was determined with a DeNovix® DS-11+ Spectrophotometer. The ProtParam tool (https://web.expasy.org/protparam/) was used for determination of the protein molecular weight and extinction coefficients.

SDS-Polyacrylamide Gel Electrophoresis Samples were boiled at 95 °C for 5 min after preparing them by adding 10 µL 4 x Lämmli buffer to 30 µl of protein solution. SDS polyacrylamide gel electrophoreses were performed with 12 % polyacrylamide gels (Table S14) in combination with the gel casting system and electrophoresis (Bio-Rad). The SDS PAGE of the protein purification process (Figure S47) shows the protein in the elution buffer 2 fraction with a mass of approx. 49 kDa. This fraction was used to concentrate the enzyme to a concentration of approx. 20 µg/mL.

Table S14. Composition of 12 % SDS-polyacrylamide gel electrophoresis.

Composition	Volume (mL)	
	separating gel	stacking gel
30 % acrylamide/bisacrylamide (Rotiphorese® Gel 30 [37,5:1])	3	0.54
ddH_2O	2.45	1.7
1.5 M Tris-HCl, pH 8.8	1.9	-
0.5 M Tris-HCl, pH 6.8	-	0.25
10 % (w/v) SDS	0.075	0.002
10 % (w/v) APS	0.075	0.002
TEMED	0.0003	0.0002

The Colour Prestained Protein Standard (4 µl; Broad Range, 11–245 kDa; New England Biolabs) was used as a reference marker and 15 µl of the prepared sampled were carefully loaded on the gel. The gel was run at 75 mA for 45–60 min. Coomassie staining solution (approx. 20 mL) was used to stain the bands of the gel for approx. 1 h. Afterwards, Coomassie bleach (20 mL) was used to de-stain the gel and visualize the bands. The gel was incubated for 20–30 min repeatedly, until the protein bands were clearly visible. The gels were scanned with the Molecular Imager Gel Doc XR+ system (Bio-Rad).

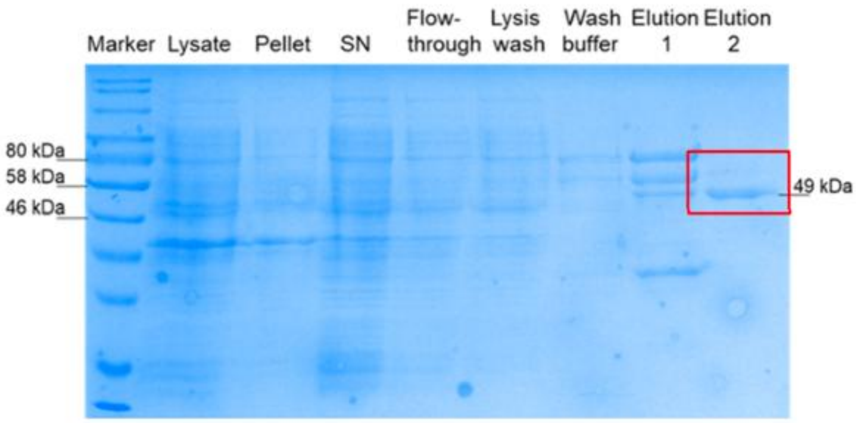

Figure S47 SDS PAGE of the purification process of *H. liehwacheense* alkylcitrate synthase from *E. coli* BL21 (SN = supernatant).

13.3 Assay of LwmR4 with Acyl-CoAs and α-keto acids

Assays were set up with components and concentrations as shown in Table S13.3.1 in a total volume of 100 µl. *E*-hex-2-enoyl CoA **16a** was synthesised as described in section 13.1. Hexanoyl CoA **16b** was obtained from Sigma-Aldrich. The assay mixtures were incubated at 30 ˚C for 2 h. Acetonitrile (100 µl) was added to stop the reaction. Protein was precipitated by centrifugation for 10 min at 21000 x g. The supernatant was directly analysed by LCMS (25 µl injection).

Table S15. Composition of reaction mix for citrate synthase assays.

Concentration	Composition
1 mM	hexanoyl-CoA **16b** or hexenoyl-CoA **16a**
1 mM	oxaloacetic acid **17** or α-ketoglutaric acid **22**
4 µM	protein
ad 100 µl	phosphate assay buffer, 50 mM pH 7.0

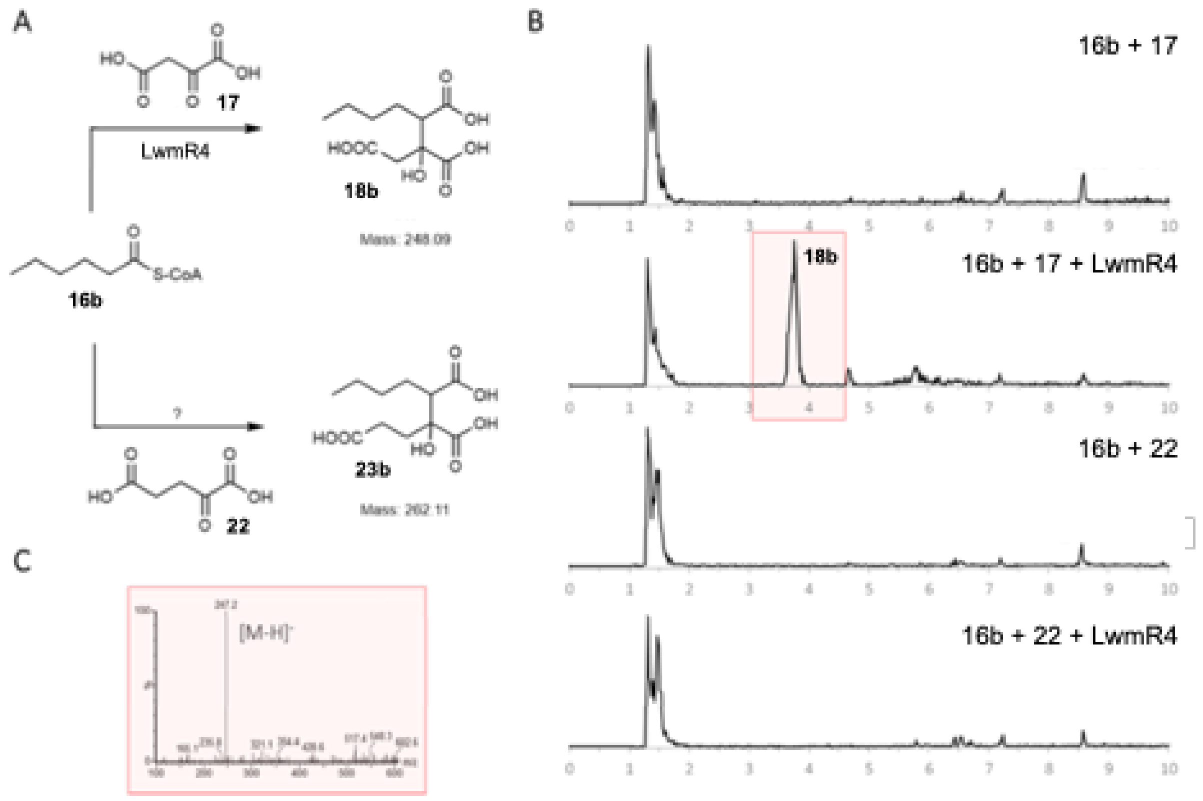

Figure S48 *In vitro* assay with *H. lienhwacheense* alkylcitrate synthase and hexanoyl-CoA: **A**, expected reaction of citrate synthase with hexanoyl-CoA; **B,** ES- chromatogram of *in vitro* assay with citrate synthase; **C**, ES- spectra of the peak at 3.7 min.

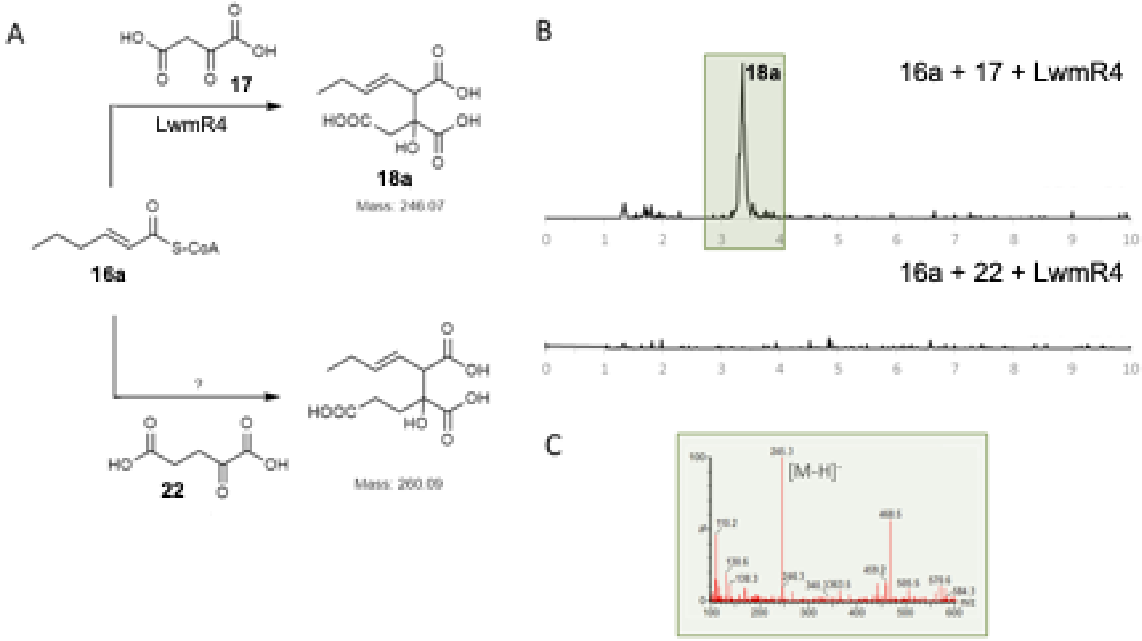

Figure S49. *In vitro* assay results with *H. lienhwacheense* alkylcitrate synthase and hexenoyl-CoA **16a**: **A**, Expected reaction of citrate synthase with hexenoyl-CoA **16a**: **B**, ES$^-$ chromatogram of *in vitro* assay with citrate synthase with EIC for expected product; **C**, ES$^-$ spectra of the peak at 3.2 min**.**

14. Biological Testing

14.1 Antimicrobial and Cytotoxicity Assays

For compounds (**5**–**11**) except for **7** the antimicrobial activity was evaluated by determining the minimum inhibitory concentration (MIC) against five fungi (*Candida albicans*, *Mucor hiemalis*, *Rhodotorula glutinis*, *Schizosaccharomyces pombe*, and *Wickerhamomyces anomalus*), different Gram-positive (*Bacillus subtilis*, *Mycolicibacterium smegmatis*, and *Staphylococcus aureus*), and Gram-negative (*Acinetobacter baumannii*, *Chromobacterium violaceum*, *Escherichia coli*, and *Pseudomonas aeruginosa*) bacteria following the protocols described by Harms et al.[12] Similarly, their cytotoxicity against two mammalian cell lines, i.e., human endocervical adenocarcinoma KB 3.1 and mouse fibroblasts L929, was evaluated in a 96-well plate following the protocols described by Charria-Girón et al.[13]

14.2 Antiviral Assays

To evaluate anti-CHIKV effects of lienhwalides, 2×10^4 Huh 7.5.1 cells per well were seeded in a 96-well plate and cultured overnight for better adherence. The spent culture medium was aspirated and cells incubated for 1 h with 50 µL of compound dilutions at 50 µM in Dulbecco's modified Eagle medium (DMEM). Subsequently, 50 µL of a Chikungunya wild-type (CHIKV-WT) virus suspension at multiplicity of infection (MOI) of 0.1 were added to the respective treatment wells. The cells were then incubated for 72 h at standard culture conditions of 37 °C and 5% CO_2. The cell viability percentage as an indirect measure of the antiviral effect (cytopathic effect (CPE) reduction) was determined using a CellTiter-Glo (Promega) assay following the manufacturer's recommendations. A 0.5% dimethylsulfoxide solution served as a negative control and ribavirin as the positive control.

15. References

[1] Ju, Y.-M. & Rogers, J. D. A Revision of the genus *Hypoxylon*. Mycologia Memoir no. 20. APS Press, ST. Paul, MN (1996).

[2] Sir, E. B., Becker, K., Lambert, C., Bills, G. F., & Kuhnert, E. Observations on Texas hypoxylons, including two new *Hypoxylon* species and widespread environmental isolates of the *H. croceum* complex identified by a polyphasic approach. Mycologia, 111(5), 832–856 (2019).

[3] Klar, P. B., Krysiak, Y., Xu, H., Steciuk, G., Cho, J., Zou, X., & Palatinus, L. Accurate structure models and absolute configuration determination using dynamical effects in continuous-rotation 3D electron diffraction data. Nature chemistry, 15(6), 848–855 (2023).

[4] Palatinus, L., Brázda, P., Jelínek, M., Hrdá, J., Steciuk, G., & Klementová, M. Specifics of the data processing of precession electron diffraction tomography data and their implementation in the program PETS2.0. *Acta crystallographica Section B, Structural science, crystal engineering and materials*, *75*(Pt 4), 512–522 (2019).

[5] Petříček, V., Palatinus, L., Plášil, J. & Dušek, M. Jana2020 – a new version of the crystallographic computing system Jana . *Zeitschrift für Kristallographie - Crystalline Materials*, *238*(7-8), 271-282 (2023).

[6] Surup, F., Wagner, O., Frieling, J., Schleicher, M., Oess, S., Müller, P., & Grond, S. The iromycins, a new family of pyridone mMetabolites from Streptomyces sp. I. Structure, NOS inhibitory activity, and biosynthesis. *The Journal of Organic Chemistry, 72* (14), 5085-5090 (2007).

[7] Pahirulzaman, K. A. K., Williams, K. & Lazarus, C. M. *A toolkit for heterologous expression of metabolic pathways in Aspergillus oryzae*. *Methods in Enzymology* vol. 517 (Elsevier Inc., 2012).

[8] Yin, S., Friedrich, S., Hrupins, V. & Cox, R. J. *In vitro* studies of maleidride-forming enzymes. *RSC Adv.* **11**, 14922–14931 (2021).

[9] Gietz, R. D. & Schiestl, R. H. Quick and easy yeast transformation using the LiAc/SS carrier DNA/PEG method. *Nat. Protoc.* **2**, 35–37 (2007).

[10] Gietz, R. D. & Woods, R. A. Yeast transformation by the LiAc/SS Carrier DNA/PEG method. *Methods Mol. Biol.* **313**, 107–120 (2006).

[11] Bond-Watts, B. B., Weeks, A. M. & Chang, M. C. Y. Biochemical and structural characterization of the trans-enoyl-coa reductase from *Treponema denticola*. *Biochemistry* **51**, 6827–6837 (2012)

[12] Harms, K., Surup, F., Stadler, M., Stchigel, A. M., & Marin-Felix, Y. Morinagadepsin, a Depsipeptide from the Fungus *Morinagamyces vermicularis* gen. et comb. nov. *Microorganisms*, *9*(6), 1191 (2021).

[13] Charria-Girón, E., Stchigel, A. M., Čmoková, A., Kolařík, M., Surup, F., & Marin-Felix, Y. *Amesia hispanica* sp. nov., Producer of the Antifungal Class of Antibiotics Dactylfungins. *Journal of fungi (Basel, Switzerland)*, *9*(4), 463 (2023).
